# Induction of ferroptosis and mitochondrial dysfunction by oxidative stress in PC12 cells

**DOI:** 10.1038/s41598-017-18935-1

**Published:** 2018-01-12

**Authors:** Chuanhong Wu, Wenwen Zhao, Jie Yu, Shaojing Li, Ligen Lin, Xiuping Chen

**Affiliations:** 1 State Key Laboratory of Quality Research in Chinese Medicine, Institute of Chinese Medical Sciences, University of Macau, Macao, China; 2 Institute of Chinese Materia Medica, China Academy of Chinese Medical Sciences, Beijing, 100700 China

## Abstract

Neurodegenerative diseases (NDD) are typically associated with neuron loss in nervous system areas. Interventions with related death mechanisms may ameliorate NDD progression. Oxidative stress plays an important role in NDD cell death routines. However, tert-butylhydroperoxide (t-BHP), a widely used oxidative stress stimulus, induces neural cell death through a mechanism that remains elusive. In our study, the ferroptosis marker events occurred after co-treatment with 100 μM t-BHP for 1 h, all of which were reversed in the presence of the ferroptosis inhibitor ferrostatin-1 (Fer-1) and the iron chelator deferoxamine, implying the occurrence of ferroptosis. Moreover, mitochondrial dysfunction accompanied by a decreased in membrane potential and ATP production, increased mitochondrial ROS generation. Furthermore, this mitochondrial dysfunction could be reversed by Fer-1. In addition, JNK1/2 and ERK1/2 were activated upstream of the ferroptosis and mitochondrial dysfunction. In summary, these data suggest that ferroptosis, coupled with mitochondrial dysfunction, was involved in t-BHP-induced PC12 death. JNK1/2 and ERK1/2 played important roles in t-BHP-induced cell death. Overall, this study might provide clues to the oxidative stress-based strategies for cell protection in NDD.

## Introduction

Cell death and proliferation control the homeostatic balance of metazoans. Precise control of the key nodes of death plays a pivotal role in the control of this homeostatic balance, which produces disease pathologies such as neurodegenerative diseases (NDD), once disturbed^1^. However, cell death induced by different environmental stresses or intracellular disorders exhibits diverse morphological and biochemical characteristics, which are defined as distinguished types of cell death. For several years, the first-discovered form of regulated cell death, called apoptosis, was considered the only therapeutically tractable mechanism^2^. However, this long-standing paradigm in the field has been challenged and revised in recognition of other regulated necrotic forms of cell death, such as programmed necrosis, ferroptosis, parthanatos and cyclophilin D-dependent necrosis^3^. In addition, several non-apoptotic forms of cell death lack a clear necrotic phenotype, such as the neutrophil extracellular-trap-associated cell death^4^, pyroptosis and autophagic cell death^5,6^. The identification of a specific type of cell death is crucial for prevention when homeostatic balance is disturbed.

Oxidative stress is a well-accepted paradigm that mediates neuronal dysfunction and death in age-related neurodegenerative diseases (NDD) such as Alzheimer’s disease and Parkinson’s disease. According to investigations of the underlying mechanisms involved in different paradigms of neurodegeneration, deregulated intracellular oxidative stress was identified as a common triggers of death signaling in neurons^7^.

Ferroptosis was identified more recently to be involved in oxidative stress-induced cell death^8^ and it is introduced as an iron-dependent form of oxidative cell death in cancer cells and in neurons. The paradigm of ferroptosis involves the generation of soluble and lipid ROS through iron-dependent enzymatic reactions. Further, mechanisms of ferroptosis have been identified in neurons, in death signaling pathways induced by cerebral ischemia^9^, and in glutamate-induced neurotoxicity in organotypic hippocampal slice cultures^10^. The biochemical mechanisms linking oxidative stress to mitochondrial dysfunction in ferroptosis have been elucidated and identified that Bid plays an important role in this process^7^.

The PC12 cell line is used as a model system for neurobiological and neurochemical studies^11–18^. This study was designed to explore the cell death type induced by *tert*-butylhydroperoxide (t-BHP), a widespread inducer of oxidative stress stimuli since 1985^19–21^ in PC12 cells, which might provide clues for oxidative stress-based strategies for cell protection in NDD.

## Results

### Selection of the oxidative stress condition in PC12 cells

**Figure 1:**
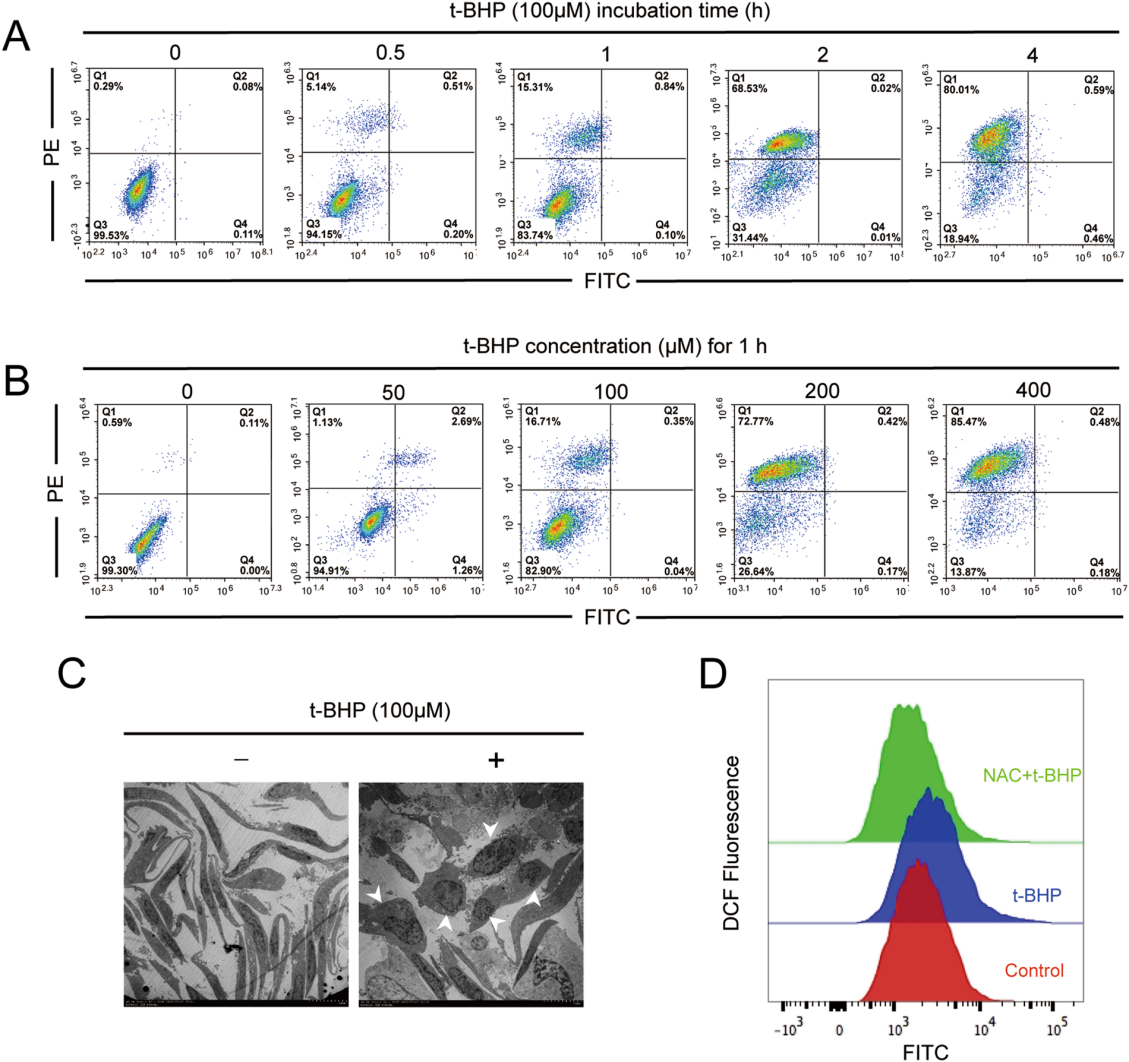
Selection of the oxidative stress condition in PC12 cells. PC12 cells were treated with t-BHP (50 μM, 100 μM, 200 μM and 400 μM) for 0.5 h, 1 h, 2 h and 4 h. Annexin V/PI staining (**A** and **B**), cell morphology (**C**) and ROS generation were determined (**D**). NAC, N-acetyl-_L_-cysteine.

**Figure 2:**
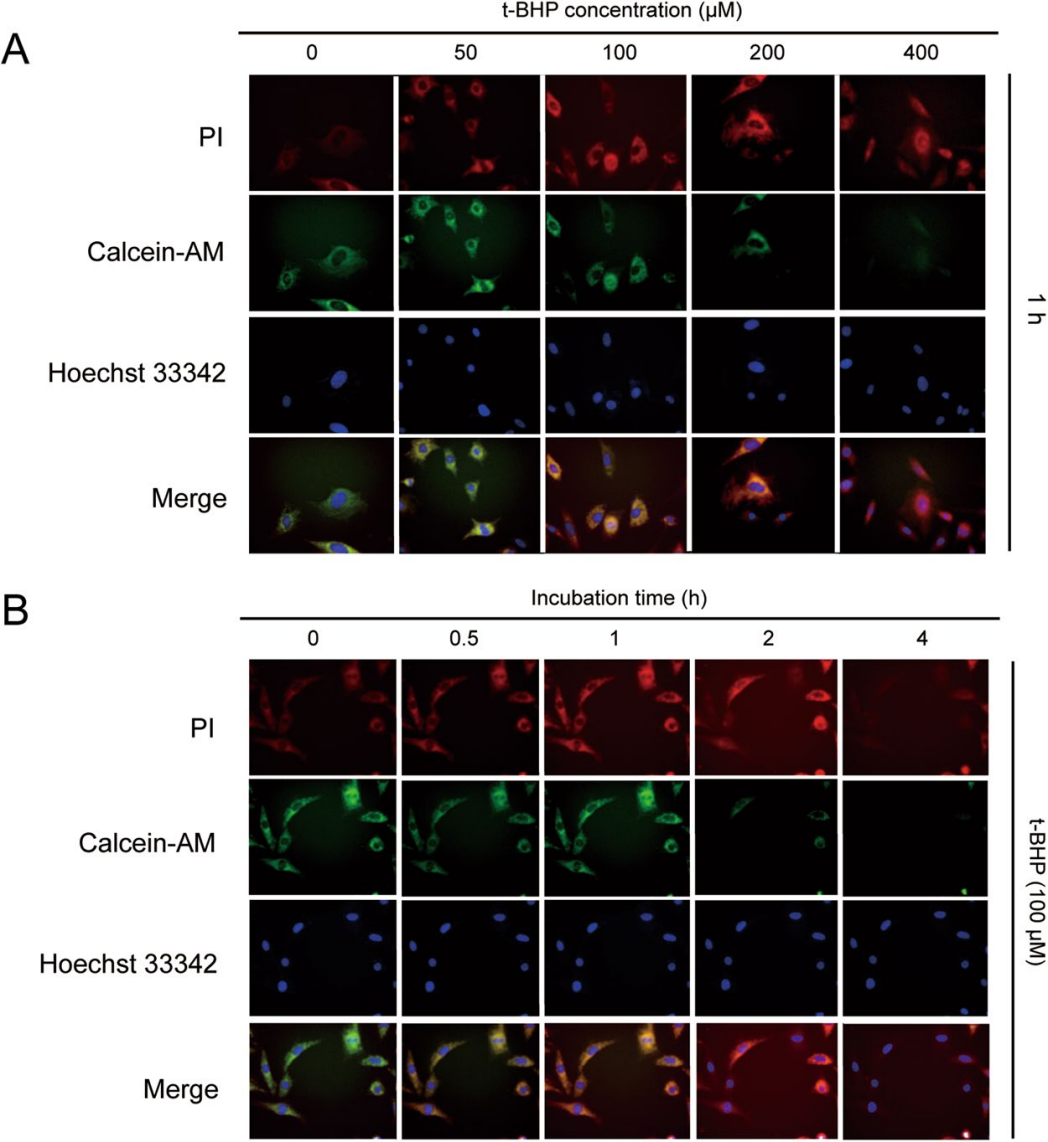
Fluorescence detection for the living and dead cells. PC12 cells were treated with t-BHP (50 μM, 100 μM, 200 μM and 400 μM) for 0.5 h, 1 h, 2 h and 4 h, and then the fluorescence images were detected by EVOS FL auto 2.

PC12 cells were treated with t-BHP (50 μM, 100 μM, 200 μM and 400 μM) for 0.5 h, 1 h, 2 h and 4 h. Then, the Annexin V/PI staining results revealed that PC12 cells that were co-treated with t-BHP (100 μM) for 1 h yielded ~15–16% cells death, and lower concentrations of t-BHP (50 μM to 100 μM) caused little apoptosis. However, when t-BHP was used at high concentrations (200 μM and 400 μM) and co-treated for longer than 1 h, the cell death percentage increased to as high as 85% (Fig. 1A and B). The electron microscopy images revealed condensed nuclei and cytoplasm, accompanied by cell rounding, in PC12 cells that were treated with t-BHP at a concentration of 100 μM for 1 h (Fig. 1C). Fluorescence images of PC12 cells stained with PI and Calcein-AM, indicated that PC12 cells treated with t-BHP at a concentration of 100 μM for 1 h, showed a yellow merged color, which implied that the staining of these two dyes came to a contrast balance (Fig. 2A and B). Considering that live cells remained suitable for rescue after treatment of t-BHP (100 μM) for 1 h, the same experimental conditions were selected for further study. Here, as shown in Fig. 1D, PC12 cell co-treatment with t-BHP at 100 μM for 1 h, caused a remarkable increase in ROS generation, and this could be reversed by the ROS scavenger N-acetyl-_L_-cysteine (NAC, 5 mM).

### Evidence of ferroptosis in PC12 cells after co-treatment with t-BHP

**Figure 3:**
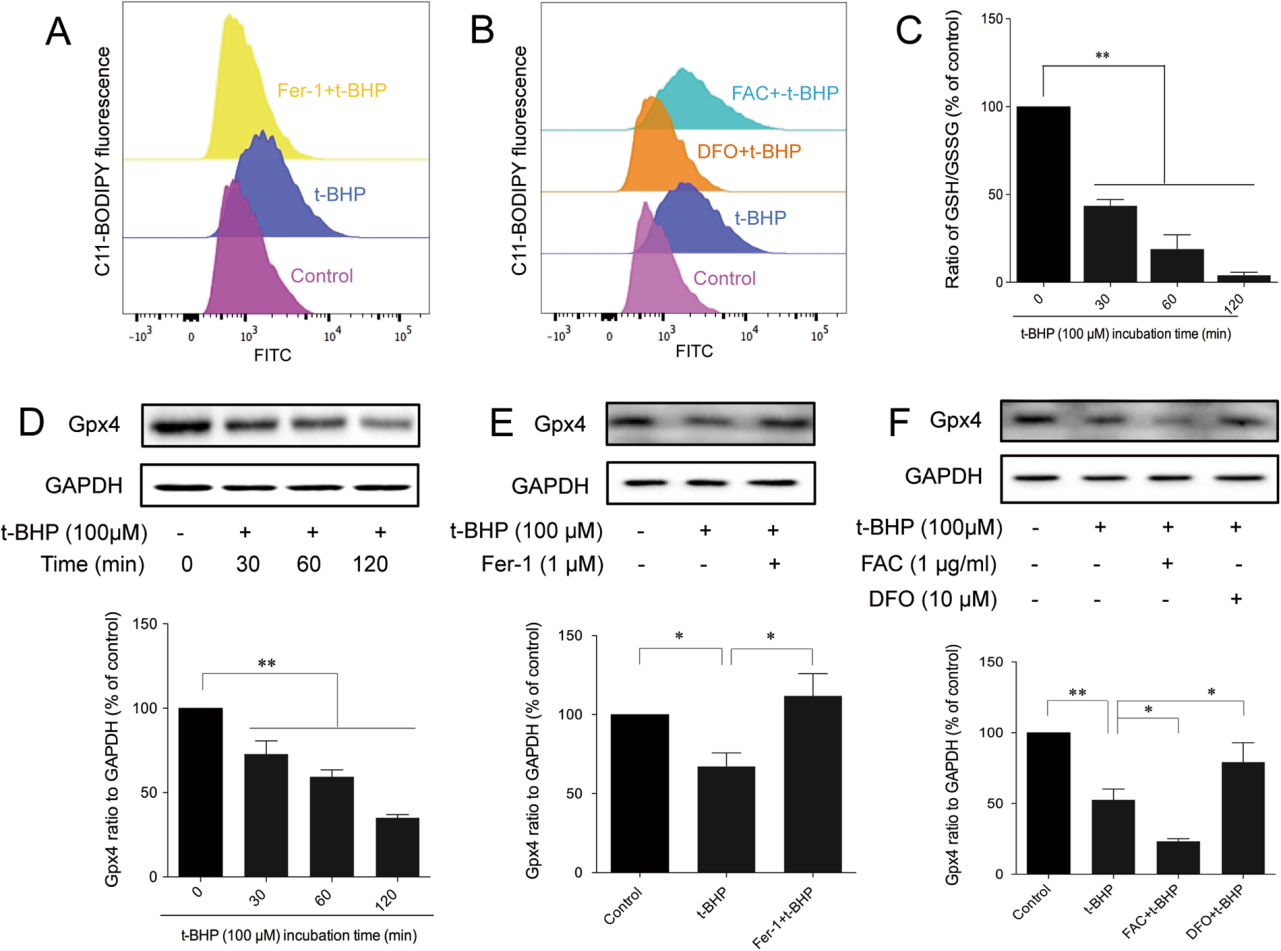
Evidence of ferroptosis in PC12 cells after co-treatment with t-BHP. PC12 cells were treated with t-BHP (100 μM) for 1 h with or without Fer-1 (1 μM)/DFO (100 μM)/FAC (1 μg·ml^−1^) pretreatment for 24 h. Generation of lipid ROS (**A** and **B**) was determined by the C11-BODIPY (581/591) probe. The ratio of GSH to GSSG (I) was determined by a luminometer kit (**C**). The expression of Gpx4 (**D**–**F**) was detected by western blot analysis (the displayed blots are cropped, and the full-length blots are included in the Supplementary Information file). **P < 0.01; *P < 0.05; Fer-1, ferrostatin-1; DFO, deferoxamine; FAC, ferric ammonium citrate.

Ferroptosis is a recently coined type of cell death^10^. Lipid peroxidation^10^, down-regulated glutathione peroxidase 4 (Gpx4)^22^, and glutathione (GSH) depletion^23^ are three crucial events that occur during ferroptosis. t-BHP increased the generation of lipid ROS (Fig. 3A and B) but decreased the expression of Gpx4 (Fig. 3D–F). All of these effects could be reversed by the ferroptosis inhibitor, ferrostatin-1 (Fer-1) and an iron chelator deferoxamine (DFO), but were counteracted by the ferric ion provider ammonium ferric citrate (FAC). In addition, t-BHP could decrease the ratio of GSH/GSSG (Fig. 3C).

### Evidence of mitochondrial dysfunction in PC12 cells after co-treatment with t-BHP

**Figure 4:**
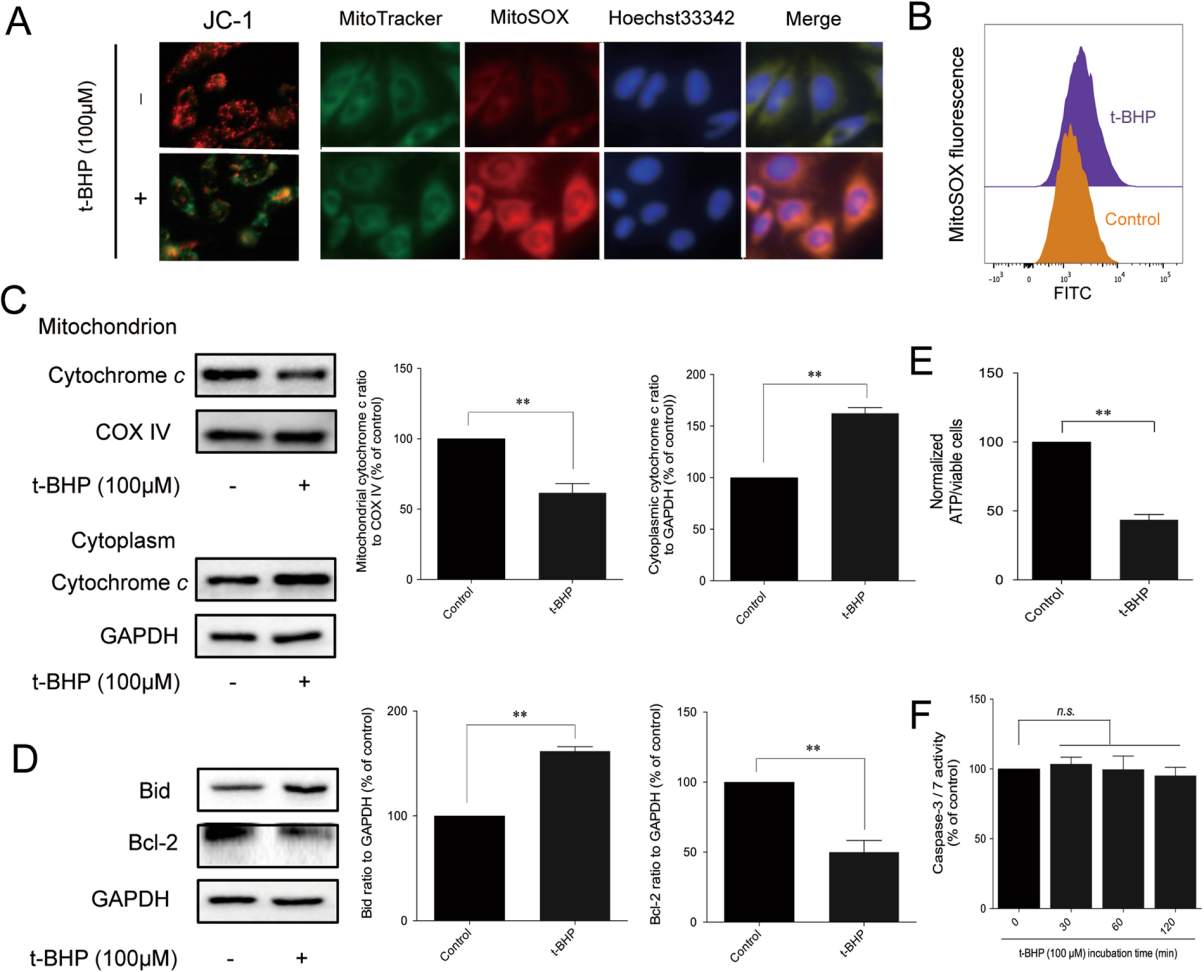
Evidence of mitochondrial dysfucntion in PC12 cells after co-treatment with t-BHP. Mitochondrial membrane potential and mitochondrial ROS (**A** and **B**) were detected by JC-1 and MitoSOX probes. ATP generation (**E**) was detected by a luminometer kit. The expression of mitochondria-related proteins (**C** and **D**) was determined by western blot analysis (the displayed blots are cropped, and the full-length blots are included in the Supplementary Information file). The activity of caspase-3/-7 was detected by a luminometer kit (**F**). **P < 0.01; *n.s*, no significance.

Biochemical pathways of apoptosis activation can be extracellular or intracellular, as well as caspase-dependent or mitochondrial-dependent^24^. To explore the role of caspases, the activity of caspase-3 and caspase-7 was detected. t-BHP had no influence on caspase-3 or caspase-7 activity (Fig. 4F). Other caspase-related proteins were also detected, but their activity was not altered by t-BHP (Figure S3). Given that caspases are not affected by t-BHP, the mitochondrial dysfunction was further detected. As shown in Fig. 4A, the JC-1 staining indicated that the mitochondrial membrane potential was significantly decreased after t-BHP treatment, as evidenced by the increased ratio of green to red fluorescence. Moreover, the mitochondrial ROS were also increased after co-treatment with t-BHP (Fig. 4A and B), and ATP production was decreased (Fig. 4E). Furthermore, the expression of the mitochondria-dependent pro-apoptotic protein Bid was increased, and the expression of anti-apoptotic protein Bcl-2 was decreased. In addition, cytochrome *c* was translocated from the mitochondria to the cytoplasm (Fig. 4C and D). Collectively, these results suggested that t-BHP induced mitochondrial dysfunction in PC12 cells and induced the release of apoptotic inducing protein, which led to cell death.

### Evidence against t-BHP-induced autophagy and necroptosis in PC12 cells

**Figure 5:**
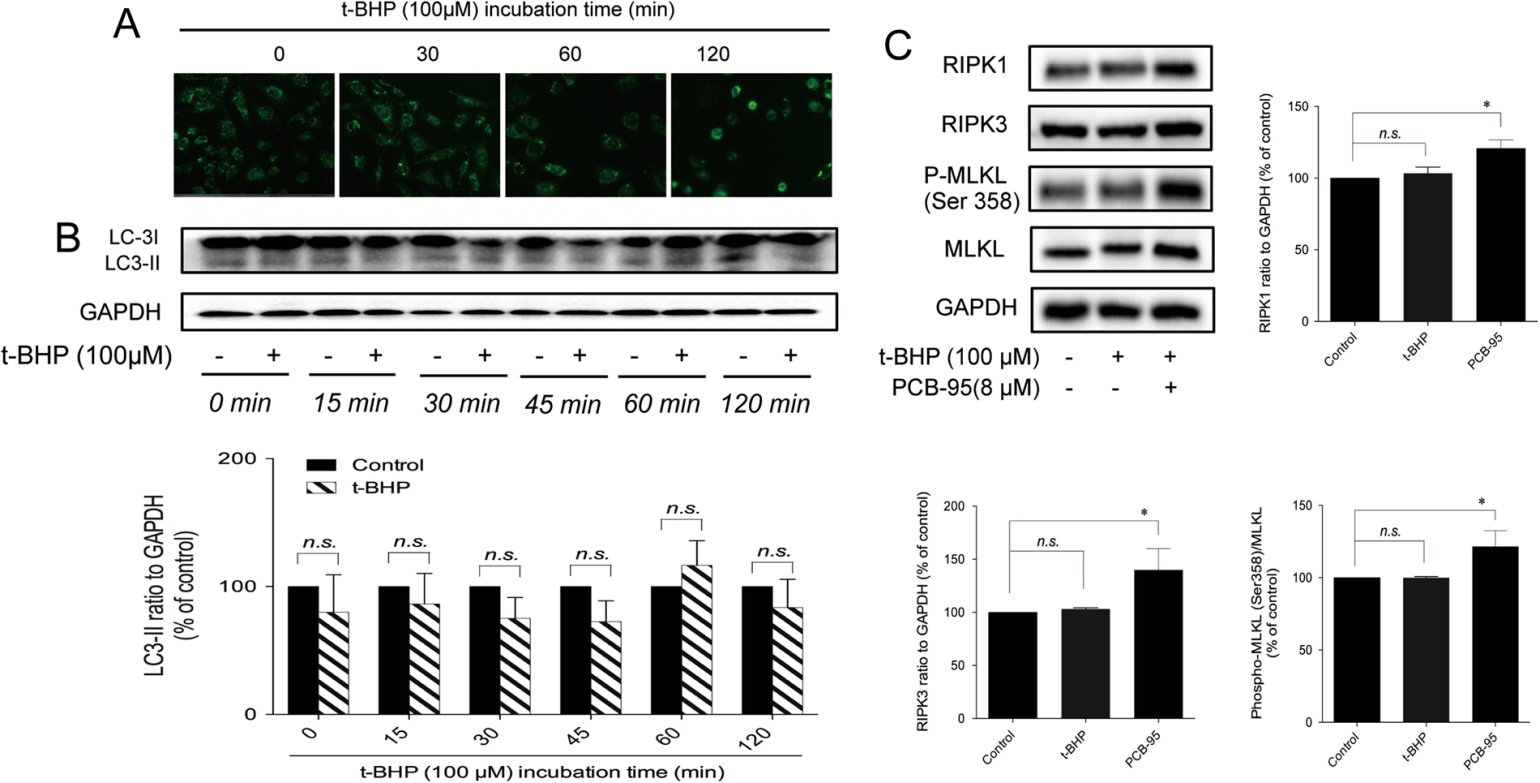
Evidence against t-BHP-induced autophagy and necroptosis in PC12 cells. PC12 cells were treated with t-BHP (100 μM) for 1 h with or without CQ (30 μM) pretreatment for 24 h. The autophagic vacuoles (**A**) and the expression of LC-3I and LC-3II (**B**) were determined by MDC staining and western blot analysis (the displayed blots are cropped, and the full-length blots are included in the Supplementary Information file). The expression of necroptosis-related proteins (**C**) was determined by western blot analysis (the displayed blots are cropped, and the full-length blots are included in the Supplementary Information file). *n.s*, no significance.

Autophagy is a non-apoptotic form of programmed cell death^25^. There are reports suggesting that ferroptosis is an autophagic inducible cell death^26^. Increased fluorescence was not observed after monodansylcadaverine (MDC) staining (Fig. 5A). In addition, the protein expression of LC-3II, which is a universal biomarker for autophagy, remained unaffected by t-BHP (Fig. 5B). Therefore, autophagy was not involved in the cell death triggered by t-BHP. Necroptosis is another form of caspase-independent regulated cell death that has been implicated in the development of a range of inflammatory, autoimmune, and neurodegenerative diseases^27^. In response to the activation of TNF receptor family members, RIPK1 is recruited to the cytosolic side of the receptor and its kinase activity is activated. RIPK1 then interacts with and phosphorylates a related kinase, RIPK3, leading to its activation^28,29^. Once active, RIPK3 then phosphorylates MLKL^30^. MLKL forms oligomers and translocates to the plasma membrane, resulting in necrotic cell death^3^. The role of necroptosis was further explored in this study. The protein expression levels of RIPK1, RIPK3, *p*-MLKL (Ser358), and MLKL were all increased after treatment with the necroptosis inducer PCB-95, but were not affected by t-BHP (Fig. 5C). Therefore, necroptosis might not be involved in t-BHP-induced PC12 death.

### Ferroptosis inhibitor protected PC12 cells from mitochondrial dysfunction

In line with earlier observations, the ferroptosis inhibitor, Fer-1, prevented t-BHP -induced cell death, decreased the generation of lipid peroxidation, induced the depletion of GSH, and increased the expression of Gpx4. Then, we analyzed the effects of Fer-1 on mitochondrial integrity in the t-BHP-induced PC12 cell model.

**Figure 6:**
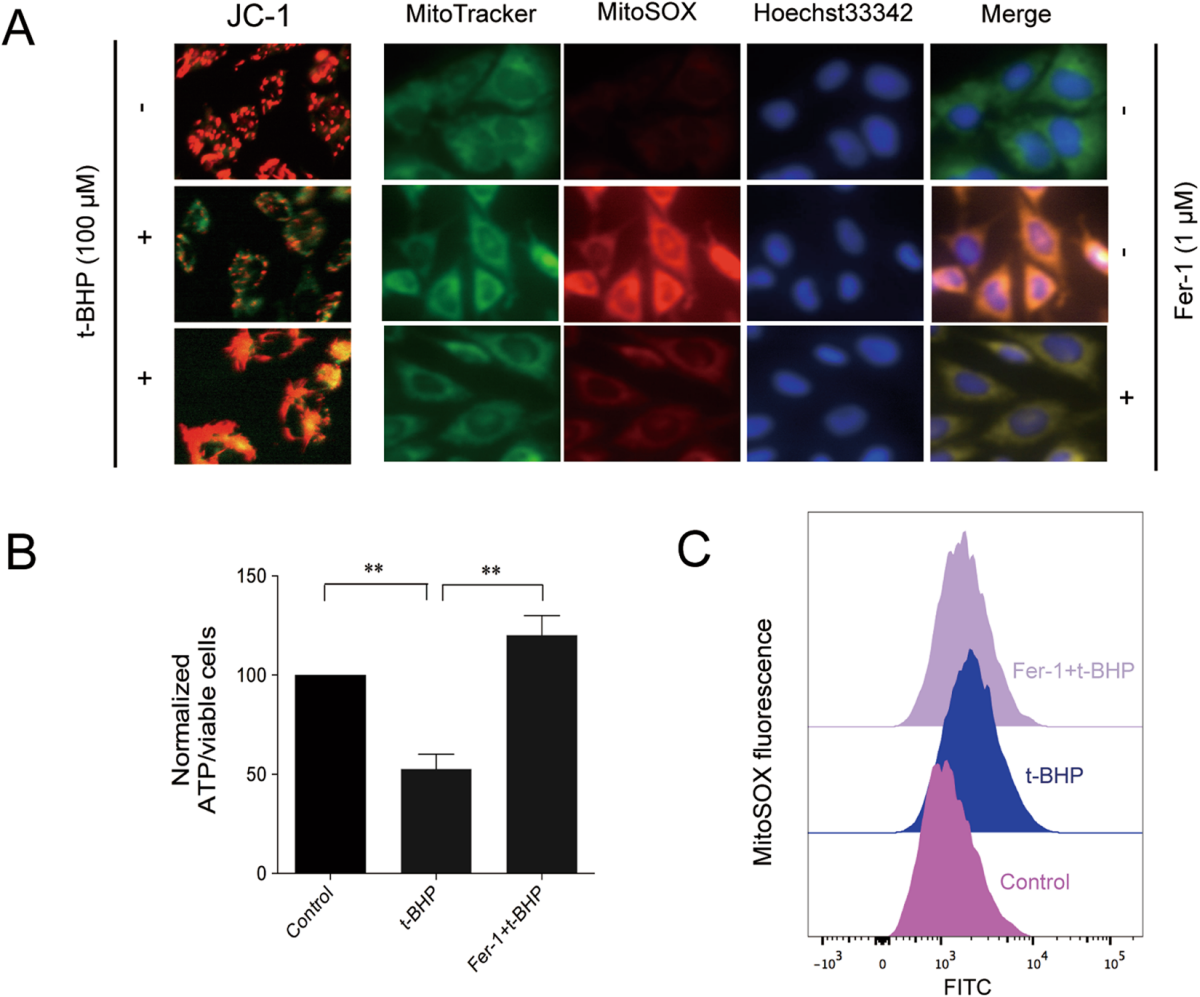
Ferroptosis inhibitor protected PC12 cells from mitochondrial dysfunction. Mitochondrial membrane potential and mitochondrial ROS (**A**–**C**) were detected by JC-1 and MitoSOX probes. ATP generation (**B**) was detected by a luminometer kit. **P < 0.01.

As shown in Fig. 6, Fer-1 increased the fluorescence ratio of red to green (Fig. 6A), as well as decreased the ATP generation (Fig. 6B) and the generation of mitochondrial ROS (Fig. 6A and C), All of these results revealed that Fer-1 preserves mitochondrial integrity.

### JNK1/2 and ERK1/2 were involved in t-BHP-induced ferroptosis and mitochondrial dysfunction

**Figure 7:**
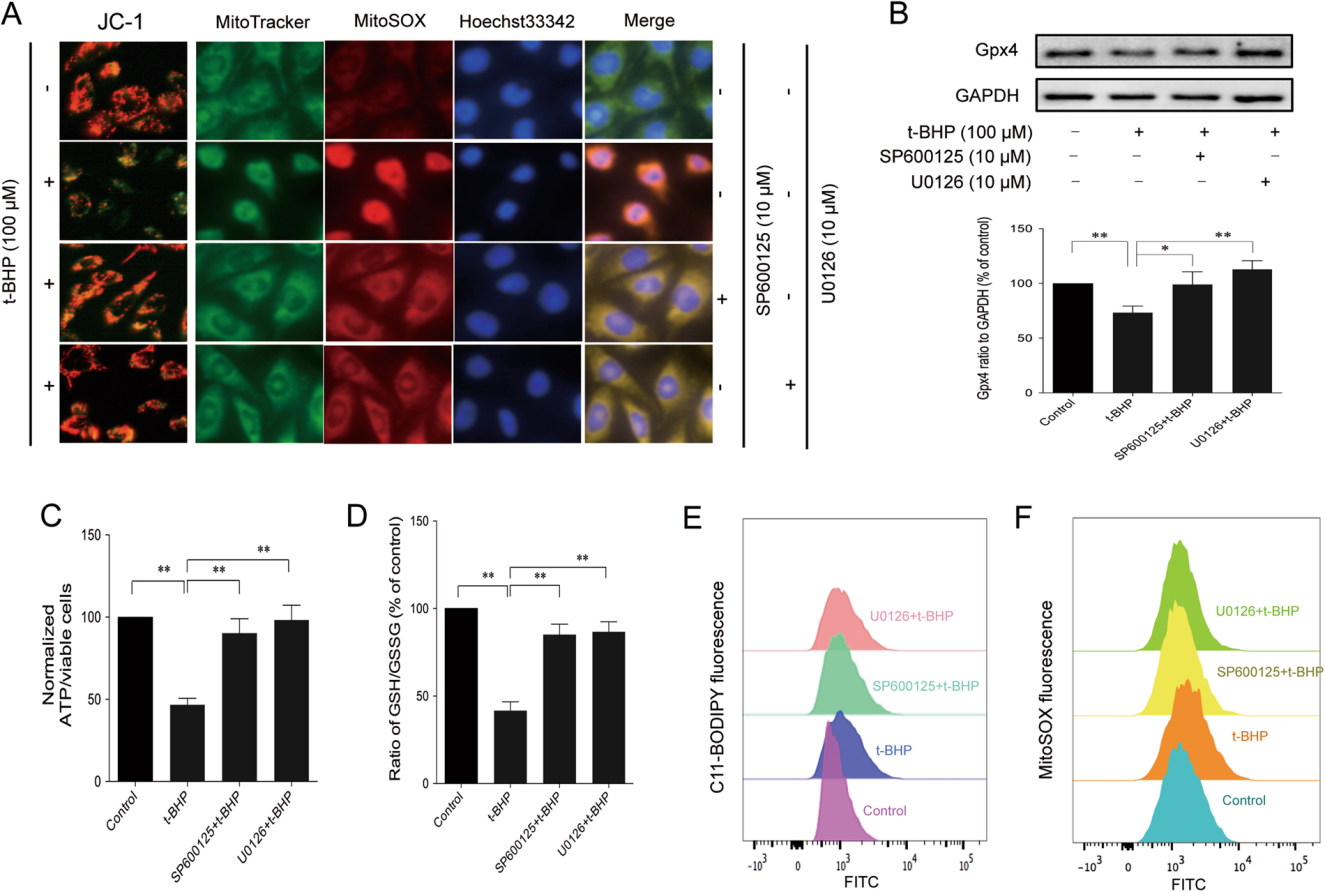
JNK1/2 and ERK1/2 inhibitors inhibited t-BHP-induced ferroptosis and mitochondrial dysfunction. PC12 cells were treated with t-BHP (100 μM) for 1 h with or without SP600125 (10 μM)/U0126 (10 μM) pretreatment for 24 h. Mitochondrial membrane potential and mitochondrial ROS (**A**–**C**) were detected by JC-1 and MitoSOX probes. ATP generation (**C**) was detected by a luminometer kit. The generation of lipid ROS (**E** and **F**), the ratio of GSH to GSSG (**D**), and the expression of Gpx4 (**B**) were determined by the C11-BODIPY (581/591) probe, luminometer kit and western blot analysis (the displayed blots are cropped, and the full-length blots are included in the Supplementary Information file), respectively. **P < 0.01; *P < 0.05.

The mitogen-activated protein kinases (MAPK) signaling pathway is an important signal transduction system in the organism and participates in many physiological and pathological processes^31^. Accumulated literature has reported that MAPK signaling is involved in cell functions from survival to death. In our study, we investigated the involvement of MAPKs in t-BHP-induced cell death. t-BHP treatment significantly increased the protein expression of *p*-JNK (Thr183/Tyr185) and *p*-ERK (Thr202/Tyr204) but had no effect on *p*-p38 mitogen-activated protein kinase (MAPK; Thr180/Tyr182) (Figure S5). Furthermore, SP600125 and U0126 decreased the t-BHP-induced generation of lipid ROS (Fig. 7E), increased the ratio of GSH/GSSG (Fig. 7D), and restored the decreased expression of Gpx4 (Fig. 7B). Interestingly, SP600125 and U0126 also increased the fluorescence ratio of red to green (Fig. 7A), as well as decreased the generation of mitochondrial ROS (Fig. 7A and F) and ATP generation (Fig. 7C).

## Discussion

Cell death is an important biological process that shapes the development of multicellular organisms. Proper regulation of cell death is pivotal for the process of neurodegenerative diseases (NDD). Oxidative damage is a well-demonstrated pathogenic factor in NDD brains. Since the brain is rich in lipids containing polyunsaturated fatty acids, lipid peroxidation is the prominent type of oxidative damage. Lipid peroxidation is believed to be an important event in NDD pathogenesis^32^. Dysfunction of iron, which can augment the production of reactive oxygen species (ROS), is also evident in NDD brains. Interestingly, studies of lipid peroxidation and iron dysregulation revealed a new cell death mechanism, called ferroptosis, a cell death pathway that is genetically, morphologically, and biochemically distinct from other major forms of regulated cell death.

The phenomenon of ferroptosis is characterized by the overwhelming, iron-dependent accumulation of lethal lipid ROS, leading to lipid peroxidation^8,10,33^. Lipid peroxidation is the driving force of cell death in ferroptosis. Cystine/glutamate antiporter (also called system Xc^−^) transports Glu, Cys and cysteine (Cys_2_) into cells and plays important roles in GSH synthesis^34^. GSH depletion can directly activate lipoxygenases and suppress the activity of Gpx4 to trigger lipid peroxidation^35,36^. Gpx4 has been identified as an important regulator of ferroptosis in certain cancer cells^37,38^. Our results showed that the iron chelator DFO could restore t-BHP-induced cell morphology and viability. By contrast, the iron donor FAC further aggravated the cell morphology and decreased t-BHP-induced cell injury, thereby suggesting that cell death induced by t-BHP was iron-dependent. Moreover, the ferroptosis inhibitor Fer-1 could also restore the t-BHP-induced cell morphology and viability, suggesting the involvement of ferroptosis. Furthermore, t-BHP (100 μM) co-treatment with PC12 cells for 1 h increased the generation of lipid ROS, restored the expression of Gpx4, and decreased the ratio of GSH/GSSG. All of these results were reversed by Fer-1 and DFO, but aggravated by FAC, implying the involvement of ferroptosis in t-BHP-induced PC12 death accompanied by alteration of the system Xc^−^.

In studies that investigated the underlying mechanisms in the different paradigms of NDD, oxidative stress was also usually involved in pathways of programmed cell death (PCD) converging at the mitochondria, the key organelle of energy metabolism that secures neuronal functions and survival under physiological conditions^39^. In paradigms of programmed cell death, mitochondrial dysfunction is associated with the clash of the membrane potential and the release of pro-apoptotic proteins such as cytochrome c from the intermembrane space into the cytosol, where they orchestrate the final steps of PCD^40^. Here, mitochondrial dysfunction was also investigated. t-BHP decreased the mitochondrial membrane potential and the production of ATP, as well as increased the generation of mitochondrial ROS. Moreover, the mitochondrial membrane was disrupted, and cytochrome c was released from the mitochondria to the cytoplasm.

There are reports that suggest that ferroptosis was an autophagic cell death mechanism^26^. Autophagic cell death is characterized by the accumulation of large intracellular vesicles and the engagement of the autophagic machinery; however, cell death could be retarded by autophagy inhibitors, such as CQ^41^. The up-regulated LC-3II and down-regulated core autophagic-machinery components marked the occurrence of autophagy^42^. The role of autophagy in t-BHP-induced PC12 death remains elusive. Our results revealed that CQ had no effect on the t-BHP-induced alterations of cell morphology and viability. Furthermore, the number of autophagic vesicles and LC-3 II expression were not increased in response to t-BHP. Therefore, autophagy was not involved in t-BHP-induced cell death under our experimental conditions.

Considering the occurrence of ferroptosis and mitochondrial dysfunction, we further investigated the relationship between these two events. Interestingly, in our study, mitochondrial dysfunction was reversed by Fer-1, the ferroptosis inhibitor. Fer-1 increased the mitochondrial membrane potential and the production of ATP, as well as decreased the generation of mitochondrial ROS.

MAPKs are activated by environmental stressors to regulate multiple cell process, from cell survival to cell death^43,44^. MAPK activation has been implicated in t-BHP-induced cell death^45^. Consistent with a previous study in HepG2 cells, JNK1/2 and ERK1/2 were activated by t-BHP. JNK1/2 and p38 MAPK, but not ERK1/2, play pivotal roles in erastin-induced ferroptosis in leukemia cells^33^. U0126, an ERK inhibitor, could reverse erastin-induced ferroptosis in HT1080 cells^10^. In the present study, JNK1/2 and ERK1/2 were activated by t-BHP (100 μM) within 1 h in PC12 cells. SP600125 (JNK1/2 inhibitor) and U0126 (ERK1/2 inhibitor) increased the cell viability induced by t-BHP. Moreover, SP600125 and U0126 inhibited t-BHP-induced lipid ROS generation, increased the ratio of GSH/GSSG, and increased the expression of Gpx4. Furthermore, SP600125 and U0126 increased the mitochondrial membrane potential, restored ATP production, and decreased the generation of mitochondrial ROS.

t-BHP^46^ is a membrane-permeable oxidant compound that could induce different routes of cell death^47^. Low concentrations of t-BHP (2.5 μM) induced the phosphorylation of ERK1/2, JNK1/2 and p38 MAPK and activated apoptotic cascades in PC12 cells^45^. High concentrations of t-BHP (500 μM) induced the mitochondria-mediated apoptosis pathway in RAW264.7 cells^48^. t-BHP co-treatment at 100 μM induced the mitochondrial-mediated apoptosis pathway in HepG2 cells^49^. t-BHP co-treatment of PC12 cells at 75–150 μM indicated no cleavage of the apoptosis effector enzyme caspase 3 in retinal pigment epithelial cells but did induce RIPK3-independent necrosis^50^. t-BHP co-treatment at 300 μM induced an ~ 80% cell viability decrease in PC12 cells^51^. Cell viability decreased to 40% when co-treated with 300 μM for 3 h in PC12 cells^37^. Here, in our study, t-BHP induced ferroptosis, and this mechanism of cell death was coupled with mitochondrial dysfunction during co-treatment of PC12 cells with 100 μM t-BHP for 1 h.

**Figure 8:**
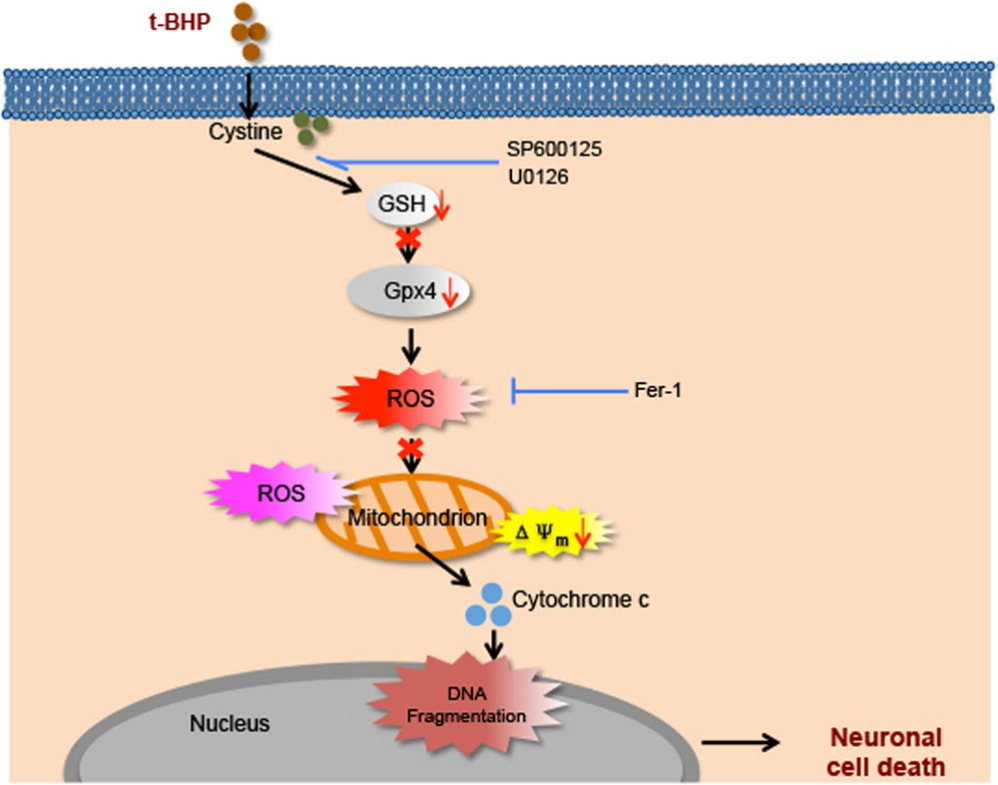
t-BHP-induced cell death routine in PC12 cells. In PC12 cells treated with 100 μM t-BHP for 1 h, the membrane of PC12 cells underwent lipid peroxidation, accompanied by GSH depletion, reduced Gpx4 expression, and increased lipid ROS. These lethal ROS further damaged the mitochondria, increased mitochondrial ROS generation, decreased the mitochondrial membrane potential, and increased the release of cytochrome c to the cytoplasm, ultimately leading to cell death. Moreover, in this death process, SP600125 and U0126 acted upstream of these events, and Fer-1 acted upstream of mitochondrial dysfunction.

This study provided comprehensive evidence supporting that t-BHP-induced PC12 cell death involves ferroptosis and mitochondrial dysfunction. Fer-1 reversed mitochondrial dysfunction, and JNK1/2 and ERK1/2 were important regulators of this cell model and reversed the cell death induced by t-BHP (Fig. 8). Considering the close link between oxidative stress and NDD, this study might provide new insight into the prevention of cell loss in NDD.

## Materials and Methods

### Materials

*tert*-Butylhydroperoxide (t-BHP, B106035) was purchased from Aladdin (Shanghai, China). The Gpx4 (ab219592, 1:1000) antibody was purchased from Abcam (CA, USA). The caspase-3 (9665s, 1:1000), cleaved-caspase-3 (9661s, 1:1000), caspase-7 (9492s, 1:1000), cleaved caspase-7 (8438s, 1:1000), caspase-9 (9502s, 1:1000), cleaved caspase-9 (7237s, 1:1000), XIAP (14334s, 1:1000), c-IAP1 (7065s, 1:1000), c-IAP2 (3130s, 1:1000), LC3B (3868s, 1:1000), *p*-MLKL (Ser 358) (91689s, 1:1000), MLKL (14993s, 1:1000), Bid (2002s, 1:1000), Bcl-2 (2870s, 1:1000), JNK (9258s, 1:1000), *p*-JNK (Thr183/Tyr185) (4668s, 1:1000), ERK (4695s, 1:1000), *p*-ERK (Thr202/Tyr204) (4370s, 1:1000), p38 (8690s, 1:1000), *p*-p38 MAPK (Thr180/Tyr182) (4511s, 1:1000), cytochrome *c* (4280s, 1:1000), COX IV (4850s, 1:1000) and GAPDH (5174s, 1:1000) antibodies were purchased from Cell Signaling Technology (MA, USA). RIPK1 (3279–100, 1:500) and RIPK3 (PAB2747, 1:500) were purchased from Abnova (Taiwan, China). C11-BODIPY (581/591) (D3861), 2′,7′-dichlorodihydrofluorescein diacetate (DCFH_2_-DA) (D399), MitoSOX^TM^ Red Mitochondrial Superoxide Indicator (M36008), the SuperSignal West Femto Chemi-luminescence Substrate, and the Mitochondria Isolation Kit (89801) were purchased from Thermo Fisher Scientific (MA, USA). The Caspase-Glo 3/7 Assay Kit (G8090) and GSH/GSSG Assay Kit (V6611) were purchased from Promega (Beijing, China). The Annexin V-FITC/PI apoptotic assay kits (556547) were purchased from BD (NJ, USA). Ferrostatin-1 (Fer-1) (SML0583), deferoxamine (DFO) (D9533), chloroquine (CQ) (C7874), necrostatin-1(Nec-1) (N9037), and monodansylcadaverine (MDC) (D4008) were purchased from Sigma (MO, USA). Necrostatin-1s (Nec-1s) (2263-1) was purchased from BioVision (SF, USA). 2, 3, 6-2′, 5′-Pentachlorinated biphenyl (PCB-95) (RPC-130AS) was purchased from Ultra Scientific (RI, USA). Ammonium ferric citrate (FAC) (A100170) and Z-VAD-FMK (C1202) were purchased from Aladdin (Shanghai, China). N-Acetyl-_L_-cysteine (NAC) (S0077), the JC-1 staining kit (C2006) and the lactate dehydrogenase (LDH) assay kit (C0016) were purchased from Beyotime Biotechnology (Haimen, China).

### Cell culture

PC12 cells obtained from the American Type Culture Collection (ATCC; USA), were cultured in complete DMEM/F-12K medium containing 5% fetal bovine serum and 10% horse serum. Cells were incubated at 37 °C in a humidified atmosphere of 5% CO_2_ in air.

### Cell viability assay

Cell viability was determined by the MTT assay. PC12 cells (5 × 10^4^/well) were seeded in a 96-well plate overnight before treatment with t-BHP (50 μM, 100 μM, 200 μM and 400 μM) for 0.5 h, 1 h, 2 h and 4 h with or without other co-treatment. Subsequently, 20 μl of the MTT solution (5 mg/ml) was added to each well and incubated at 37 °C for 4 h. The supernatant was removed, and the insoluble formazan product was dissolved in 100 μl of DMSO. The absorbance of each culture well was measured with a microplate reader (Molecular Devices, USA) at a wavelength of 570 nm.

### Dynamic imaging

PC12 cells (5 × 10^4^/well) were seeded in 96-well black-walled multiwall plates. When cells grew to confluence, they were co-treated with 5 μM PI and 5 μM Calcein-AM for 20 min, and then treated with Hoechst 33342 for 5 min. After this the cells were treated with different concentration of t-BHP (50 μM, 100 μM, 200 μM and 400 μM). Upon addition of the t-BHP, the fluorescence was detected in EVOS FL auto 2 for as long as 4 h.

PC12 cells (5 × 10^4^/well) were seeded in 96-well plates. When cells grew to confluence, upon addition of t-BHP (50 μM, 100 μM, 200 μM and 400 μM), the plate was detected in EVOS FL auto2 for as long as 4 h to observe the cell condition.

### Electron microscopy observation for cell morphology changes

PC12 cells were seeded in 10-cm dishes. The following day, cells were treated with or without t-BHP (100 μM) for 1 h. Cells were collected in PBS, gently spun down, fixed in 2% paraformaldehyde, 2.5% glutaraldehyde in 0.1 M sodium cacodylate buffer before washing in 0.1 M sodium cacodylate buffer followed by post-fixing in 1% osmium tetroxide in 0.1 M sodium cacodylate buffer. Following fixation, cells were dehydrated through a graded series of alcohols and embedded in Spurrs Resin. Ultrathin sections were cut with a diamond knife using a Leica Ultracut S ultra-microtome and stained with both methanolic uranyl acetate and lead citrate before viewing in a LEO 906 transmission electron microscope operated at 60 kV.

### Measurement of ROS generation

Approximately 1.0 × 10^6^ cells/well were seeded in 6-well plates overnight followed by treatment with inhibitors or activators for 24 h and subsequent treatment with t-BHP (100 μM) for 1 h. The cells were incubated with DCFH_2_-DA or C11-BODIPY (581/591) or MitoSOX^TM^ Red Mitochondrial Superoxide Indicator in the dark at 37 °C for 30 min^10^. Cells were washed twice with PBS and detached with trypsin/EDTA. The cellular fluorescence was analyzed by flow cytometry (Becton Dickinson FACS Canto ^TM^, USA). A total of 10000 events in the gate were acquired for each sample from three different experiments. Then, the data were analyzed by FlowJo vx0.7.

### MDC staining

After treatment with t-BHP (100 μM) for 1 h, the autophagic vacuoles of PC12 cells were detected with MDC staining. PC12 cells (5 × 10^4^/well) were incubated with MDC (50 μM) in a 96-well plate with serum-free medium at 37 °C for 30 min in the dark. After incubation, cells were washed four times with cold PBS, and fluorescent micrographs were obtained with a Cell Analyzer 2000^52^.

### Determination of Caspase-3 and Caspase-7 activity

PC12 cells (5 × 10^4^/well) were cultured in 96-well white-walled multiwall luminometer plates. After treatment with t-BHP (100 μM) for 1 h, the activity of caspase-3 and caspase-7 was detected with a luminometer kit following the manufacturer’s instructions.

### JC-1 staining

PC12 cells (5 × 10^4^/well) were cultured in 96-well black-walled multiwall plates. After treatment with t-BHP (100 μM) for 1 h, the mitochondrial membrane potential was detected by JC-1 staining with a commercial kit according to the manufacturer’s instructions. Images were analyzed by Image J.

### Determination of GSH/GSSG activity

PC12 cells (5 × 10^4^/well) were cultured in 96-well white-walled multiwall luminometer plates. After treatment with t-BHP (100 μM) for 1 h, the GSH/GSSG ratio was detected with a luminometer kit following the manufacturer’s instructions.

### Annexin V-FITC/PI staining

To distinguish between dead cells and apoptotic cells, 10^6^ cells/mL of each sample were co-stained with 5 μg/mL propidium iodide (PI) and fluorescein isothiocyanate (FITC)-conjugated annexin V. Cell counts were acquired with a FACS Calibur flow cytometer, and cell fractions in each quadrant were analyzed using Cell Quest Pro software. Cells in the lower right quadrant represented early apoptosis, while cells in the upper right quadrant represented late apoptosis. Dead cells were found in the upper left quadrant. Fluorescence compensation on the flow cytometer was adjusted to minimize overlap of the FITC and PI signals. A total of 10000 events in the gate were acquired for each sample from three different experiments.

### Western Blot analysis

PC12 cells were collected, and the total protein was extracted after treatment with t-BHP or inhibitors. The protein concentrations were detected by the BCA protein assay according to the manufacturer’s instructions. Thirty micrograms of cellular protein from each group was electro-blotted onto PVDF membrane following separation on 8% SDS-PAGE. The immuno-blot was incubated with the blocking solution (5% skim milk) at room temperature for 1 h, followed by incubation with a primary antibody overnight at 4 °C. After washing with Tween 20/Tris-buffered saline (TBST), the immune-blot was incubated with the respective secondary antibodies (1:10,000) for 2 h at room temperature. Blots were developed by the SuperSignal West Femto Chemi-luminescence Substrate and quantified by Image J.

Mitochondrial and cytoplasmic proteins were isolated with a commercial kit following the manufacturer’s instructions.

### Statistical analysis

The data were expressed as means ± SD and analyzed with one-way ANOVA by SPSS (17.0) for statistical significance using one-way analysis of variance^53^. *P* < 0.05 was considered statistically significant.

## Electronic supplementary material

Supplementary information file

## References

[1] Chan FK, Luz NF, Moriwaki K (2015). Programmed necrosis in the cross talk of cell death and inflammation. Annu. rev. immunol..

[2] Conrad, M., Angeli, J. P., Vandenabeele, P. & Stockwell B. R. Regulated necrosis: Disease relevance and therapeutic opportunities. *Nat. rev. drug discov*. 2016 10.1038/nrd.2015.6 PMC6531857 26775689

[3] Vanden-Berghe T, Linkermann A, Jouan-Lanhouet S, Walczak H, Vandenabeele P (2014). Regulated necrosis: The expanding network of non-apoptotic cell death pathways. Nat. rev. Mol. cell bio..

[4] Brinkmann V, Zychlinsky A (2012). Neutrophil extracellular traps: Is immunity the second function of chromatin?. J. cell biol..

[5] Green DR, Levine B (2014). To be or not to be? How selective autophagy and cell death govern cell fate. Cell..

[6] Galluzzi L, Pietrocola F, Levine B, Kroemer G (2014). Metabolic control of autophagy. Cell..

[7] Neitemeier S (2017). Bid links ferroptosis to mitochondrial cell death pathways. Redox bio..

[8] Yang WS, Stockwell BR (2016). Ferroptosis: Death by lipid peroxidation. Trends cell biol..

[9] Speer RE (2013). Hypoxia-inducible factor prolyl hydroxylases as targets for neuroprotection by “antioxidant” metal chelators: From ferroptosis to stroke. Free radical bio. med..

[10] Dixon SJ (2012). Ferroptosis: An iron-dependent form of nonapoptotic cell death. Cell..

[11] Greene LA, Tischler AS (1976). Establishment of a noradrenergic clonal line of rat adrenal pheochromocytoma cells which respond to nerve growth factor. Proc. Natl. Acad. Sci. USA.

[12] Shah M (2014). The high-affinity d2/d3 agonist d512 protects pc12 cells from 6-ohda-induced apoptotic cell death and rescues dopaminergic neurons in the mptp mouse model of parkinson’s disease. J. neurochem..

[13] Renaud J, Bournival J, Zottig X, Martinoli MG (2014). Resveratrol protects daergic pc12 cells from high glucose-induced oxidative stress and apoptosis: Effect on p53 and grp75 localization. Neurotox. res..

[14] Pislar AH, Zidar N, Kikelj D, Kos J (2014). Cathepsin x promotes 6-hydroxydopamine-induced apoptosis of pc12 and sh-sy5y cells. Neuropharmacology..

[15] Li J (2014). Paeoniflorin attenuates abeta25-35-induced neurotoxicity in pc12 cells by preventing mitochondrial dysfunction. Folia neuropathol..

[16] Hu X (2014). Differential protein profile of pc12 cells exposed to proteasomal inhibitor lactacystin. Neurosci. lett..

[17] Han X (2014). Antioxidant action of 7,8-dihydroxyflavone protects pc12 cells against 6-hydroxydopamine-induced cytotoxicity. Neurochem. int..

[18] Pasban-Aliabadi H (2013). Inhibition of 6-hydroxydopamine-induced pc12 cell apoptosis by olive (olea europaea l.) leaf extract is performed by its main component oleuropein. Rejuv. res..

[19] Sutherland MW, Nelson J, Harrison G, Forman HJ (1985). Effects of t-butyl hydroperoxide on nadph, glutathione, and the respiratory burst of rat alveolar macrophages. Arch. biochem. biophys..

[20] Dise CA, Goodman DB (1986). T-butyl hydroperoxide alters fatty acid incorporation into erythrocyte membrane phospholipid. Biochim. biophys. acta..

[21] Rice-Evans C, Baysal E, Pashby DP, Hochstein P (1985). T-butyl hydroperoxide-induced perturbations of human erythrocytes as a model for oxidant stress. Biochim. biophys. acta..

[22] Sun X (2016). Activation of the p62-keap1-nrf2 pathway protects against ferroptosis in hepatocellular carcinoma cells. Hepatology..

[23] Yang WS (2014). Regulation of ferroptotic cancer cell death by gpx4. Cell..

[24] Barber AJ, Gardner TW, Abcouwer SF (2011). The significance of vascular and neural apoptosis to the pathology of diabetic retinopathy. Invest. ophth. vis. sci..

[25] Levine B, Yuan J (2005). Autophagy in cell death: An innocent convict?. J. clin. invest..

[26] Hou W (2016). Autophagy promotes ferroptosis by degradation of ferritin. Autophagy..

[27] Strzyz P (2016). Cell death: Molecular insights into execution of necroptosis. Nat. rev. mol. cell bio..

[28] Zhang DW (2009). Rip3, an energy metabolism regulator that switches tnf-induced cell death from apoptosis to necrosis. Science..

[29] He S (2009). Receptor interacting protein kinase-3 determines cellular necrotic response to tnf-alpha. Cell..

[30] Sun L (2012). Mixed lineage kinase domain-like protein mediates necrosis signaling downstream of rip3 kinase. Cell..

[31] Yao B (2017). Mapk signaling pathways in eye wounds: Multifunction and cooperation. Exp. cell res..

[32] Hambright WS, Fonseca RS, Chen L, Na R, Ran Q (2017). Ablation of ferroptosis regulator glutathione peroxidase 4 in forebrain neurons promotes cognitive impairment and neurodegeneration. Redox bio..

[33] Xie Y (2016). Ferroptosis: Process and function. Cell death differ..

[34] Robert SM, Ogunrinu-Babarinde T, Holt KT, Sontheimer H (2014). Role of glutamate transporters in redox homeostasis of the brain. Neurochem. int..

[35] Loscalzo J (2008). Membrane redox state and apoptosis: Death by peroxide. Cell metab..

[36] Seiler A (2008). Glutathione peroxidase 4 senses and translates oxidative stress into 12/15-lipoxygenase dependent- and aif-mediated cell death. Cell metab..

[37] Maiwulanjiang M (2013). Song bu li decoction, a traditional uyghur medicine, protects cell death by regulation of oxidative stress and differentiation in cultured pc12 cells. Evid-based compl. alt: eCAM..

[38] Fonck C, Baudry M (2001). Toxic effects of mpp(+) and mptp in pc12 cells independent of reactive oxygen species formation. Brain res..

[39] Galluzzi L, Kepp O, Kroemer G (2012). Mitochondria: Master regulators of danger signalling. Nat. rev. mol. cell bio..

[40] Flippo KH, Strack S (2017). Mitochondrial dynamics in neuronal injury, development and plasticity. J. Cell Sci..

[41] Green, D.R. & Llambi, F. Cell death signaling. *CSH. Perspect. biol*. 7 (2015). 10.1101/cshperspect.a006080 PMC4665079 26626938

[42] Galluzzi L (2015). Autophagy in malignant transformation and cancer progression. EMBO J..

[43] Wada T, Penninger JM (2004). Mitogen-activated protein kinases in apoptosis regulation. Oncogene..

[44] Karin M (1998). Mitogen-activated protein kinase cascades as regulators of stress responses. Ann. NY. acad. sci..

[45] Li YB (2011). Protective, antioxidative and antiapoptotic effects of 2-methoxy-6-acetyl-7-methyljuglone from polygonum cuspidatum in pc12 cells. Planta med..

[46] Martin C (2001). Tert-butyl hydroperoxide-induced lipid signaling in hepatocytes: Involvement of glutathione and free radicals. Biochem. Pharmacol..

[47] Zhao K, Luo G, Giannelli S, Szeto HH (2005). Mitochondria-targeted peptide prevents mitochondrial depolarization and apoptosis induced by tert-butyl hydroperoxide in neuronal cell lines. Biochem. Pharmacol..

[48] Han L (2012). Inhibitory effects of trolox-encapsulated chitosan nanoparticles on tert-butylhydroperoxide inducedraw264.7 apoptosis. Biomaterials..

[49] Kim SC, Lee JR, Park SJ (2014). Role of 6-shogaol in tert -butyl hydroperoxide-induced apoptosis of hepg2 cells. Pharmacology..

[50] Hanus J (2013). Induction of necrotic cell death by oxidative stress in retinal pigment epithelial cells. Cell death dis..

[51] Zhao Y, Dou J, Wu T, Aisa HA (2013). Investigating the antioxidant and acetylcholinesterase inhibition activities of gossypium herbaceam. Molecules..

[52] Su Z (2016). Overexpression of rbm5 induces autophagy in human lung adenocarcinoma cells. World j. surg. oncol..

[53] Moyanova SG, Dijkhuizen RM (2014). Present status and future challenges of electroencephalography- and magnetic resonance imaging-based monitoring in preclinical models of focal cerebral ischemia. Brain res. bull..

