# Supplementary material for: Induction of ferroptosis and mitochondrial dysfunction by oxidative stress in PC12 cells
Source: Sci Rep. 2018 Jan 12;8:574. doi: 10.1038/s41598-017-18935-1 (PMC5766540; doi:10.1038/s41598-017-18935-1)
Supplement: Supplementary file 1 — Supplementary information file [file 41598_2017_18935_MOESM1_ESM.doc]

**Induction of ferroptosis and mitochondrial dysfunction by oxidative stress in PC12 cells**

Chuanhong Wu^1^, Wenwen Zhao^1^, Jie Yu^1^, Shaojing Li^2^, Ligen Lin^1^, Xiuping Chen^1,*^

^1^State Key Laboratory of Quality Research in Chinese Medicine, Institute of Chinese Medical Sciences, University of Macau, Macao, China

^2^Institute of Chinese Materia Medica, China Academy of Chinese Medical Sciences, Beijing 100700, China

Running title: Oxidative stress induced PC12 death

*Send correspondence to: Dr. Xiuping Chen

Address: Institute of Chinese Medical Sciences, University of Macau, Avenida da Universidade, Taipa, Macau, China

Correspondence to [email address]: xpchen@umac.mo

Tel: +853-88224679

Fax: +853-28841358

1. The full-length blots of Figure 4.

Figure 3 D

Gpx4: 0 min, 30 min, 60 min, 120 min


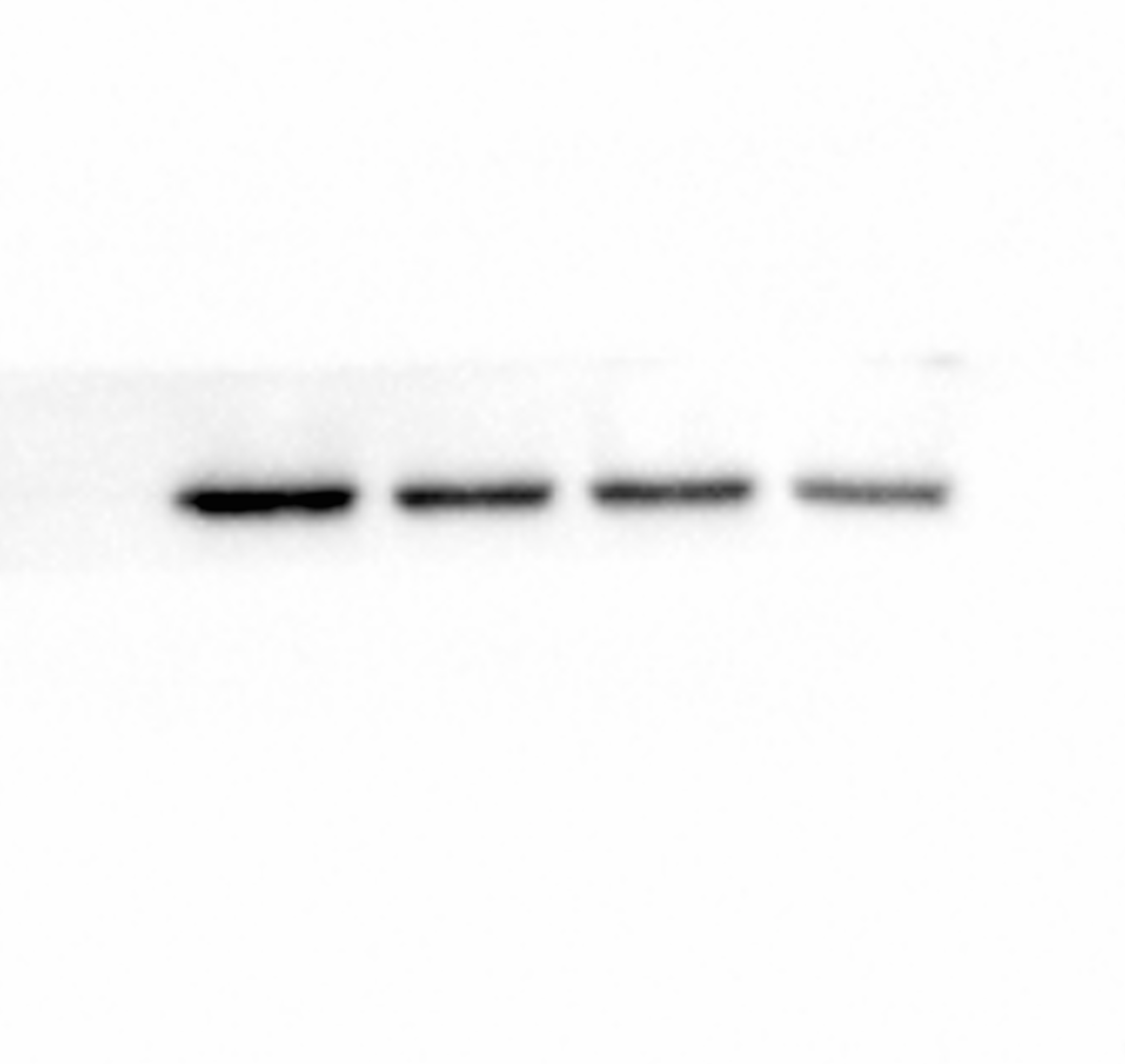


GAPDH: 0 min, 30 min, 60 min, 120 min


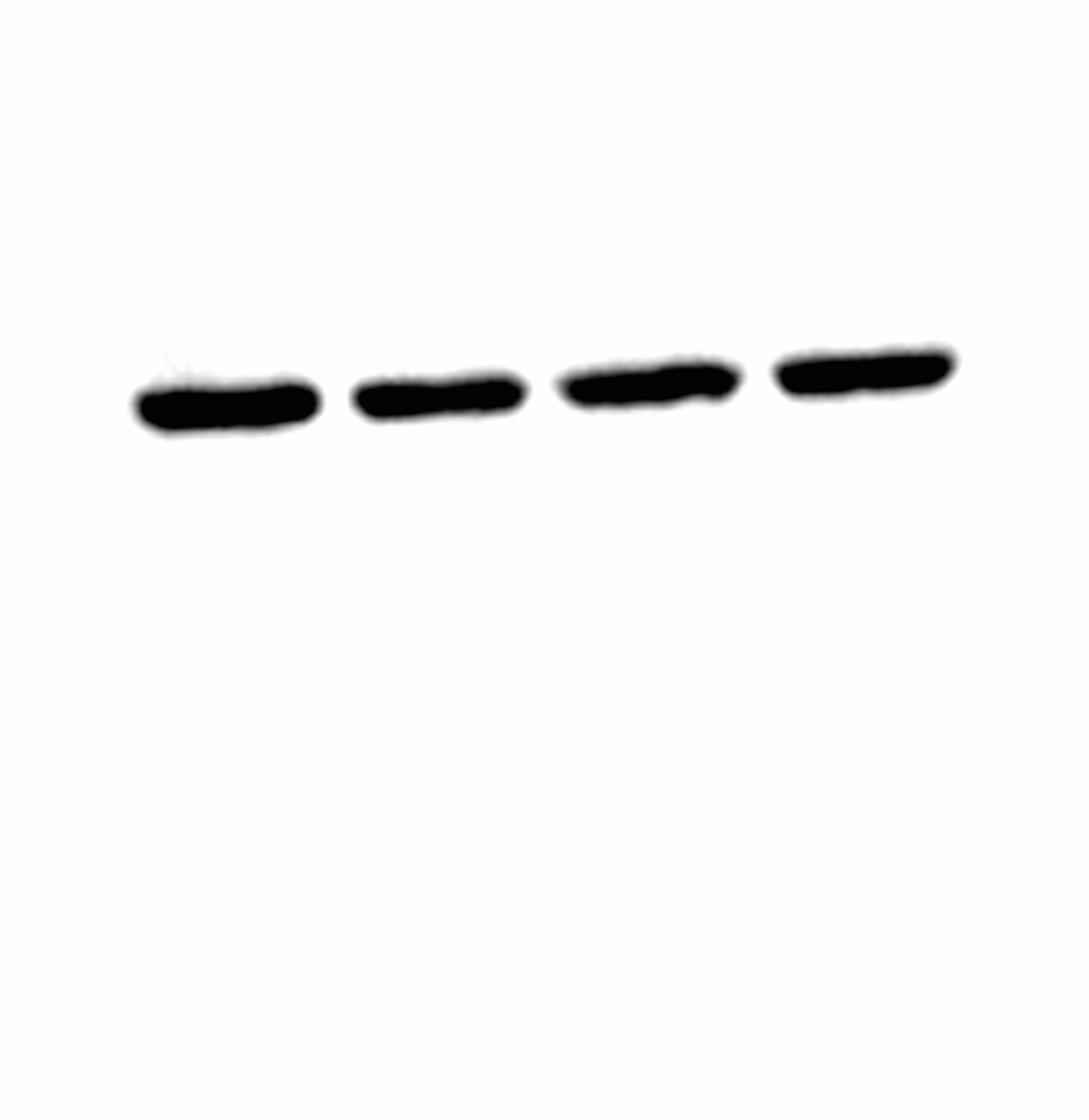


Figure 3 E

Gpx4: Control, t-BHP, Fer-1+t-BHP


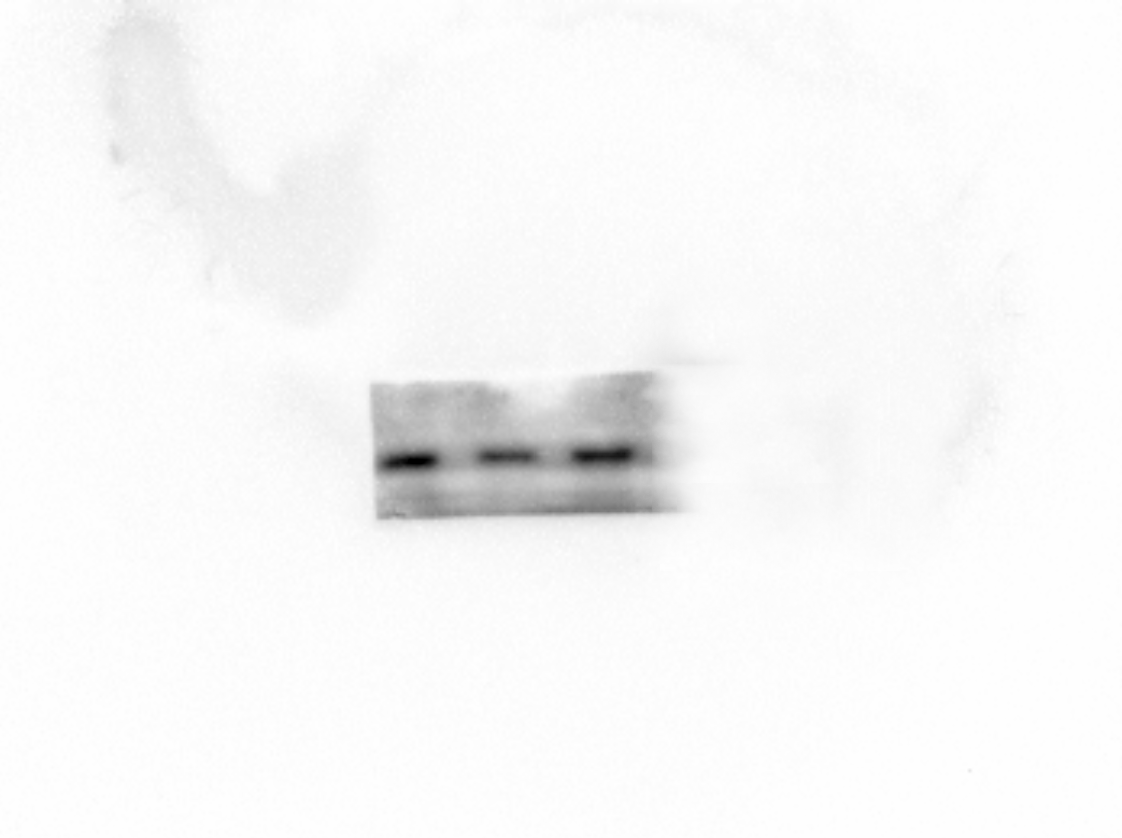


GAPDH: Control, t-BHP, Fer-1+t-BHP


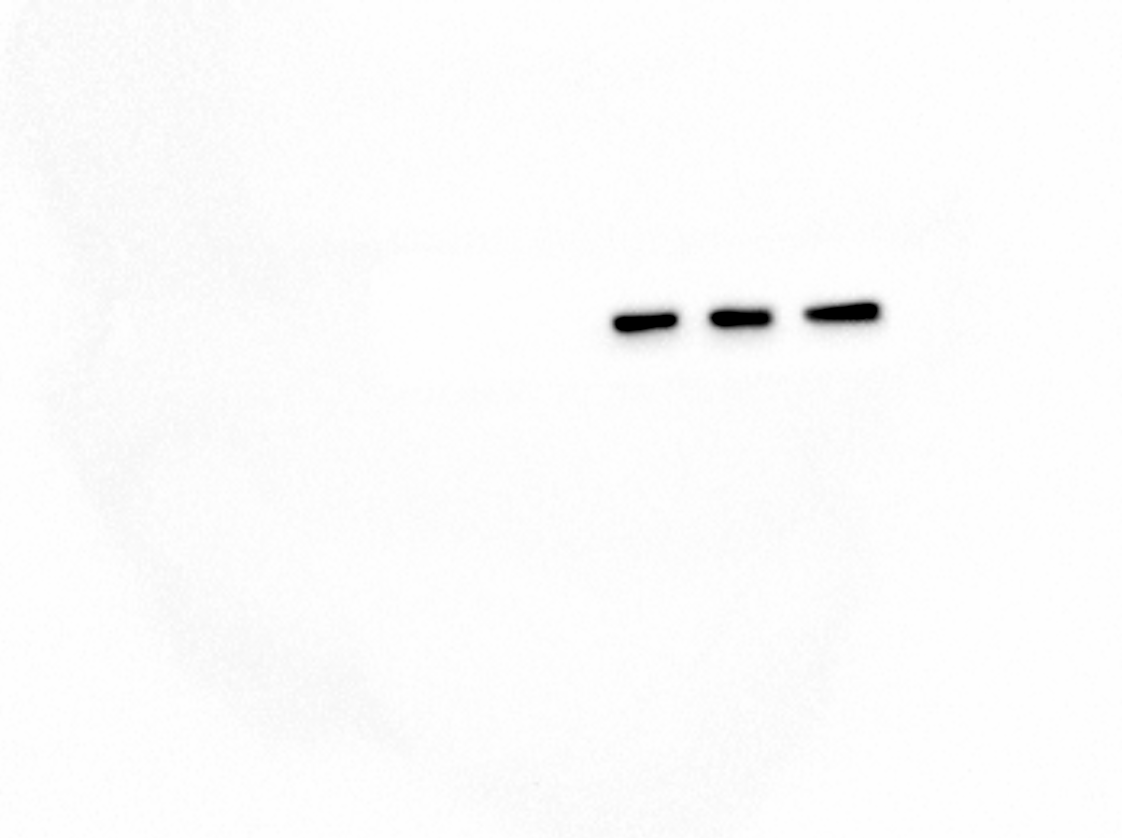


Figure 3 F

Gpx4: Control, t-BHP, FAC+t-BHP, DFO+t-BHP


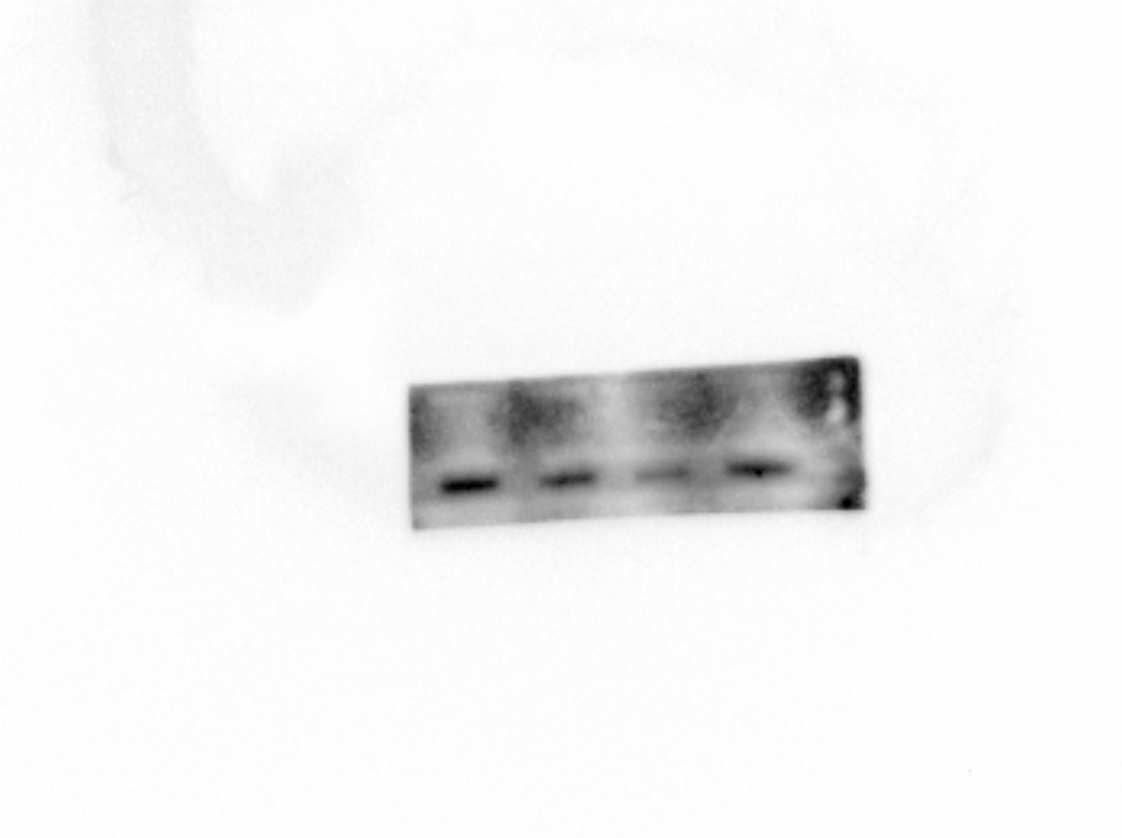


GAPDH: Control, t-BHP, FAC+t-BHP, DFO+t-BHP


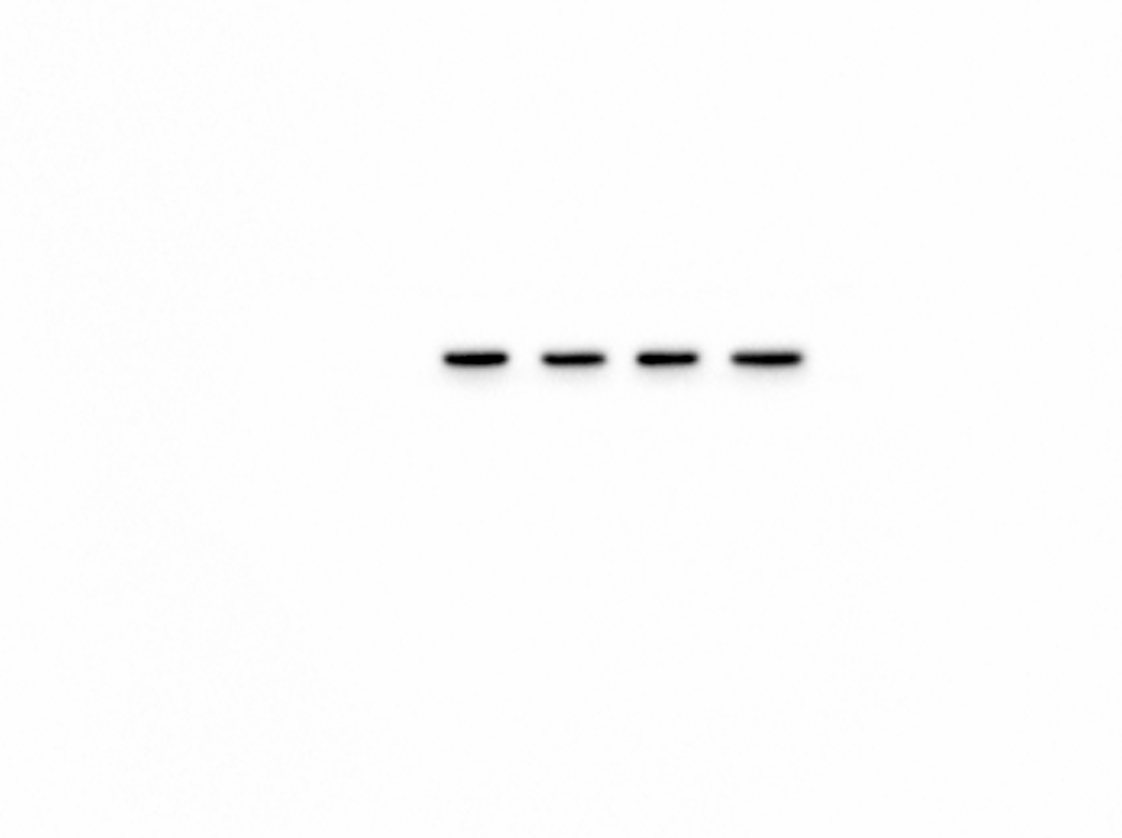


The full-length blots of Figure 4.

Figure 4 C

Cytochrome c: Control, t-BHP (Cytoplasma); Control, t-BHP (Mitochondrion).


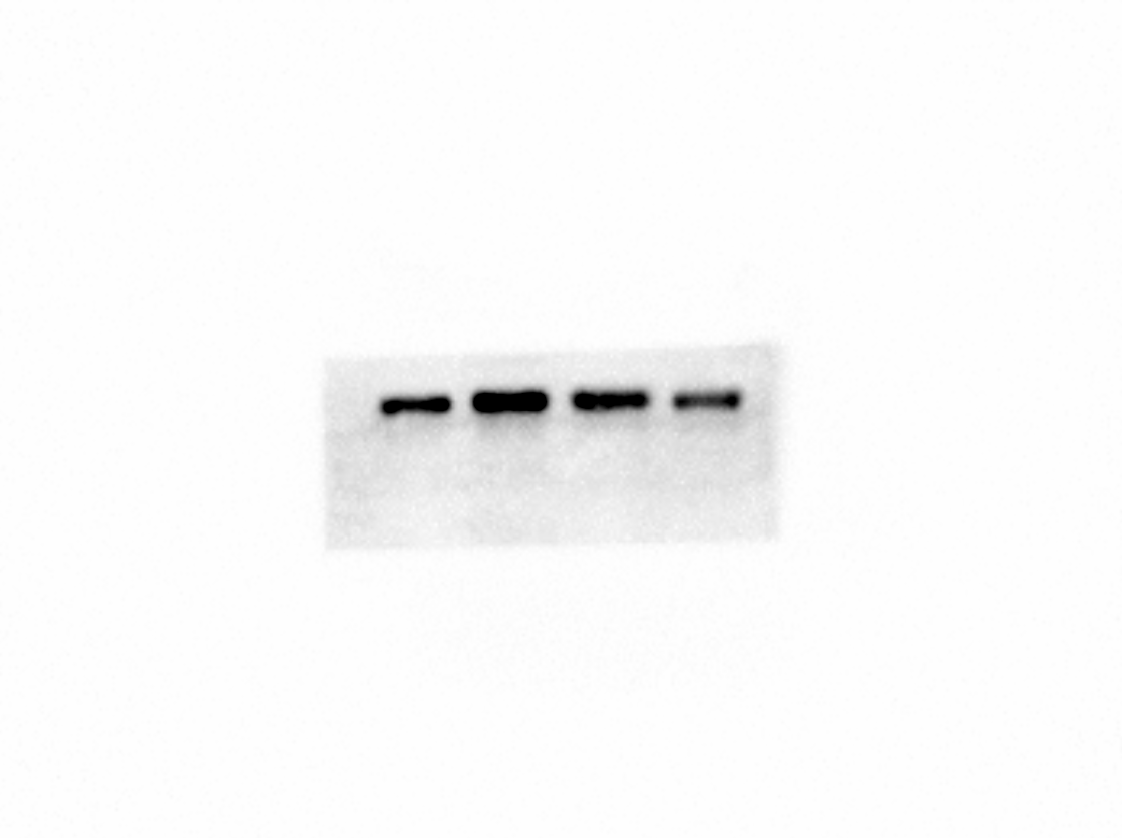


COXIV: Control, t-BHP


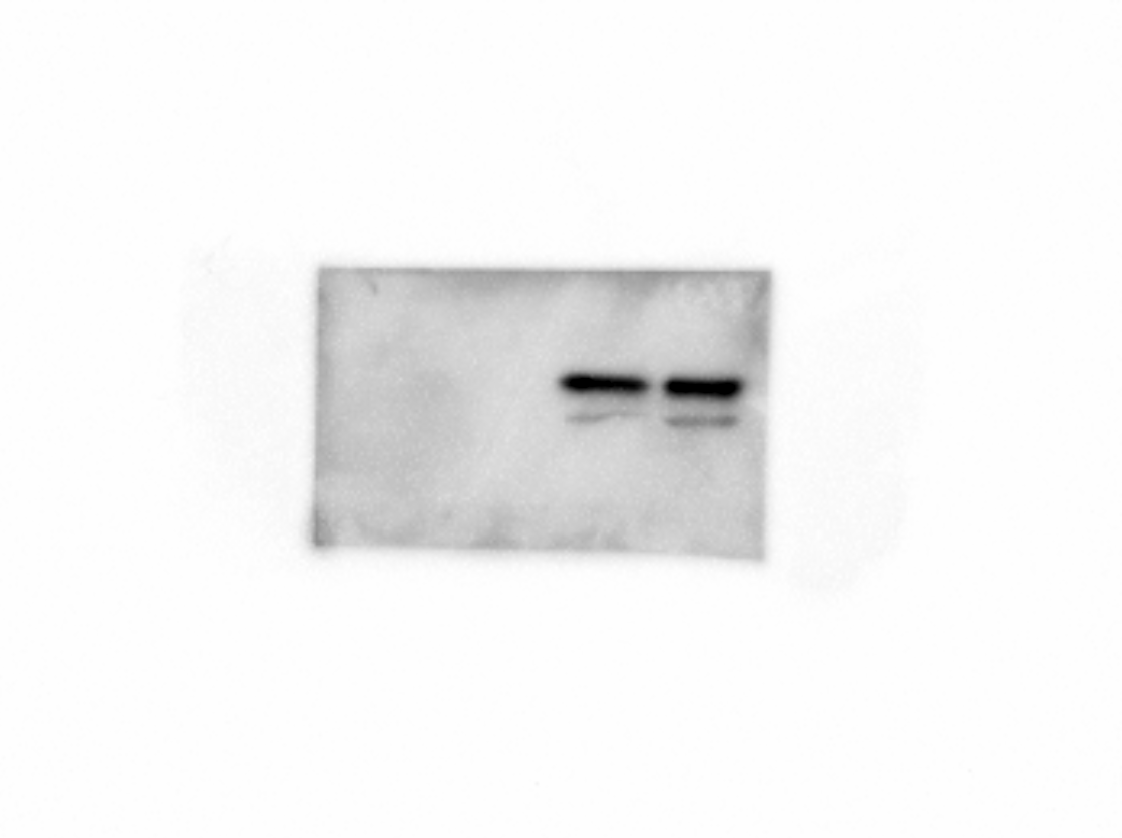


GAPDH: Control, t-BHP


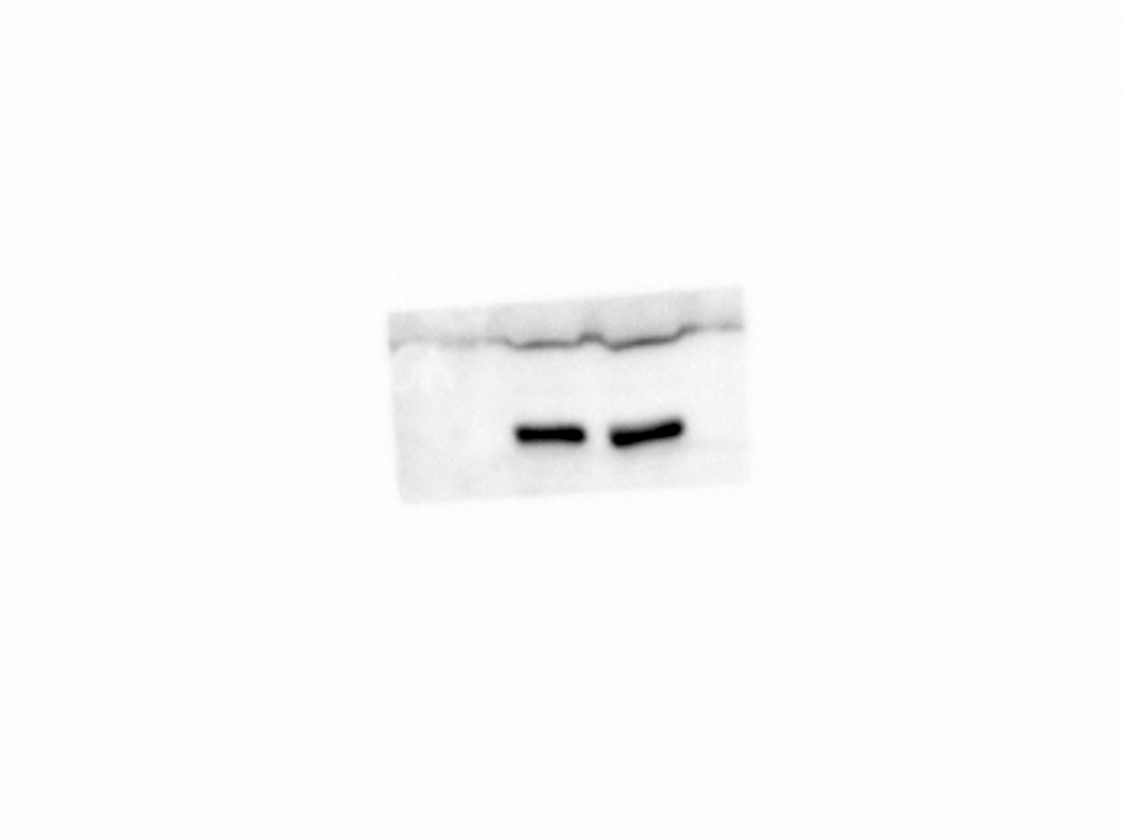


Figure 4 D

Bid: Control, t-BHP


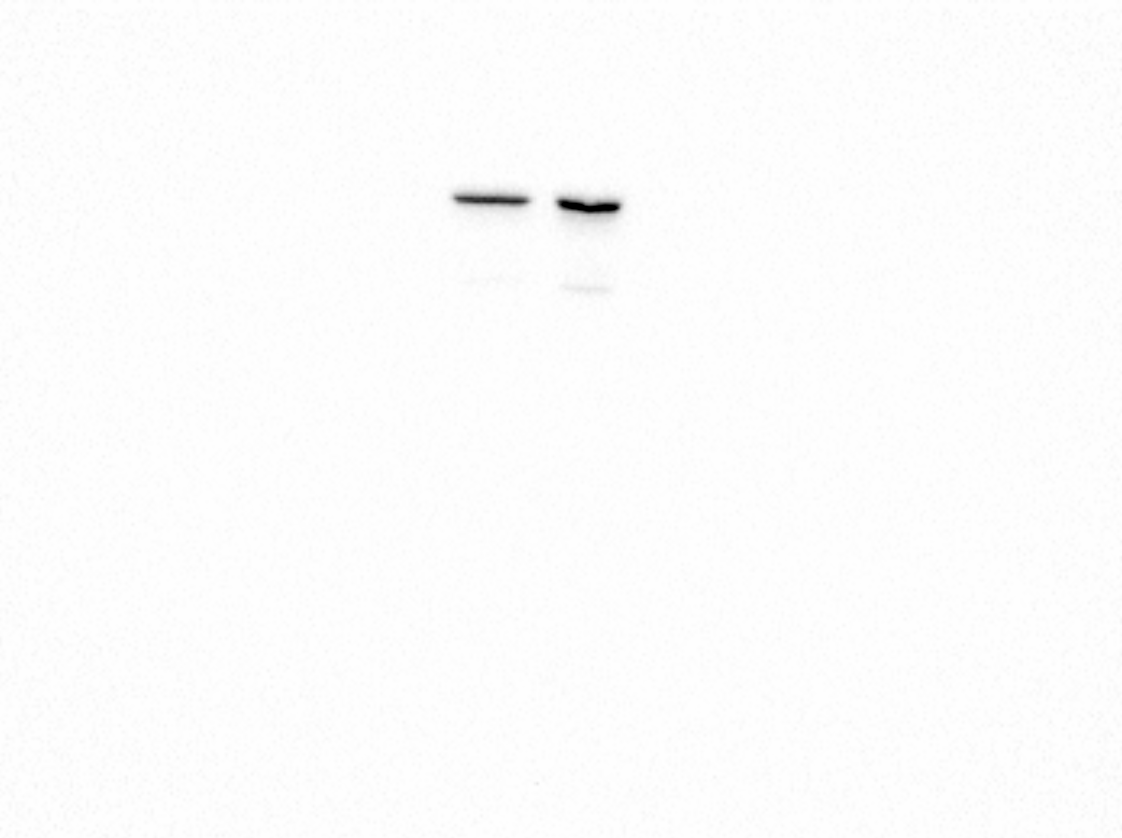


Bcl-2: Control, t-BHP


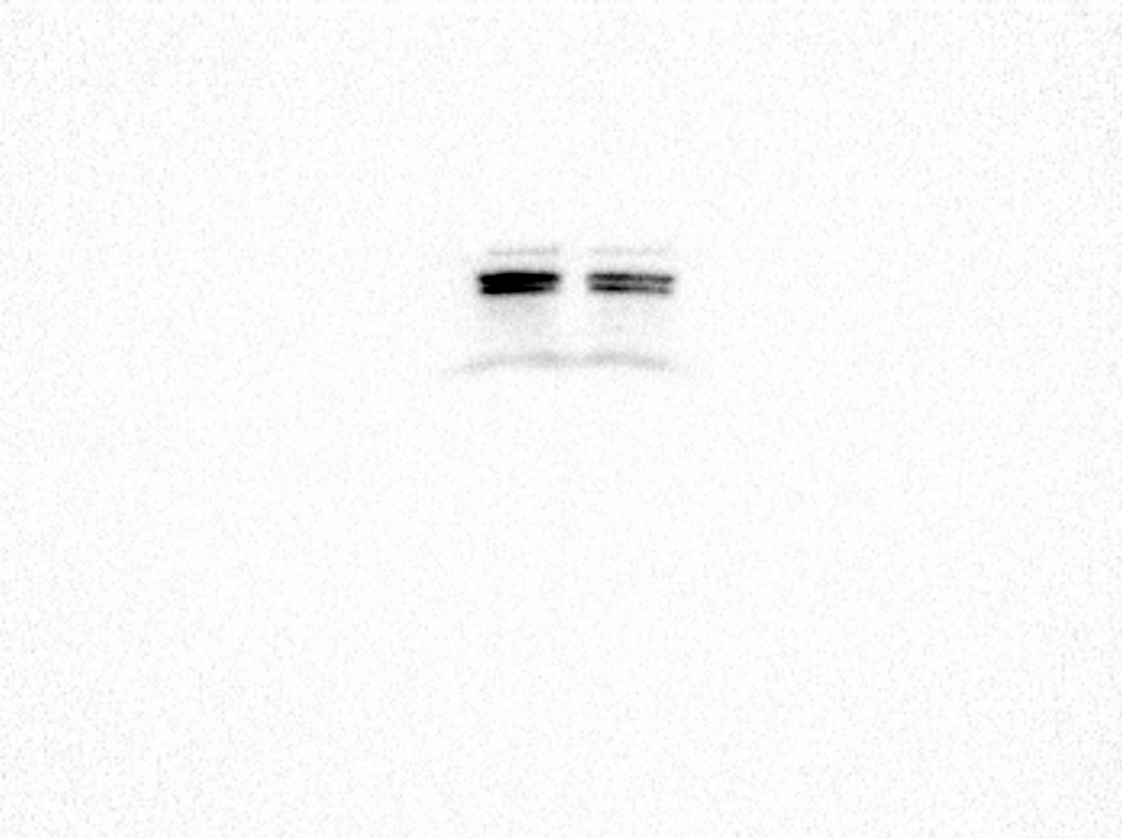


GAPDH: Control, t-BHP


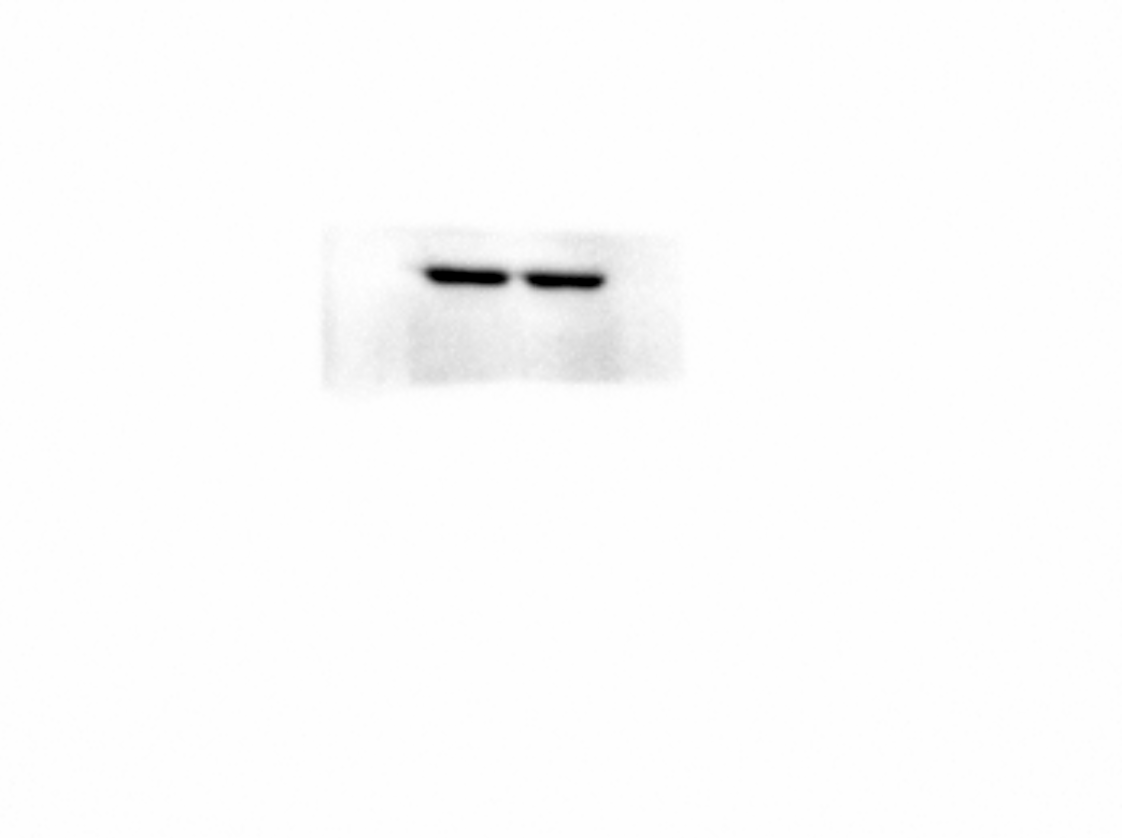


The full-length blots of Figure 5.

Figure 5 B

LC-3I/II: Control, t-BHP (0 min); Control, t-BHP (15 min); Control, t-BHP (30 min); Control, t-BHP (45 min); Control, t-BHP (60 min); Control, t-BHP (120 min).


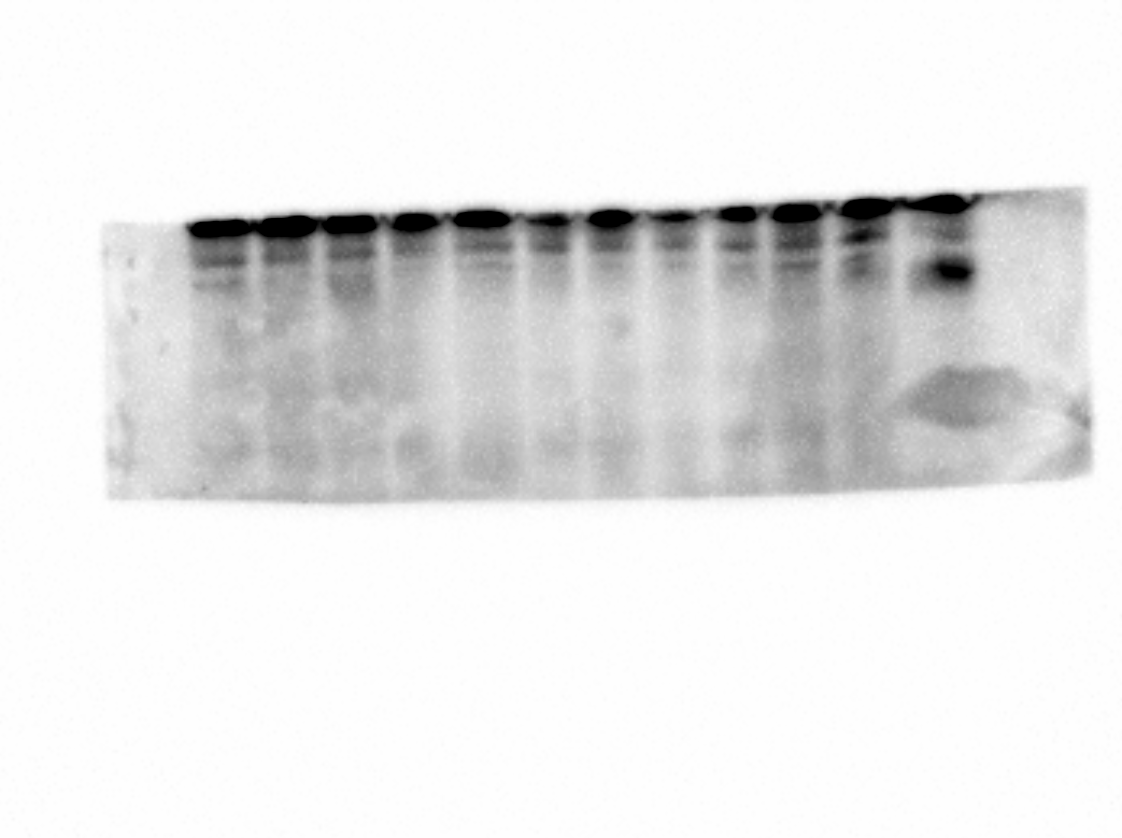


GAPDH: Control, t-BHP (0 min); Control, t-BHP (15 min); Control, t-BHP (30 min); Control, t-BHP (45 min); Control, t-BHP (60 min); Control, t-BHP (120 min).


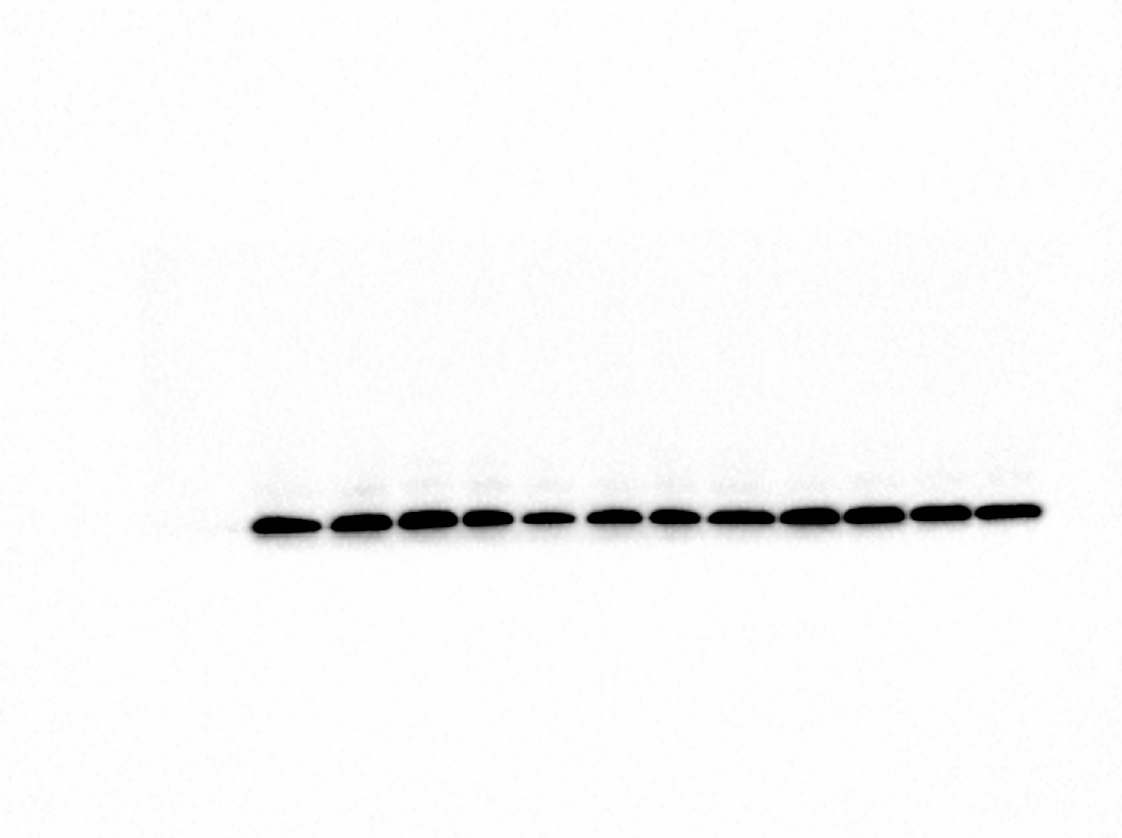


Figure 5 C

RIPK1: Control, t-BHP, PCB-95.


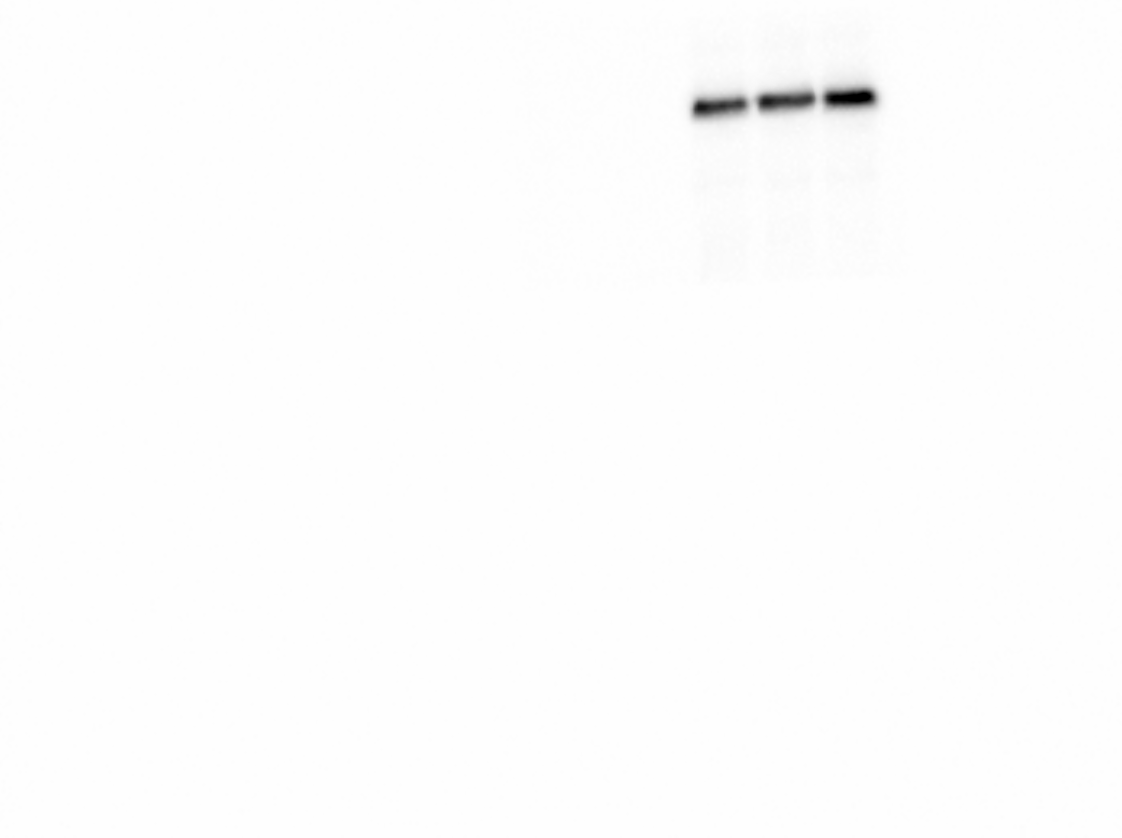


RIPK3: Control, t-BHP, PCB-95.


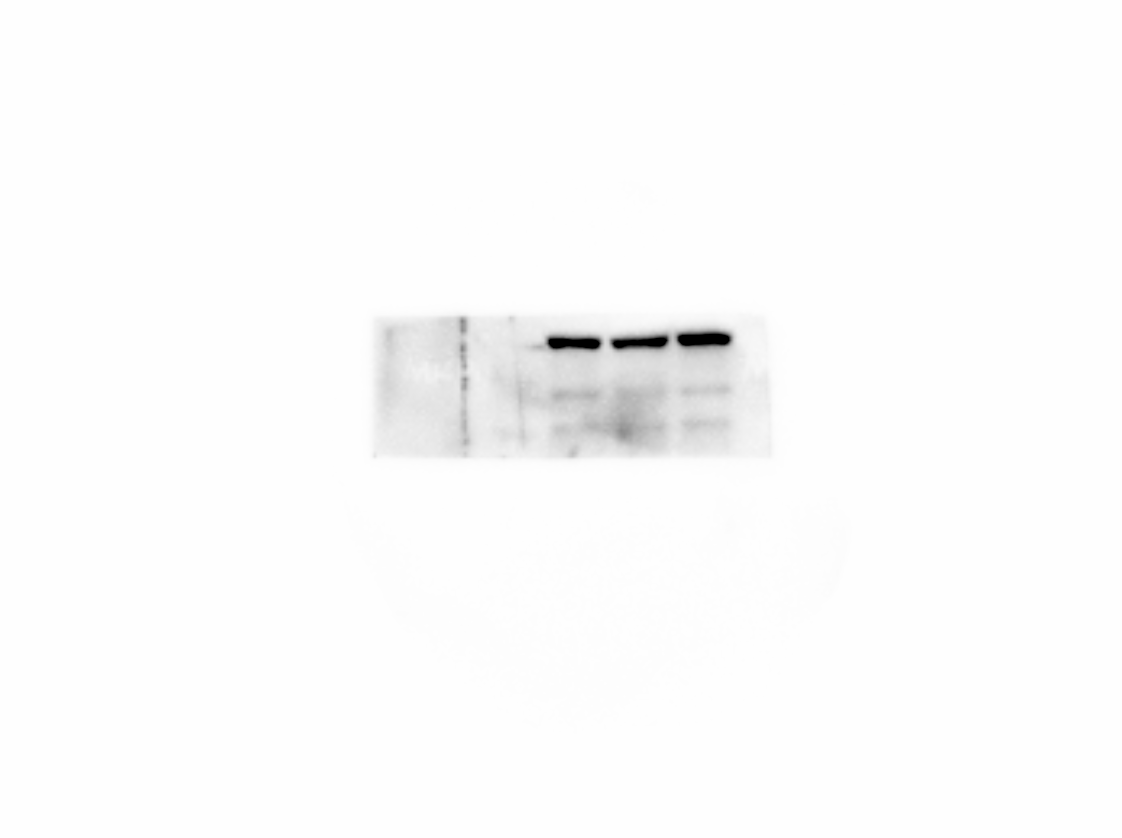


p-MLKL(Ser358): Control, t-BHP, PCB-95.


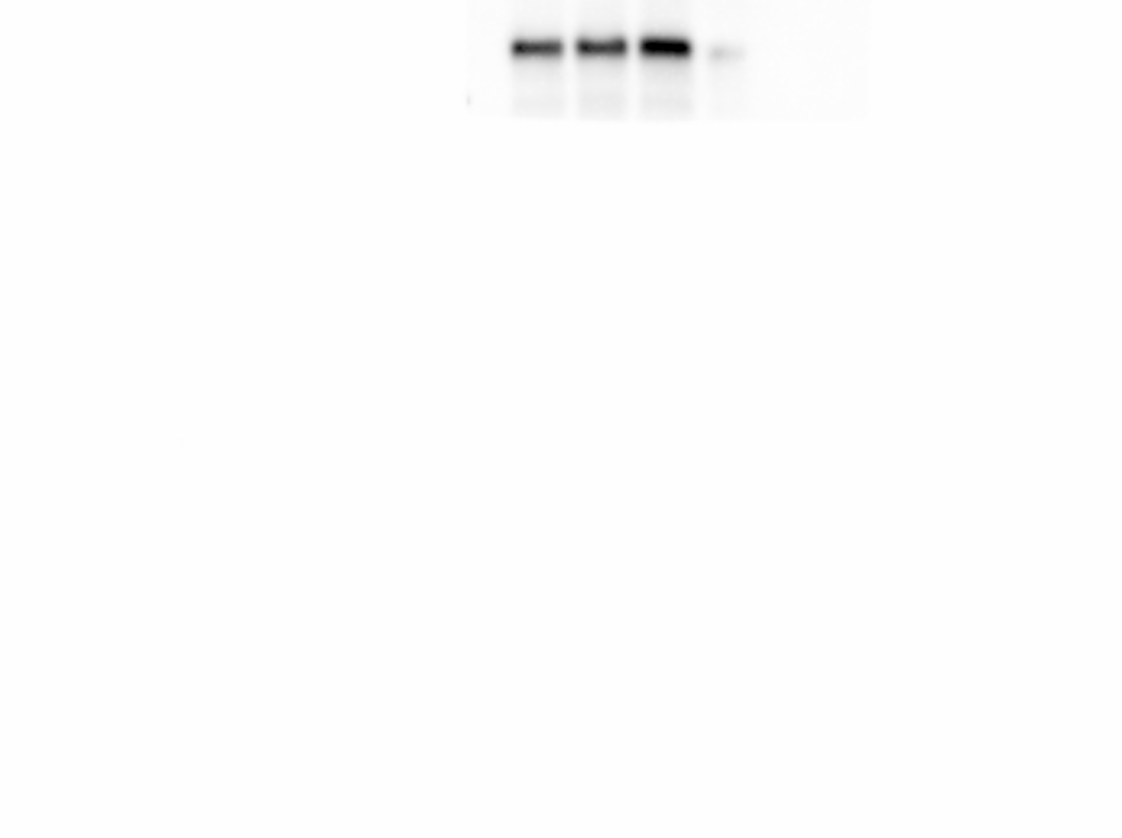


MLKL: Control, t-BHP, PCB-95.


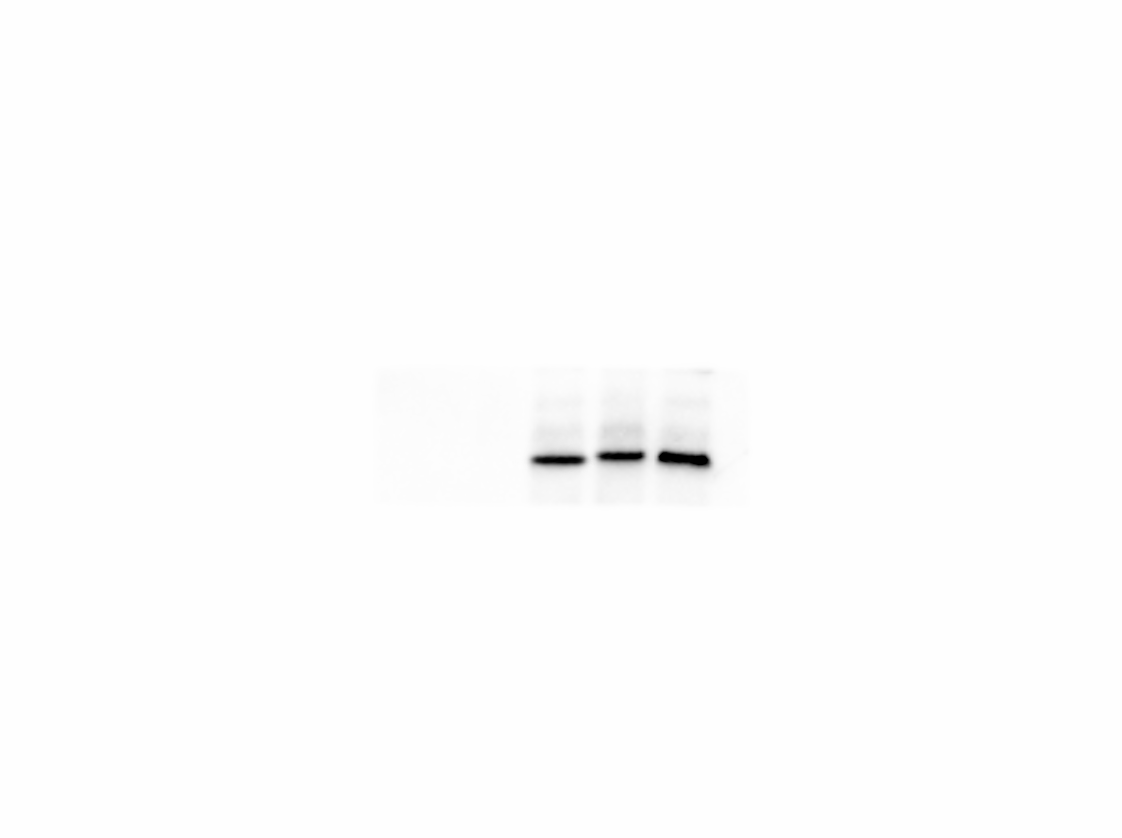


GAPDH: Control, t-BHP, PCB-95.


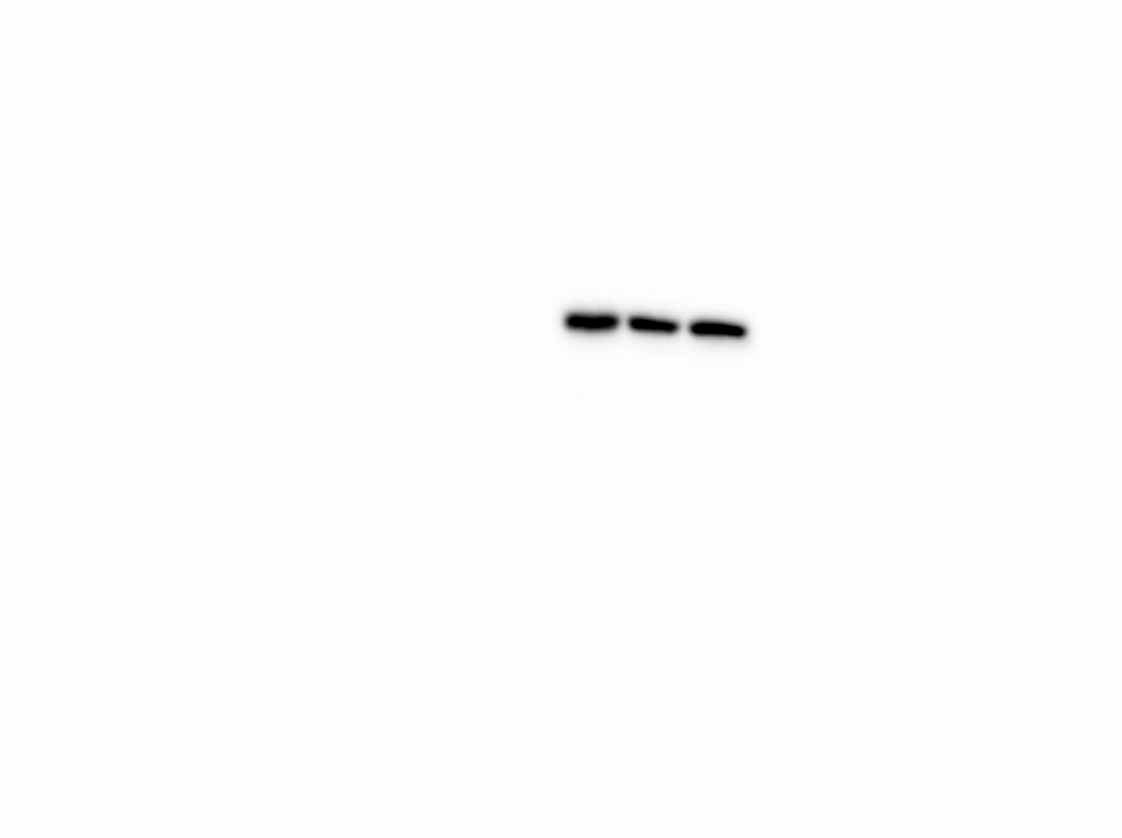


The full-length blots of Figure 7.

Figure 7 B

Gpx4: Control, t-BHP, SP600125+t-BHP, U0126+t-BHP


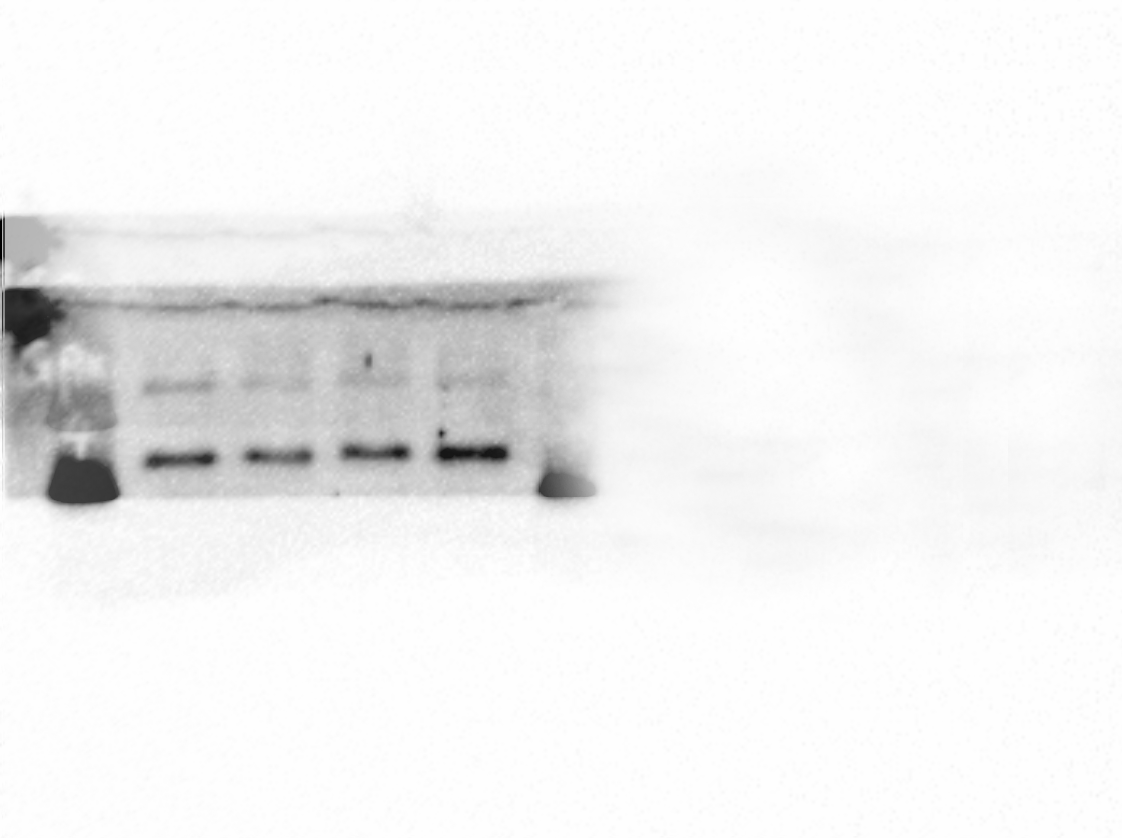


GAPDH: Control, t-BHP, SP600125+t-BHP, U0126+t-BHP


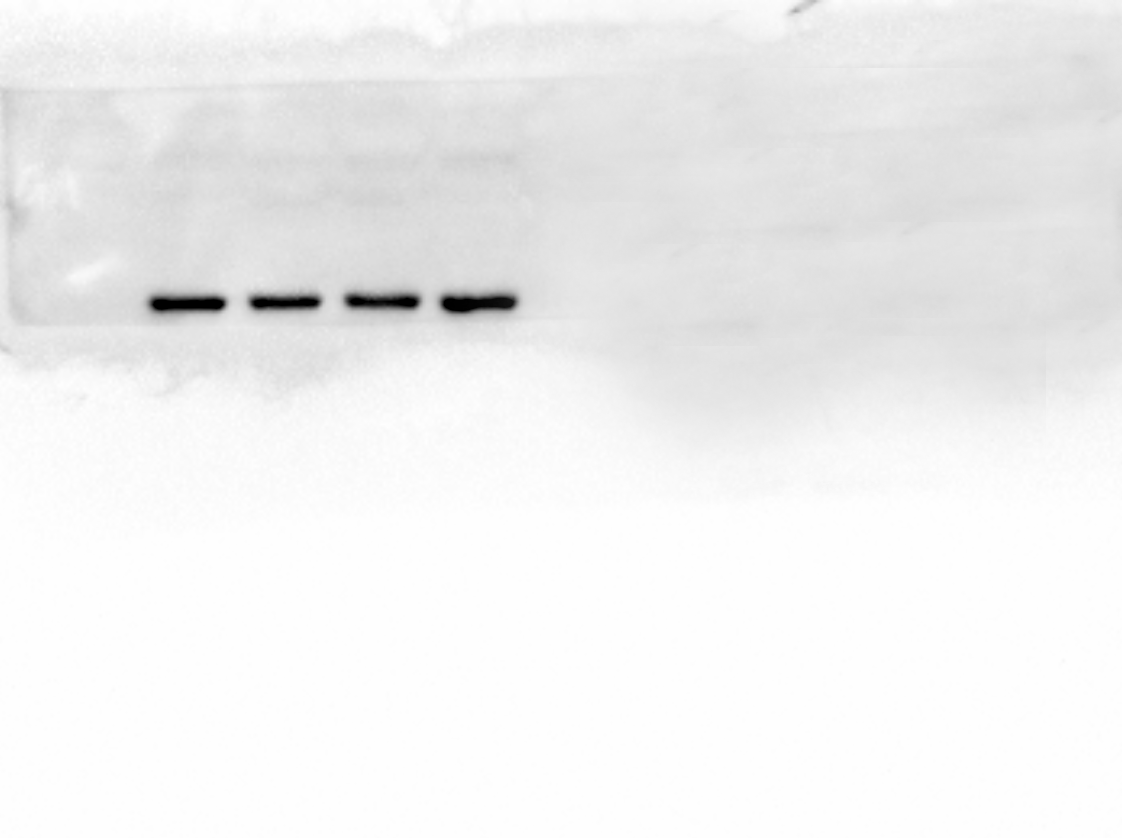


The full-length blots of Figure S3.

Figure S3 C

Caspas-7: Control, t-BHP


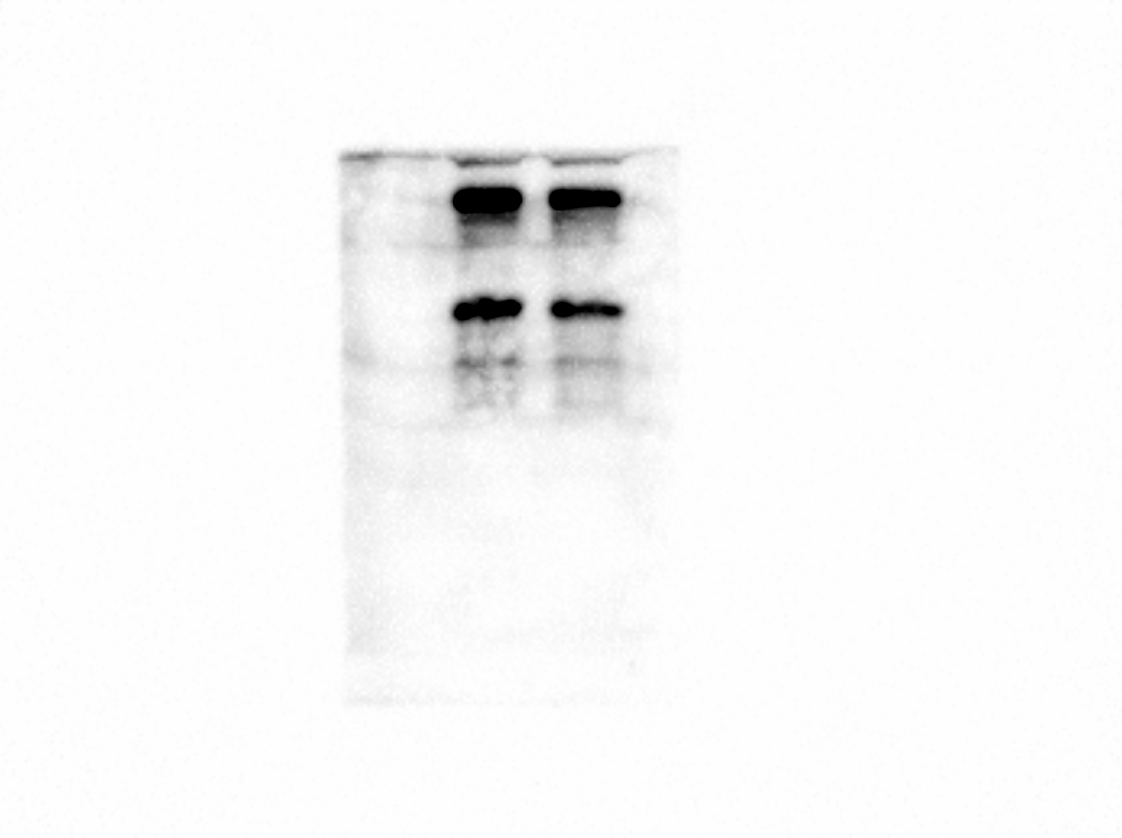


Cleaved-Caspase-7: Control, t-BHP


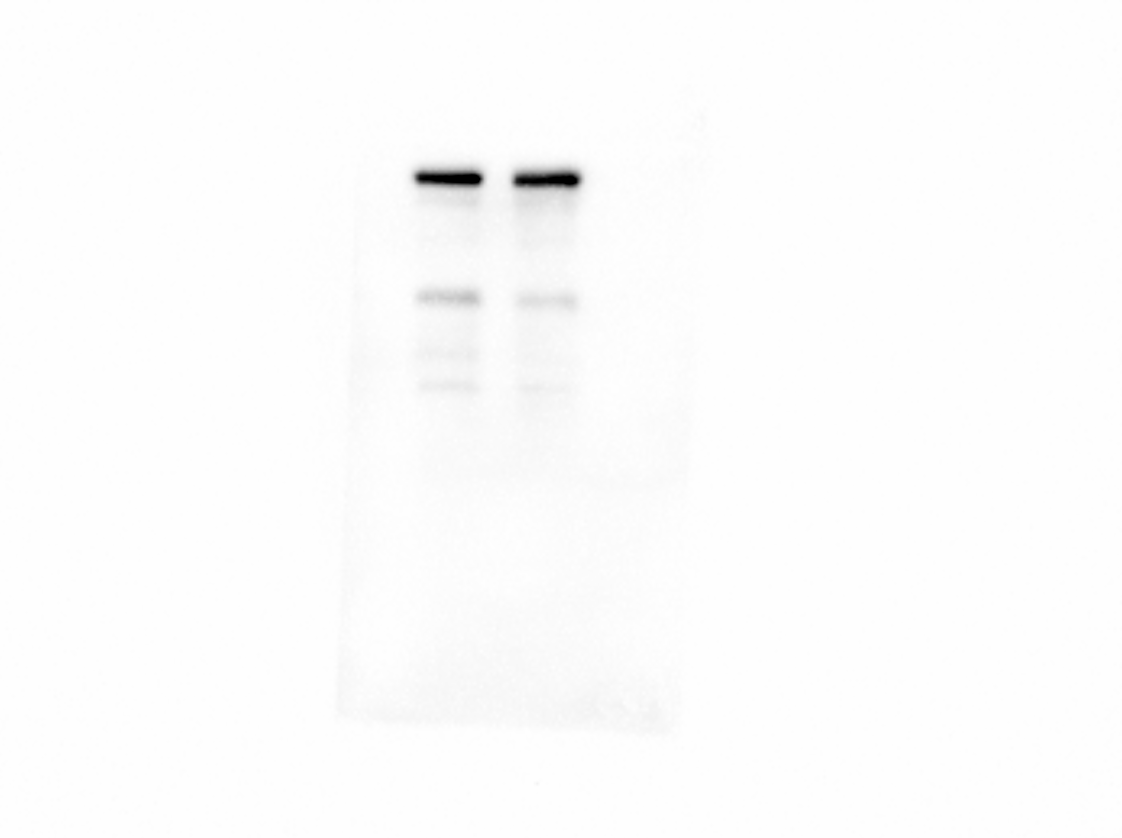


Caspase-3: Control, t-BHP


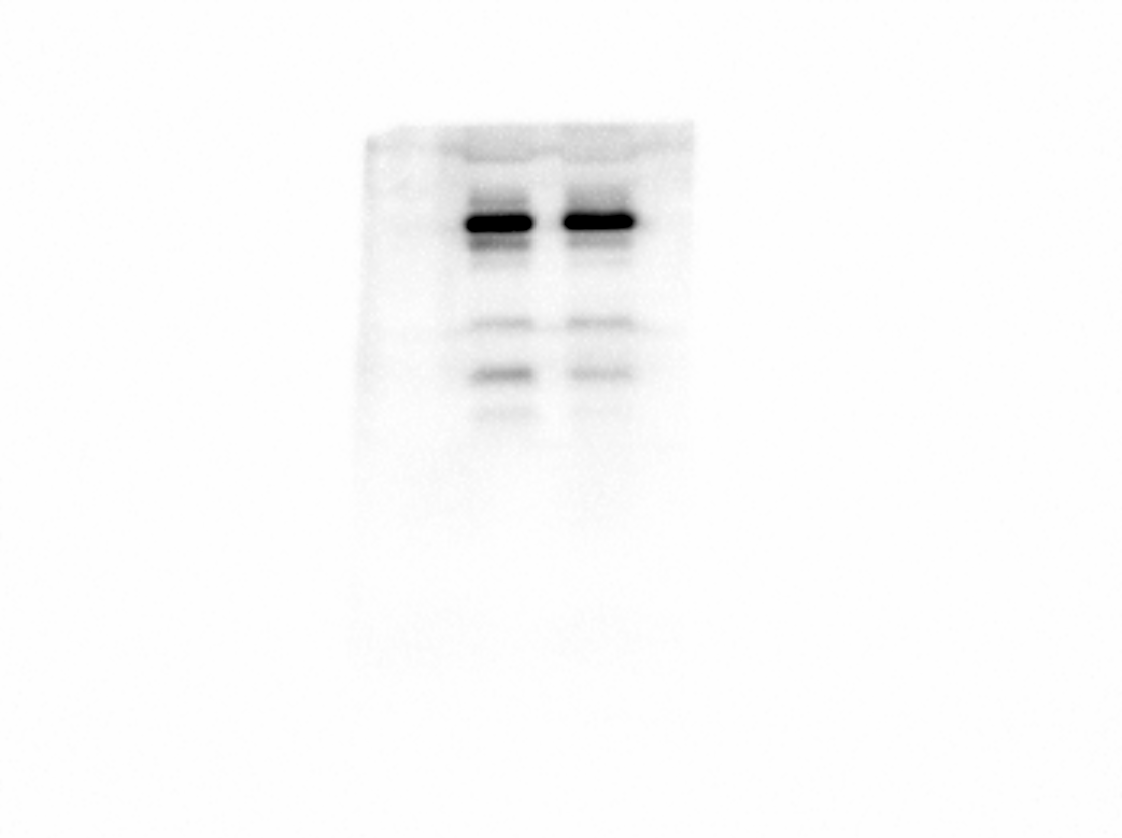


Cleaved-Caspase-3: Control, t-BHP


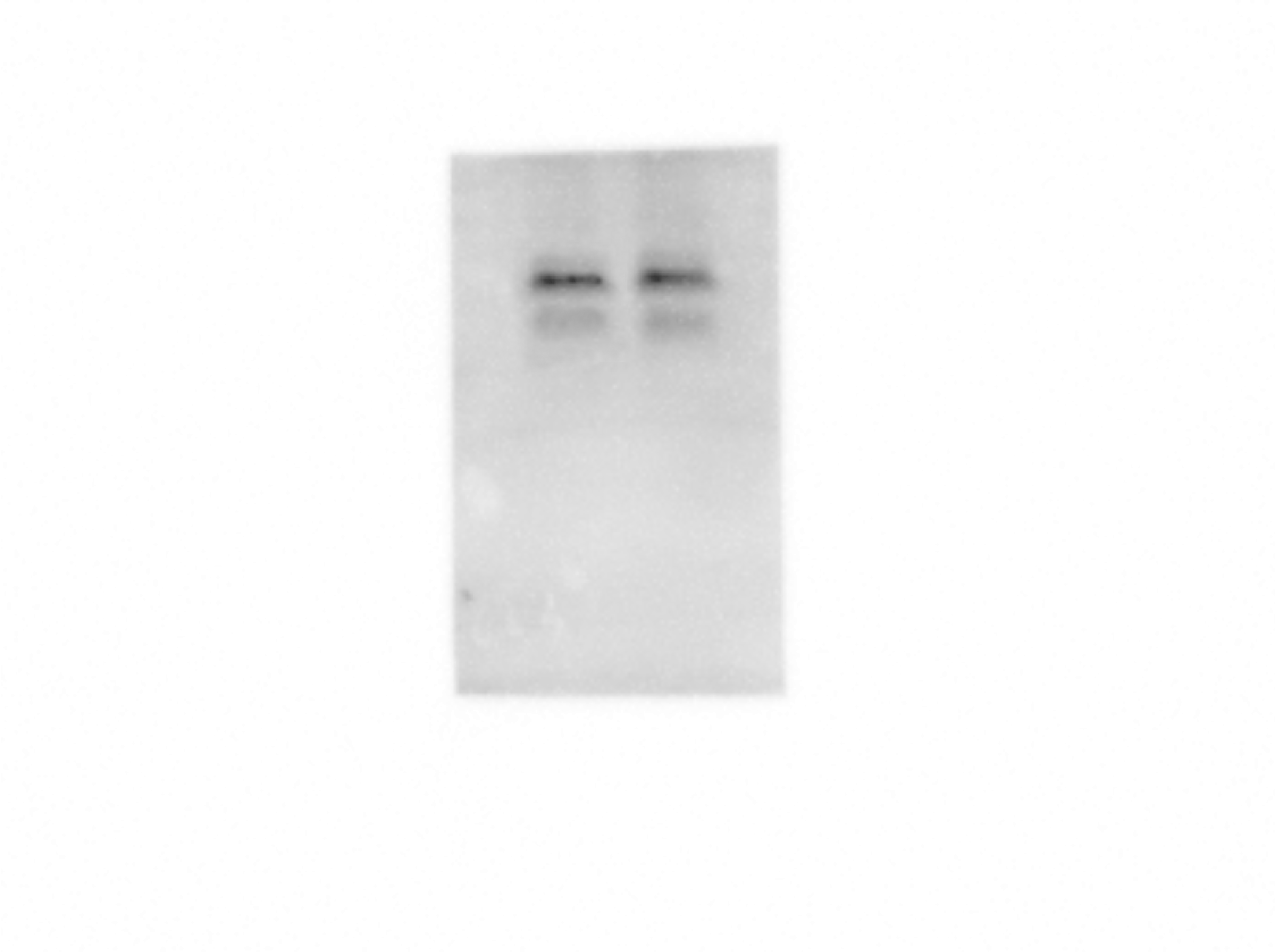


XIAP: Control, t-BHP


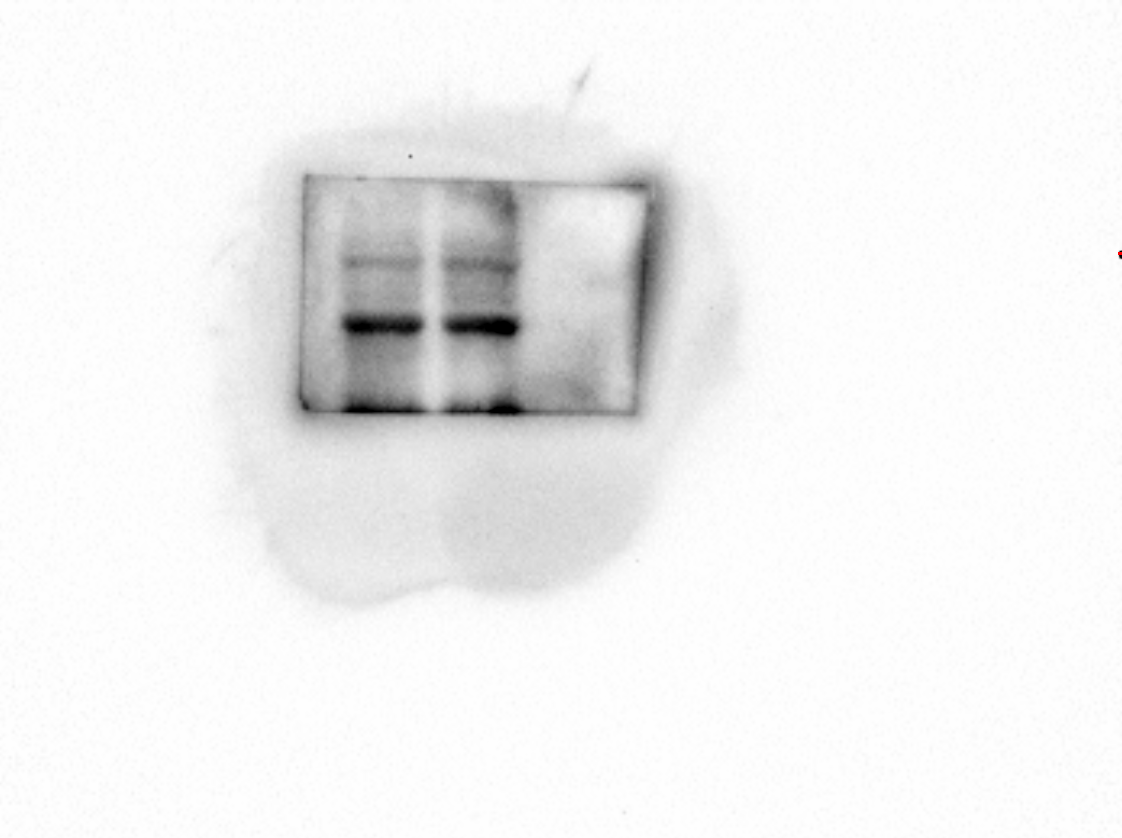


C-IAP1: Control, t-BHP


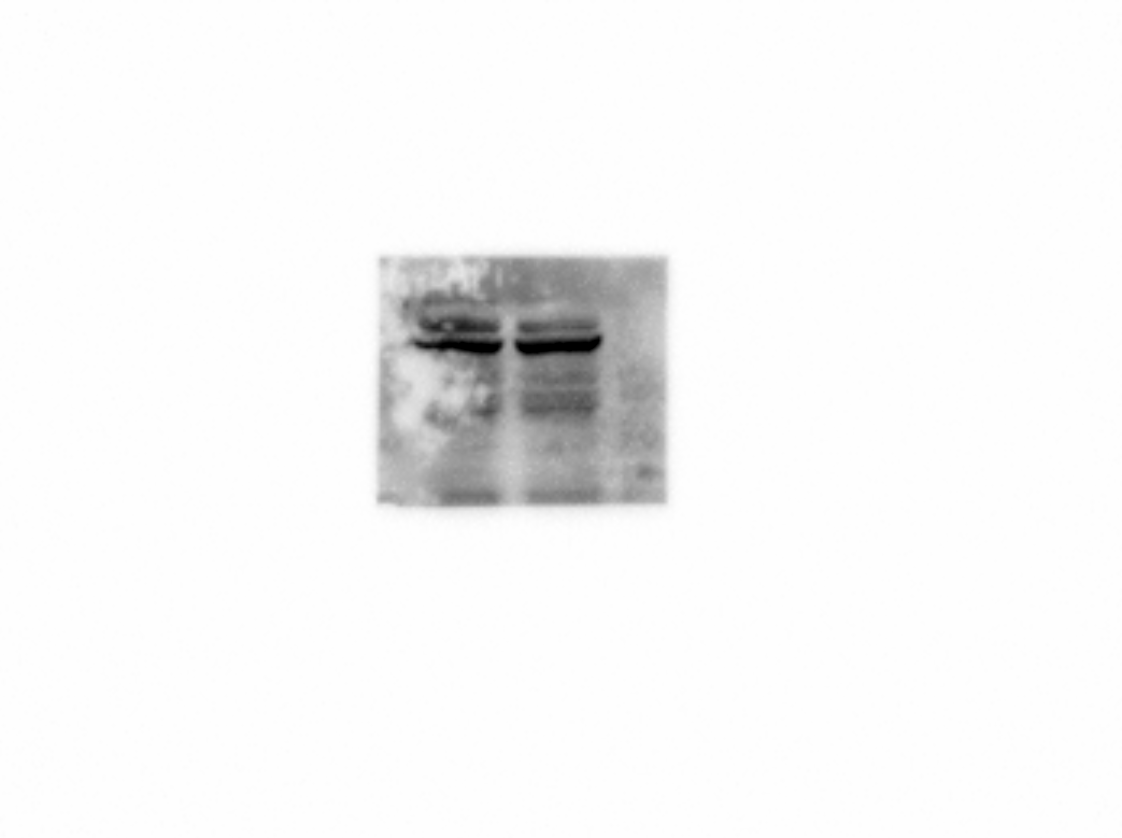


C-IAP2: Control, t-BHP


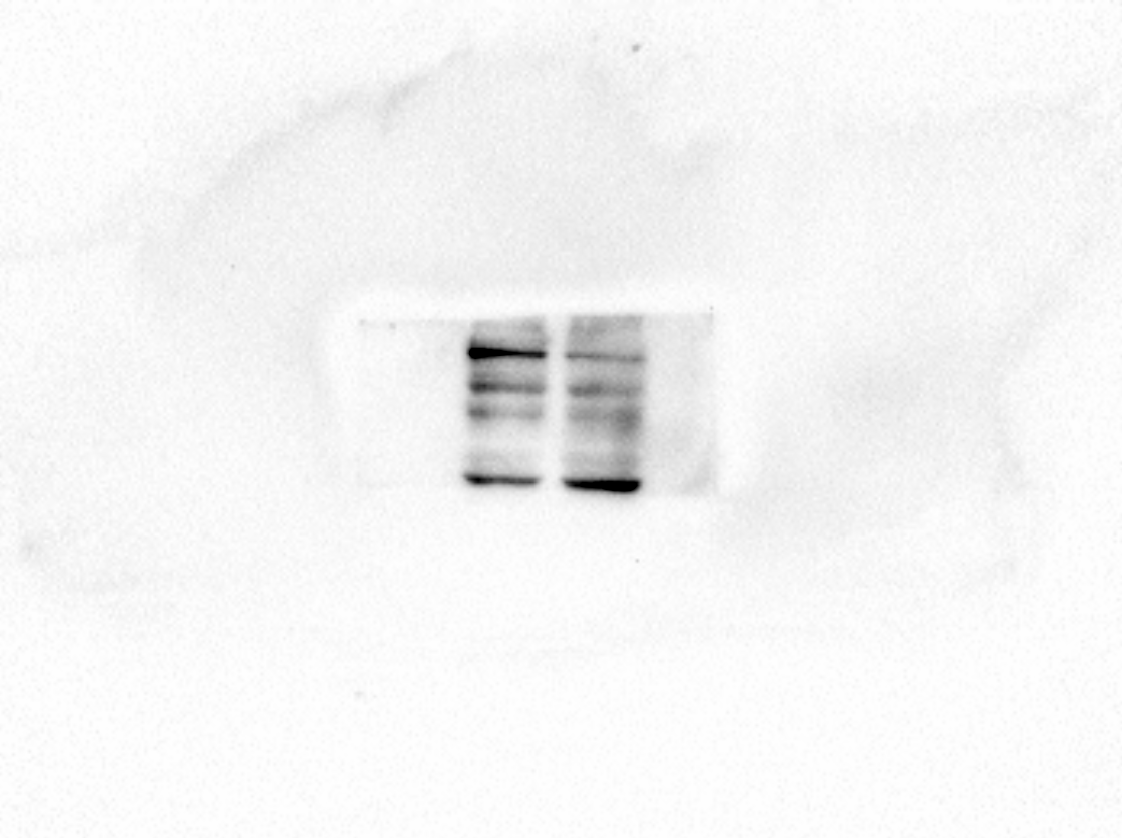


GAPDH: Control, t-BHP


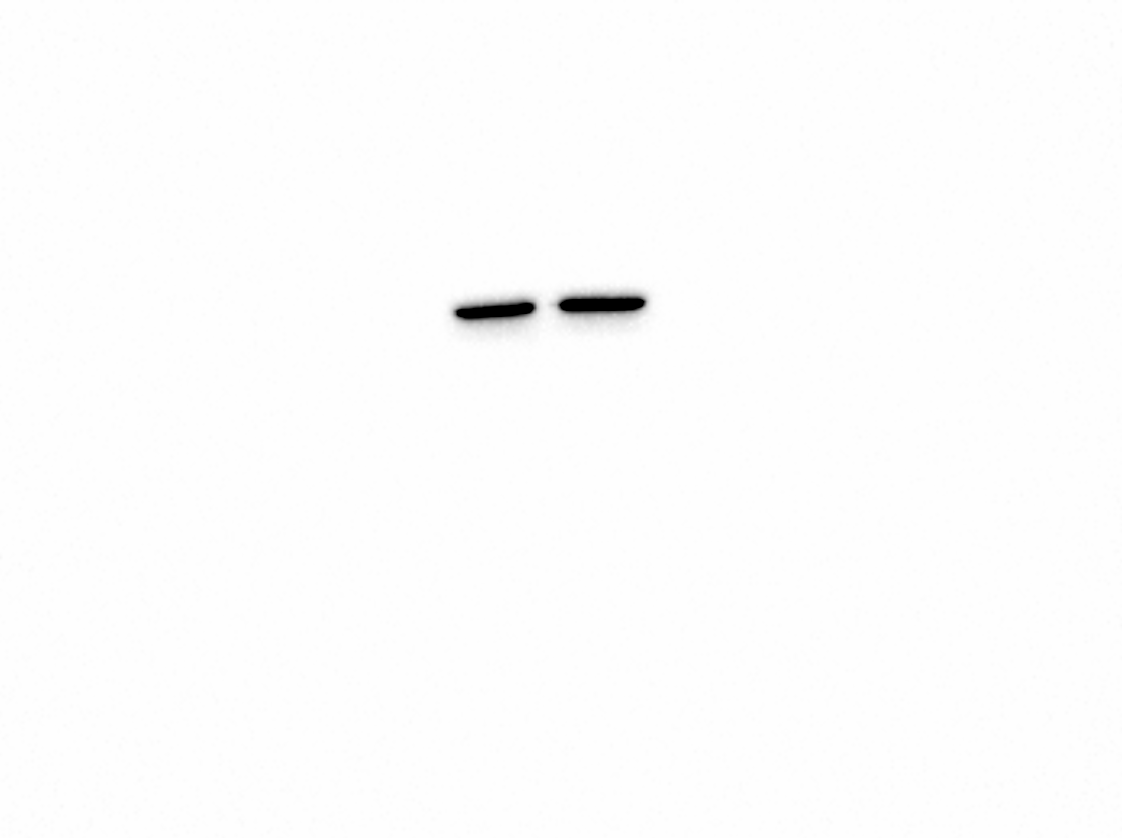


The full-length blots of Figure S5.

Figure S5 A

JNK: Control, t-BHP (0 min); Control, t-BHP (15 min); Control, t-BHP (30 min); Control, t-BHP (45 min); Control, t-BHP (60 min); Control, t-BHP (120 min).


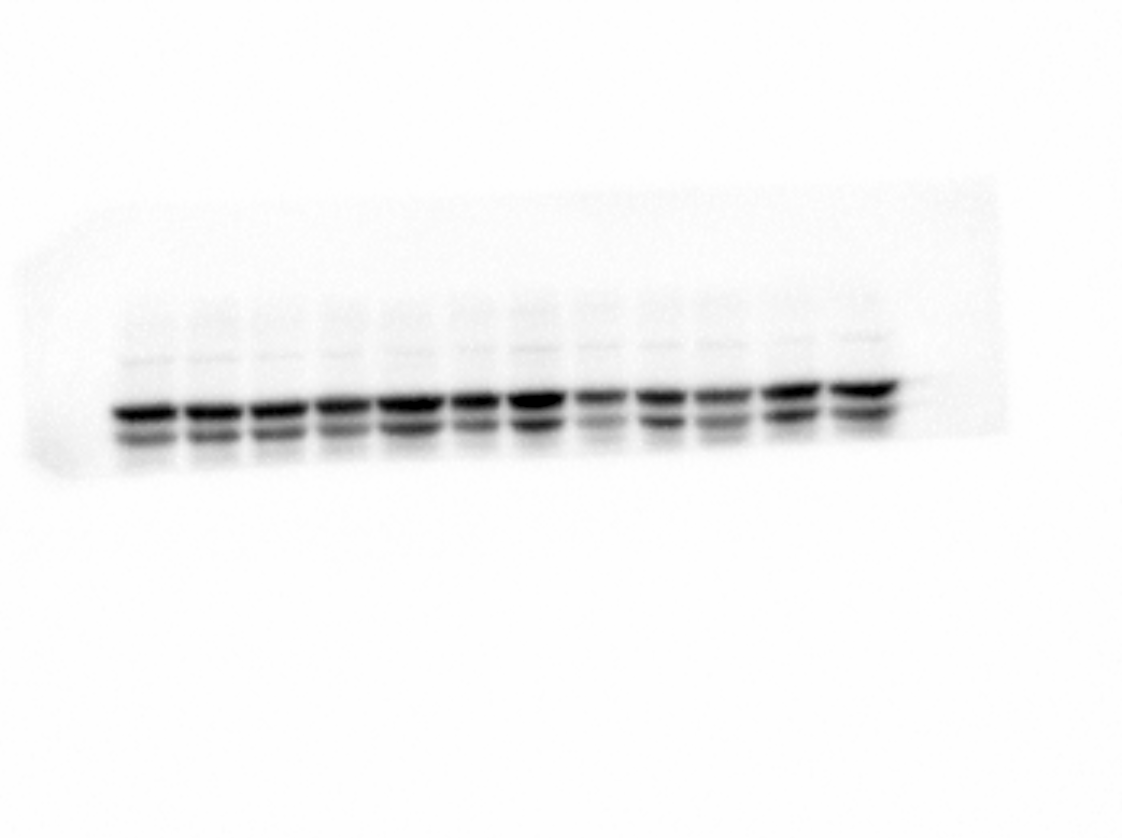


P-JNK: Control, t-BHP (0 min); Control, t-BHP (15 min); Control, t-BHP (30 min); Control, t-BHP (45 min); Control, t-BHP (60 min); Control, t-BHP (120 min).


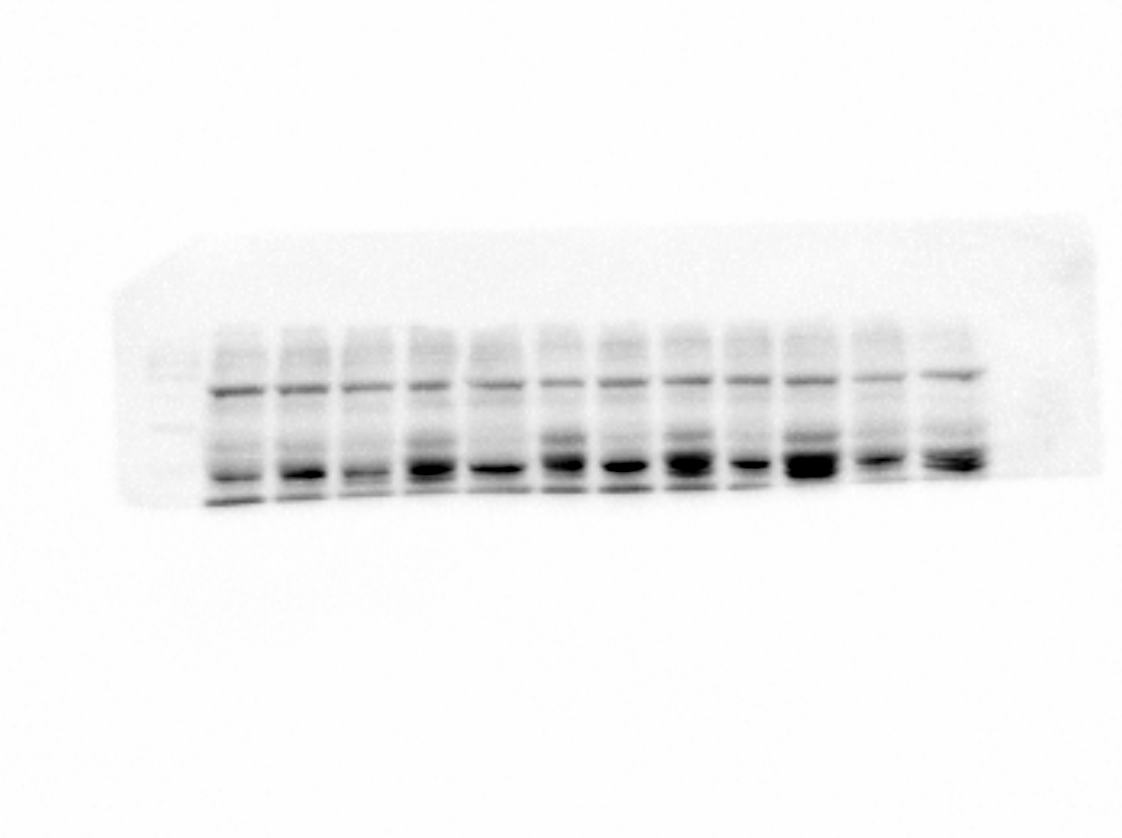


ERK: Control, t-BHP (0 min); Control, t-BHP (15 min); Control, t-BHP (30 min); Control, t-BHP (45 min); Control, t-BHP (60 min); Control, t-BHP (120 min).


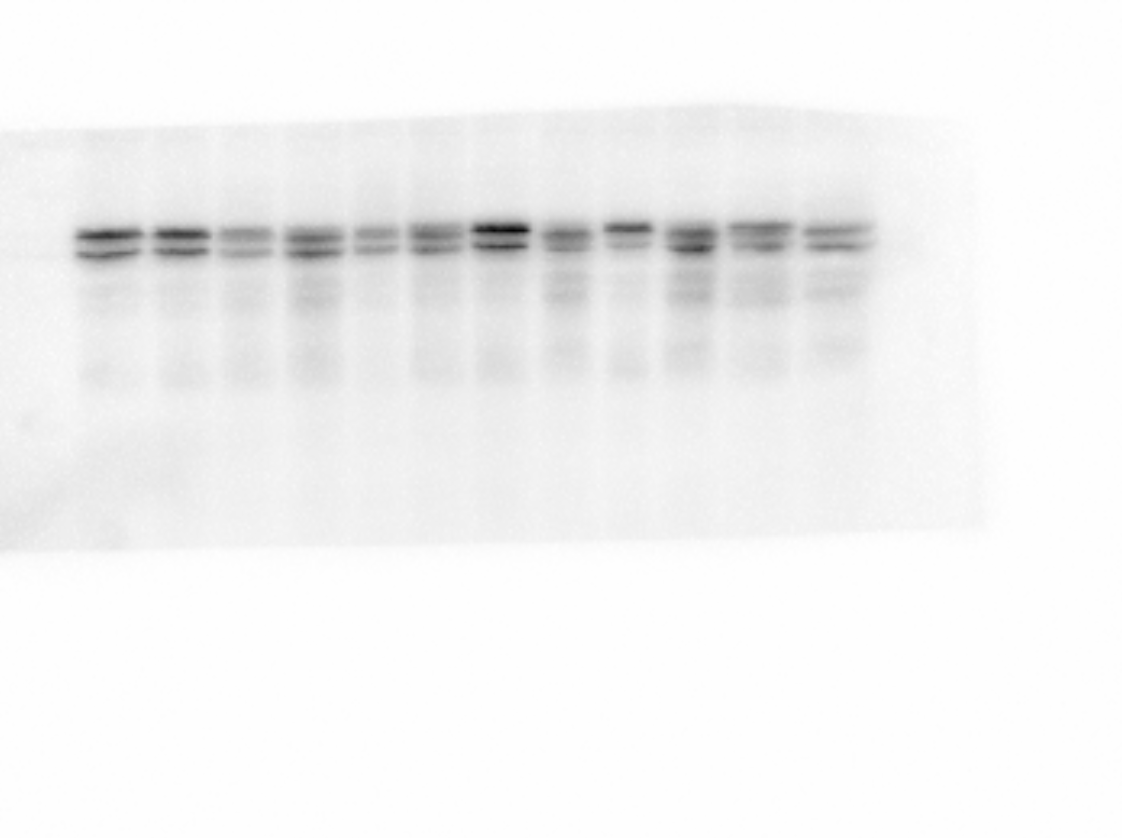


P-ERK: Control, t-BHP (0 min); Control, t-BHP (15 min); Control, t-BHP (30 min); Control, t-BHP (45 min); Control, t-BHP (60 min); Control, t-BHP (120 min).


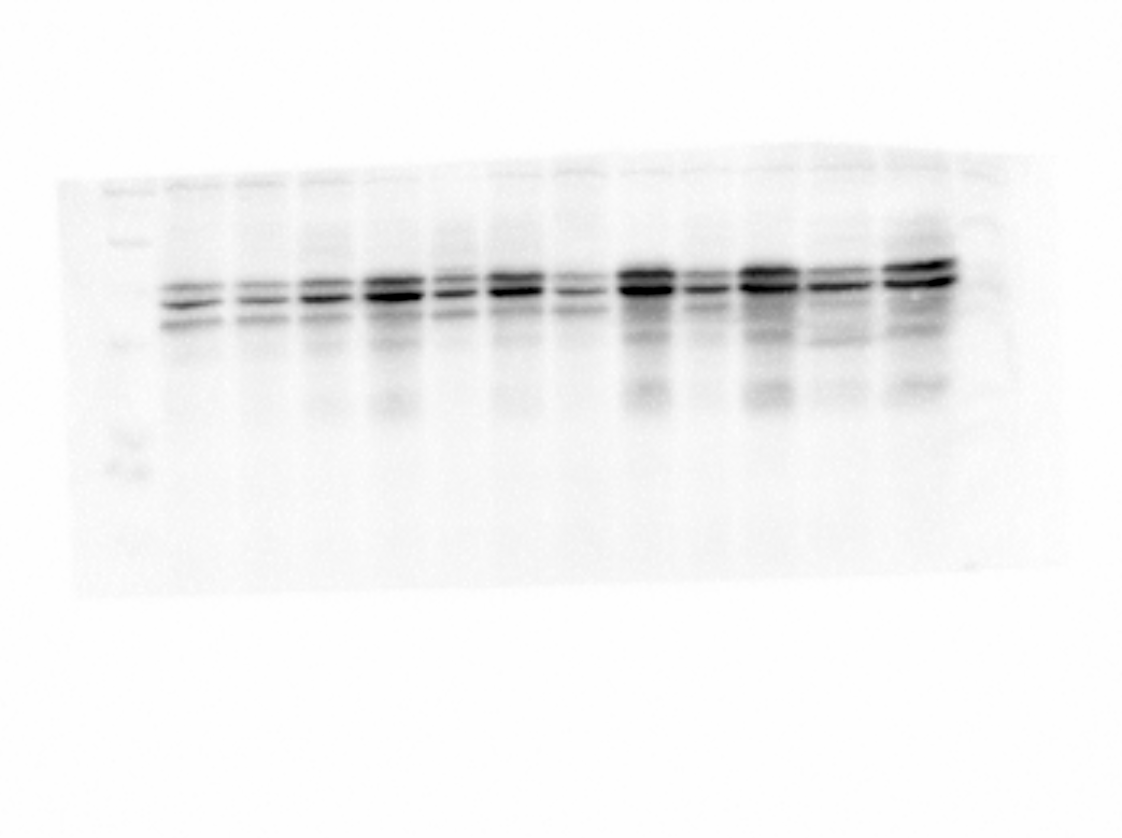


P38: Control, t-BHP (0 min); Control, t-BHP (15 min); Control, t-BHP (30 min); Control, t-BHP (45 min); Control, t-BHP (60 min); Control, t-BHP (120 min).


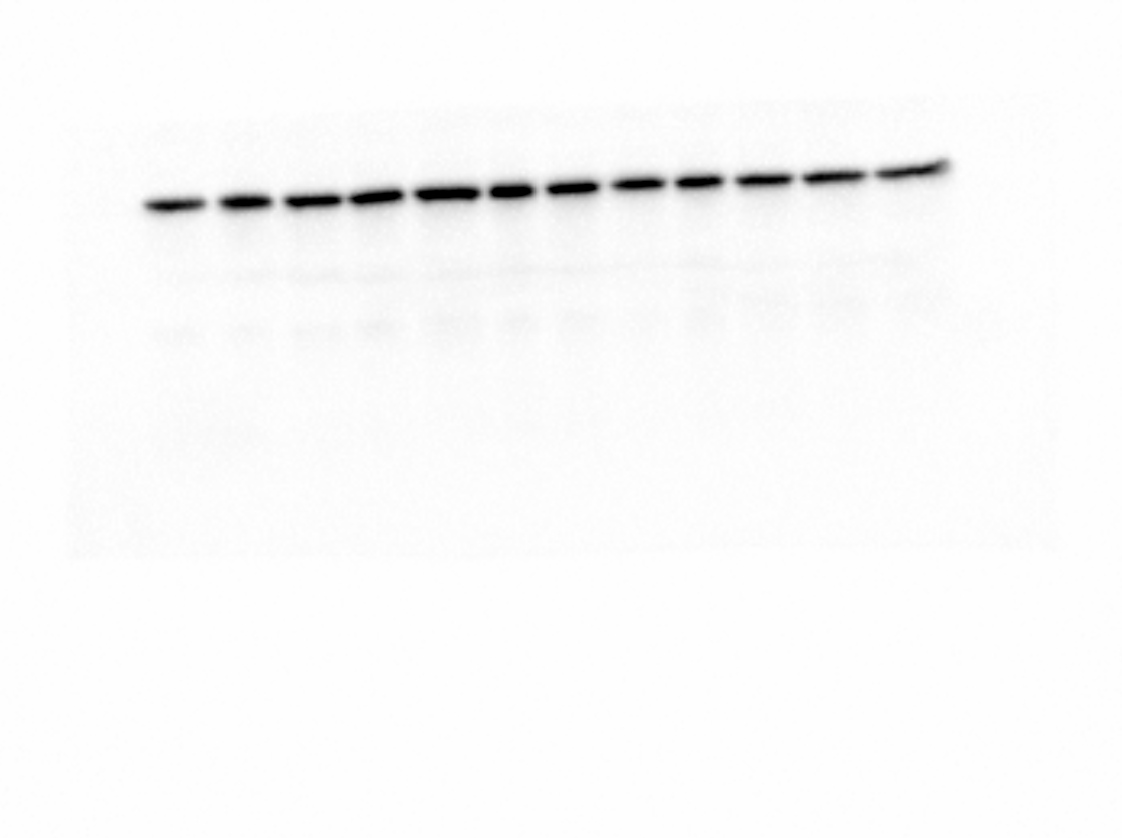


P-p38: Control, t-BHP (0 min); Control, t-BHP (15 min); Control, t-BHP (30 min); Control, t-BHP (45 min); Control, t-BHP (60 min); Control, t-BHP (120 min).


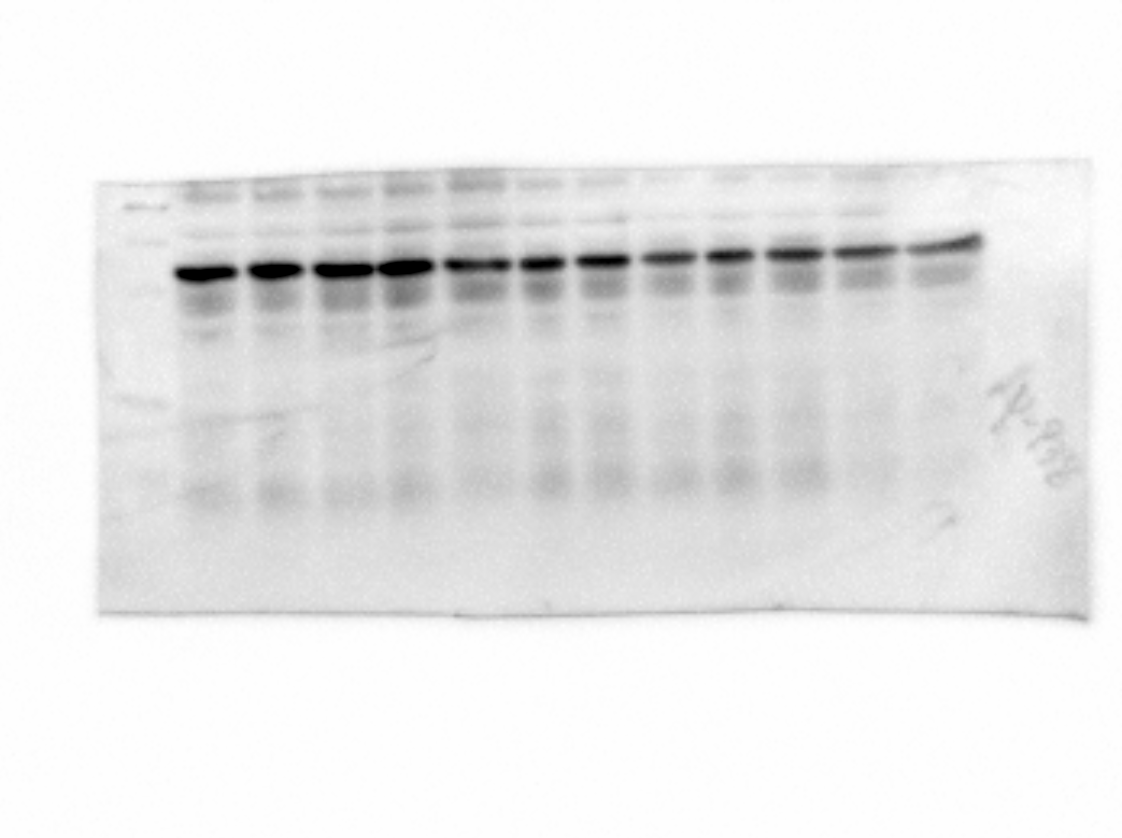


GAPDH: Control, t-BHP (0 min); Control, t-BHP (15 min); Control, t-BHP (30 min); Control, t-BHP (45 min); Control, t-BHP (60 min); Control, t-BHP (120 min).


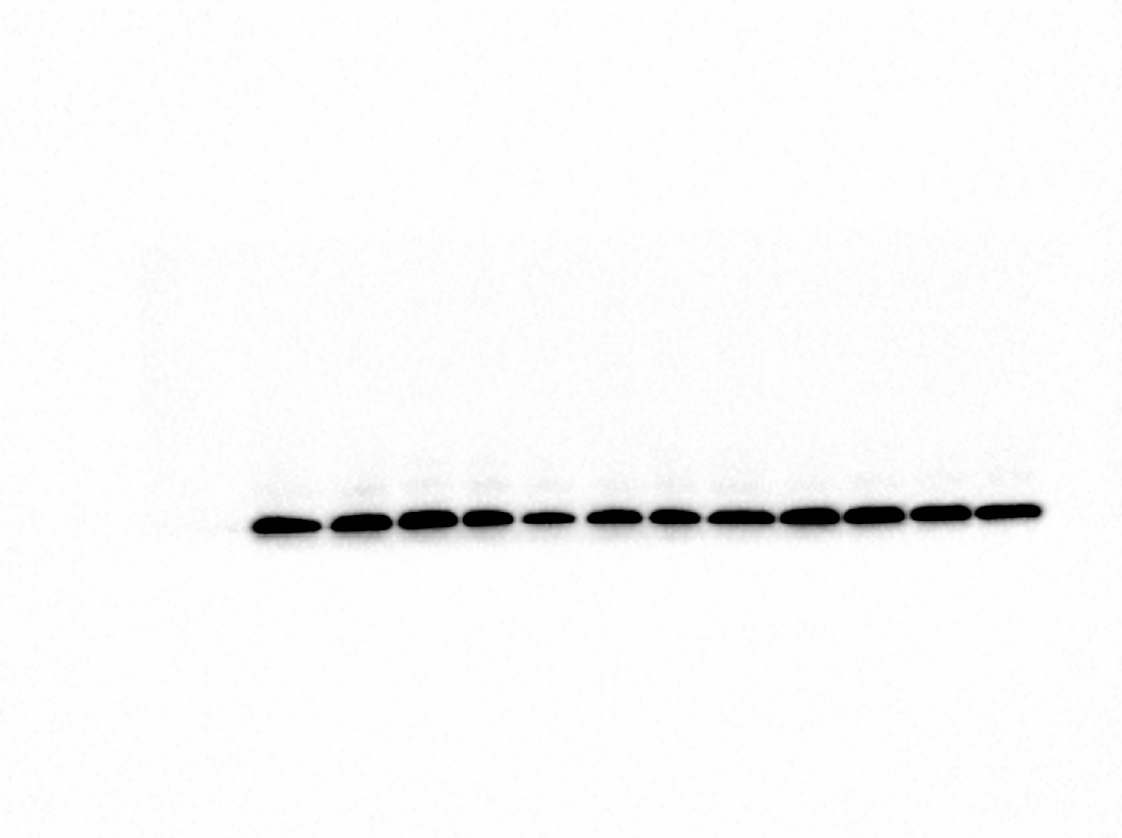


2. Details for antibodies.

| **Antibody** | **Cat. No.** | **Company** | **Dilutions** |
| --- | --- | --- | --- |
| Gpx4 | Ab219592 | Abcam | 1:1000 |
| Caspase-3 | 9665s | Cell Signaling Technology | 1:1000 |
| Cleaved-caspase-3 | 9661s | Cell Signaling Technology | 1:1000 |
| Caspase-7 | 9492s | Cell Signaling Technology | 1:1000 |
| Cleaved-caspase-7 | 8438s | Cell Signaling Technology | 1:1000 |
| Caspase-9 | 9502s | Cell Signaling Technology | 1:1000 |
| Cleaved-caspase-9 | 7237s | Cell Signaling Technology | 1:1000 |
| XIAP | 14334s | Cell Signaling Technology | 1:1000 |
| c-IAP1 | 7065s | Cell Signaling Technology | 1:1000 |
| c-IAP2 | 3130s | Cell Signaling Technology | 1:1000 |
| LC3B | 3868s | Cell Signaling Technology | 1:1000 |
| *p*-MLKL | 91689s | Cell Signaling Technology | 1:1000 |
| MLKL | 14993s | Cell Signaling Technology | 1:1000 |
| Bid | 2002s | Cell Signaling Technology | 1:1000 |
| Bcl-2 | 2870s | Cell Signaling Technology | 1:1000 |
| JNK | 9258s | Cell Signaling Technology | 1:1000 |
| *p*-JNK (Thr183/Tyr185) | 4668s | Cell Signaling Technology | 1:1000 |
| ERK | 4695s | Cell Signaling Technology | 1:1000 |
| *p*-ERK (Thr202/Tyr204) | 4370s | Cell Signaling Technology | 1:1000 |
| P38 | 8690s | Cell Signaling Technology | 1:1000 |
| *p*-p38 MAPK (Thr180/Tyr182) | 4511s | Cell Signaling Technology | 1:1000 |
| Cytochrome *c* | 4280s | Cell Signaling Technology | 1:1000 |
| COX IV | 4850s | Cell Signaling Technology | 1:1000 |
| GAPDH | 5174s | Cell Signaling Technology | 1:1000 |
| RIPK1 | 3279-100 | Abnova | 1:500 |
| RIPK3 | PAB2747 | Abnova | 1:500 |
| Transferrin Receptor (TFR) | 13-6800 | Invitrogen | 1:500 |

3.Quantitative results of FACS.

Figure 1 D


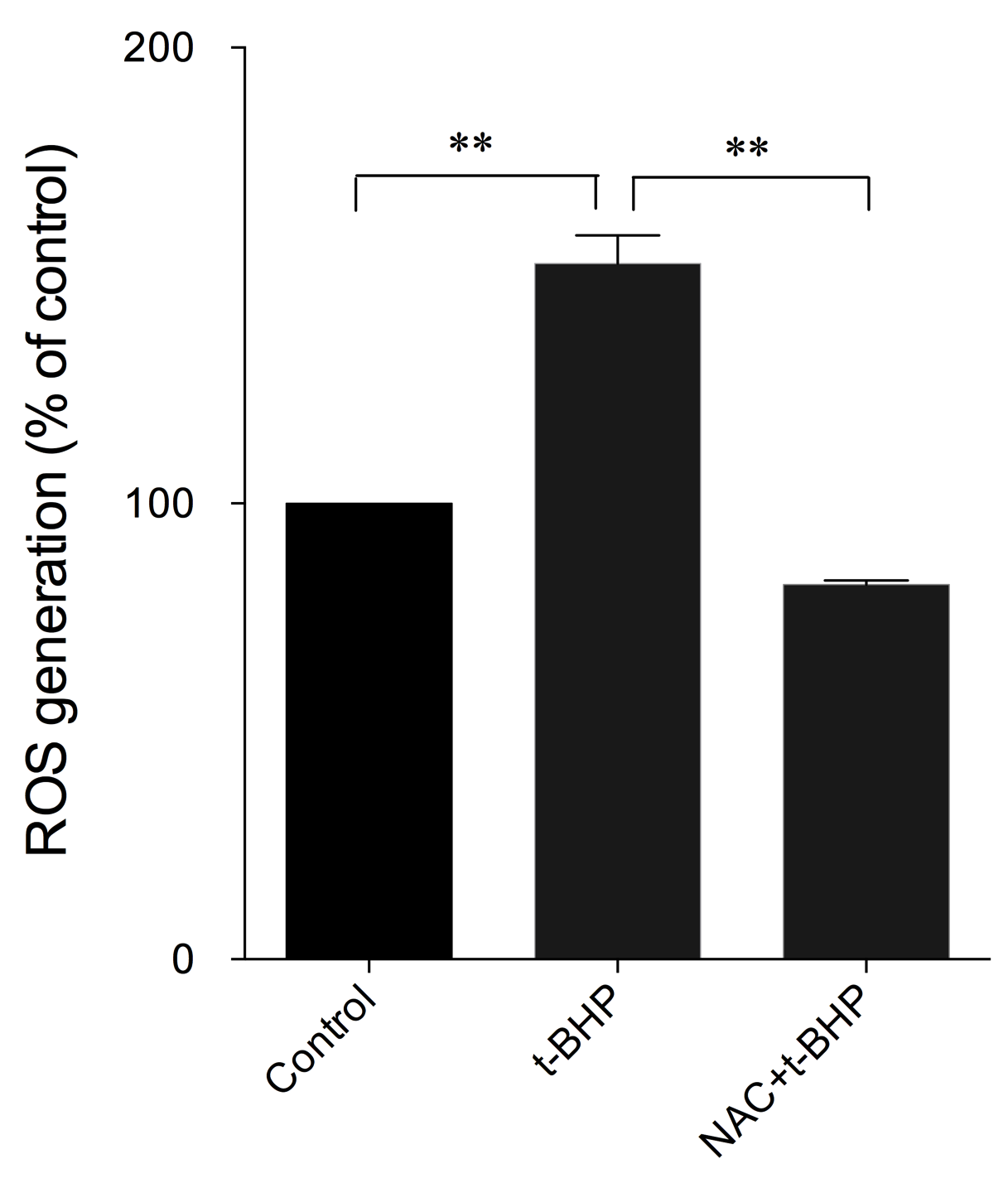


Figure 3 A


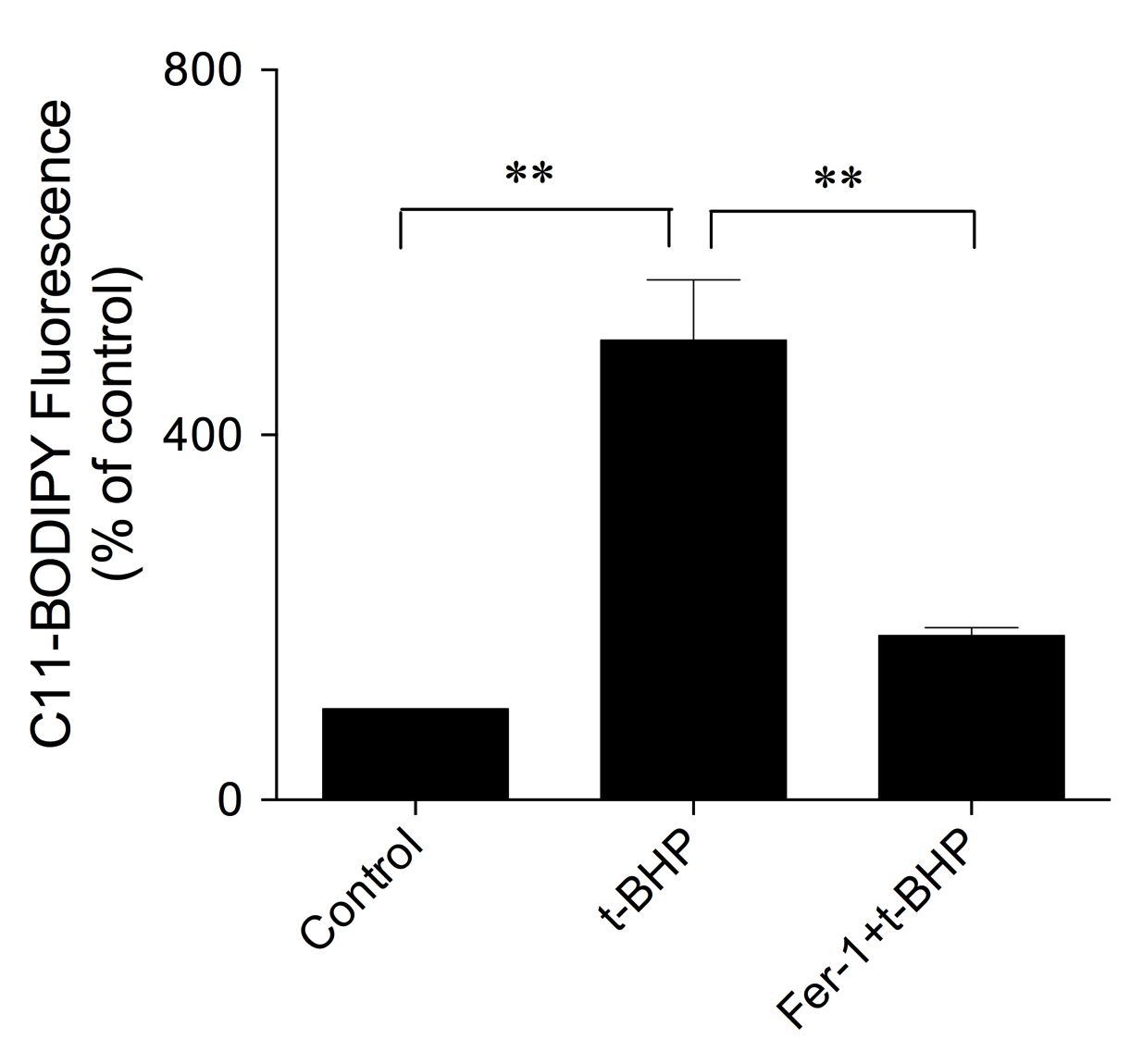


Figure 3 B


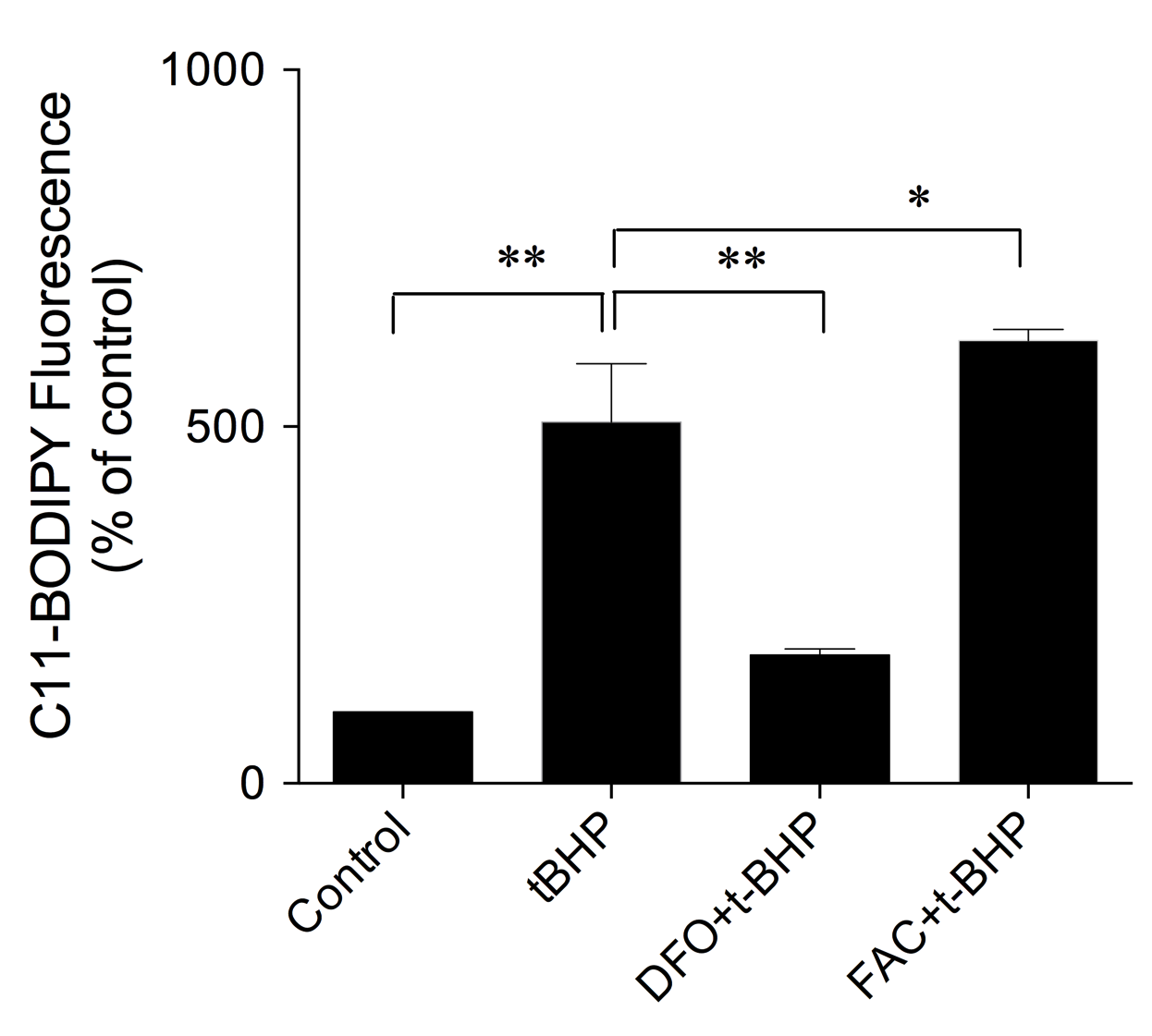


Figure 4 B


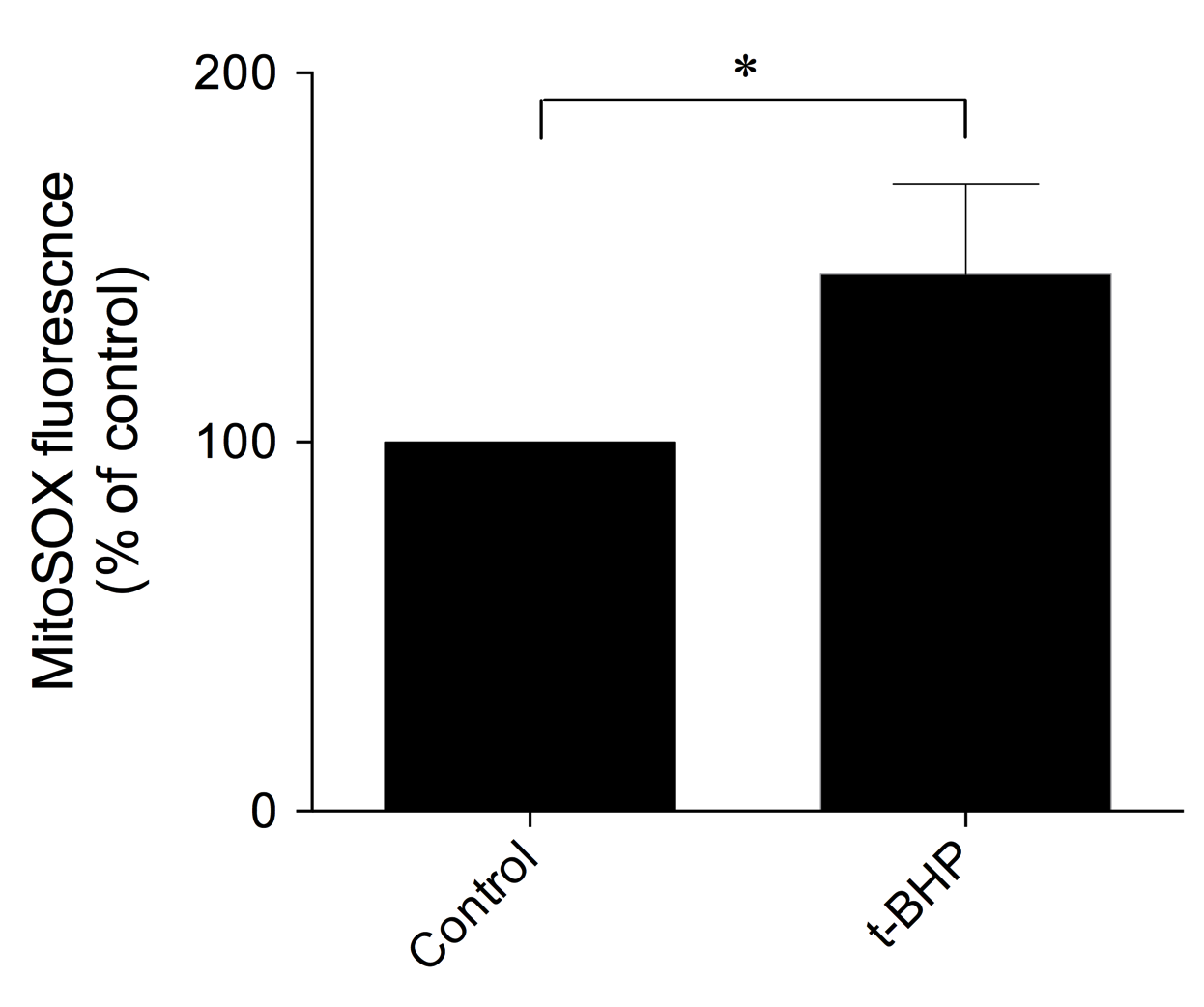


Figure 6 C


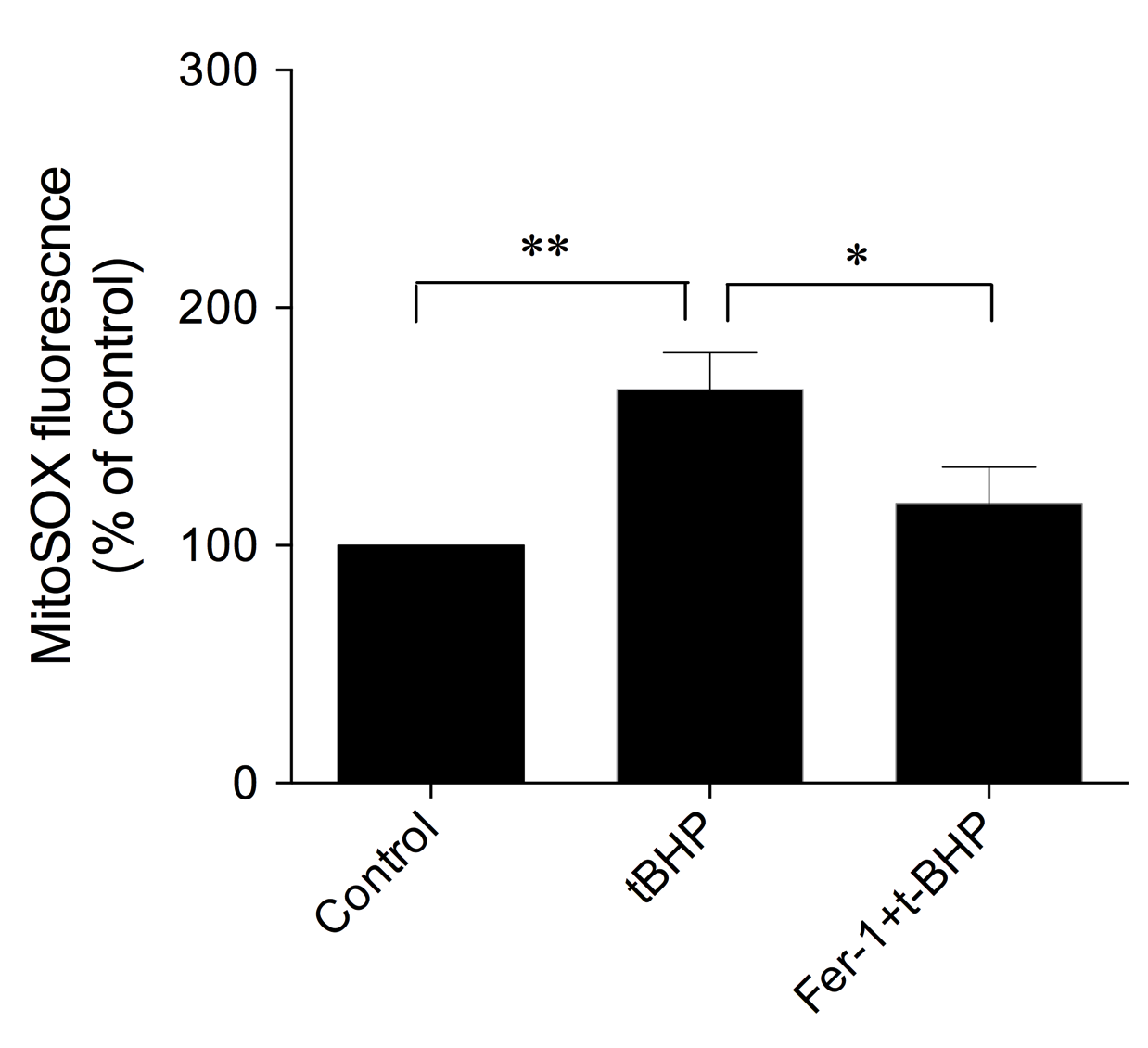


Figure 7 E


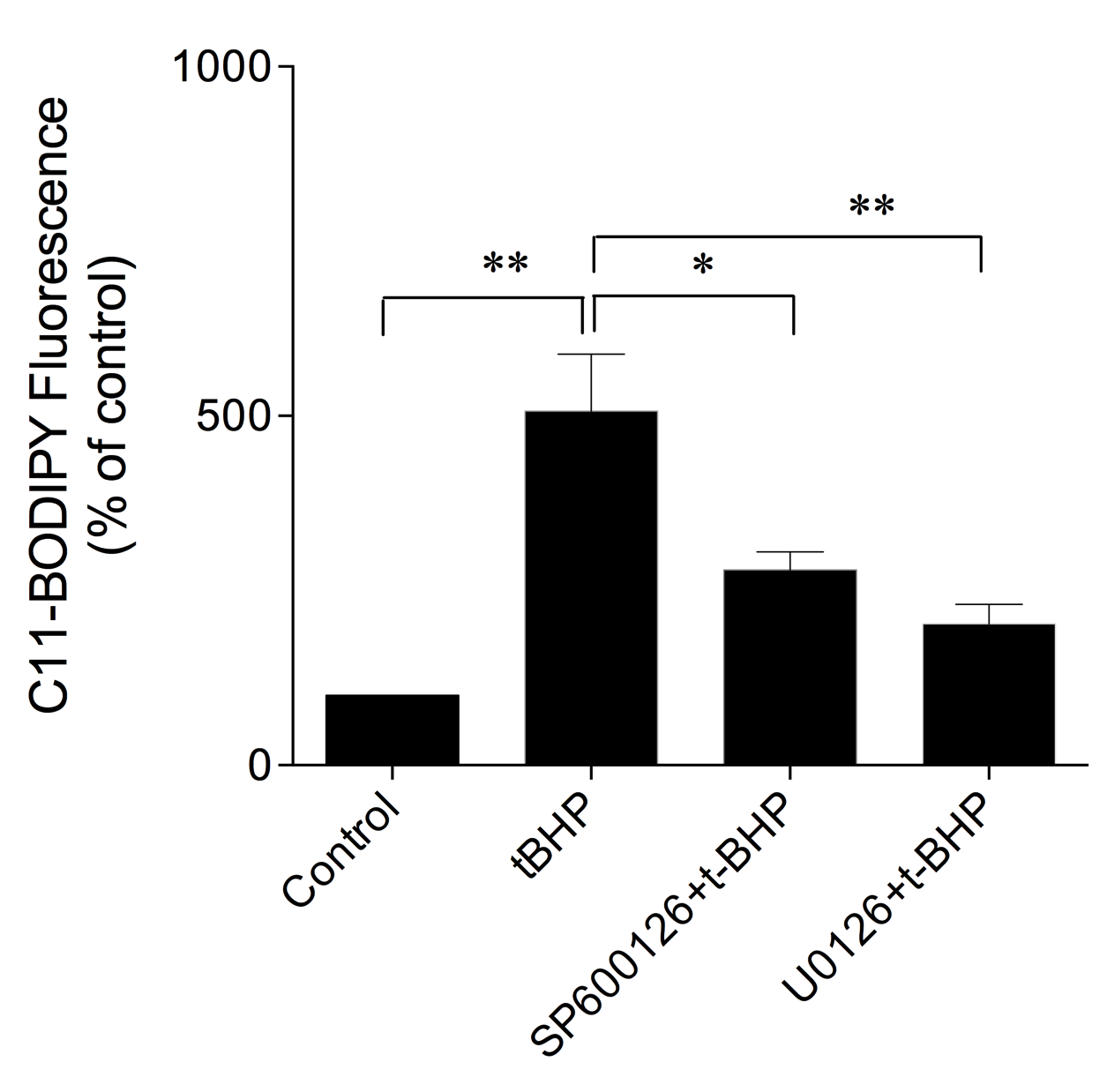


Figure 7 F


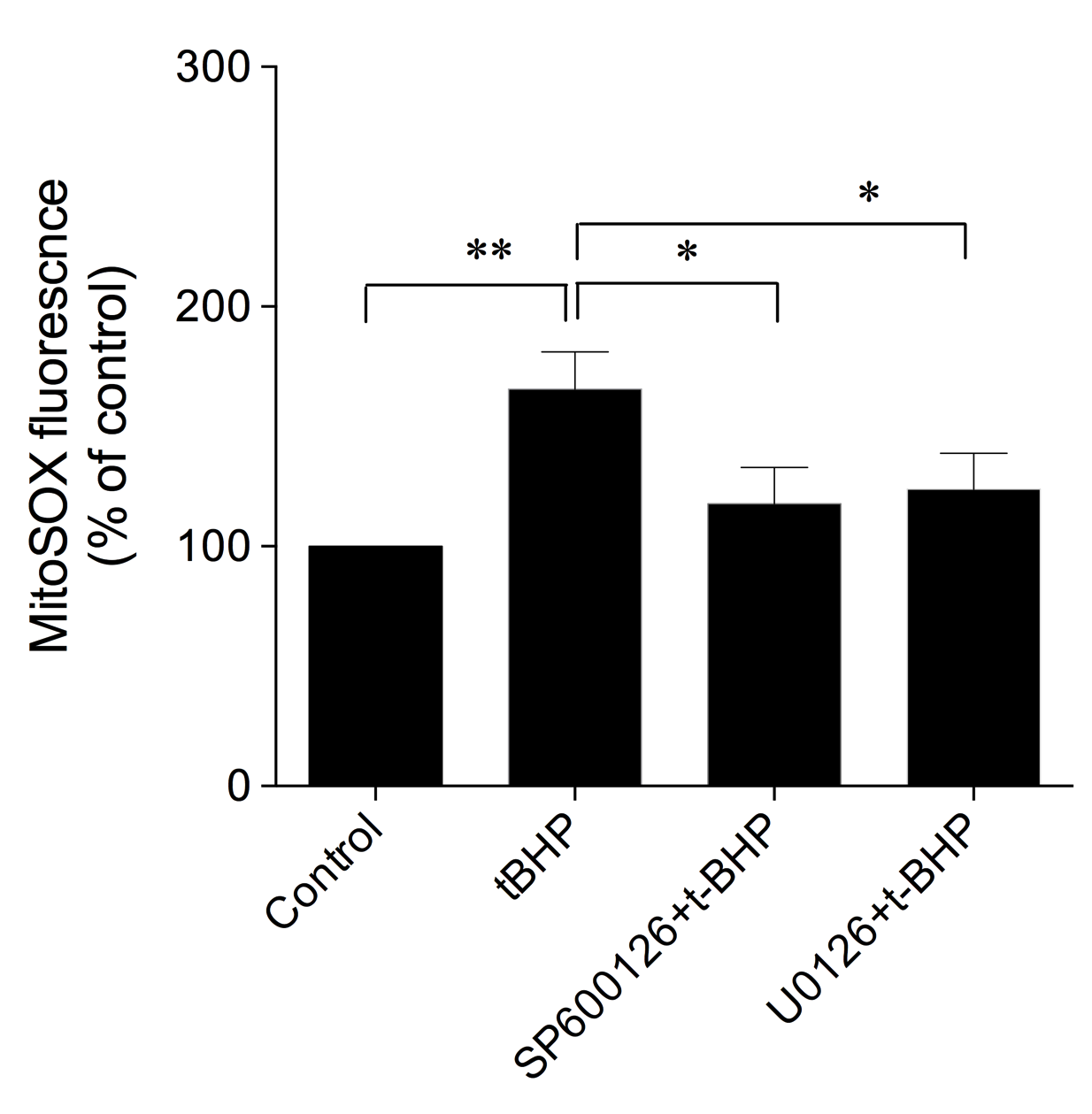


4. PC12 cells treated with t-BHP (50 μM, 100 μM, 200μM and 400 μM) for 0.5 h, 1 h, 2 h and 4 h, and then the dynamic continuous imaging results revealed that the cell morphology in the cells of t-BHP group at the concentration of 100 μM for 1 h, remained a contrast good condition. After this concentration and time point, the cells undergoing condensed nucleus and cytoplasm, accompanied with cell rounding-up (Figure S1A). Moreover, cell viability results revealed that t-BHP at the concentration of 100 μM for 1 h, caused about 50% percent of the cell loss (Figure S1B and C).


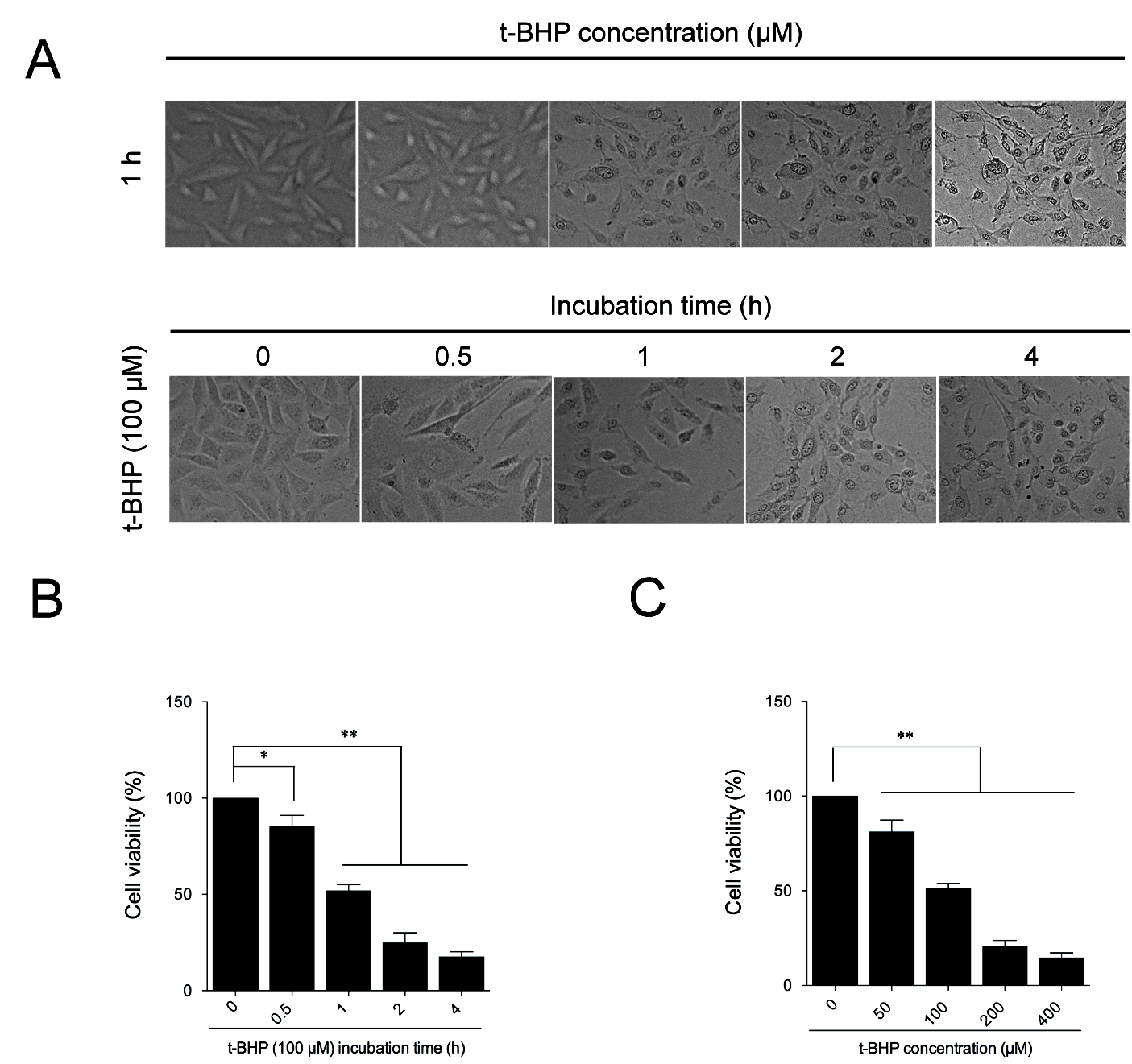


**Figure S1 Selection of the oxidative stress condition in PC12 cells.** PC12 cells were treated with t-BHP (50 μM, 100 μM, 200 μM and 400μM) for 0.5 h, 1 h, 2 h and 4 h, cell morphology (A), cell viability (B and C). ^**^*P*＜0.01, ^*^*P*＜0.05.

5. T-BHP treated cells accompanied with rounding-up morphology change, which was consistent with the morphology of the ferroptosis. Interestingly, Ferrostatin-1 (Fer-1, 1 μM), a specific inhibitor for ferroptosis, could dramatically restore t-BHP-induced morphological changes in PC12. Moreover, deferoxamine (DFO, 100 μM), which can decrease ferric irons, had the same effect as Fer-1. Meanwhile ammonium ferric citrate (FAC, 1 μg/ml) can provided ferric ions, but further aggravated the morphological changes induced by t-BHP (Figure S2 A, B and C). In addition, Fer-1 and DFO could enhance cell viability after treated with t-BHP, whereas FAC further reduced the cell viability (Figures S2 D, E, and F).
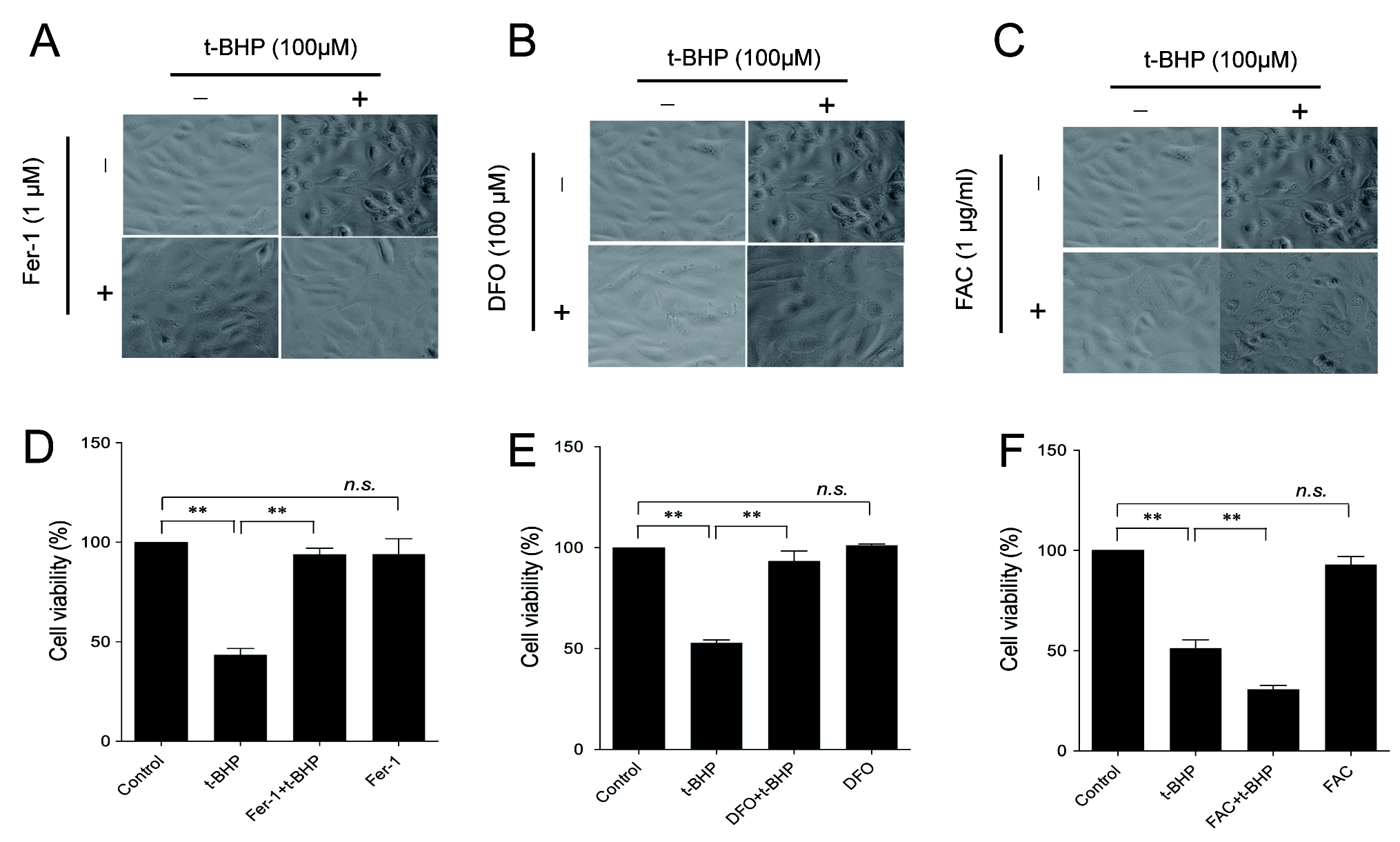


**Figure S2 Evidence of ferroptosis in PC12 cells after co-treatment with t-BHP** PC12 cells were treated with t-BHP (100 μM) for 1 h with or without Fer-1 (1 μM)/DFO (100 μM)/ FAC (1 μg·ml^-1^) pretreatment for 24 h. Morphological changes (A)-(C) and the cell viability (D) – (F) were observed and determined by microscopy and the MTT assay respectively. ^**^*P*＜0.01; ^*^*P*＜0.05; Fer-1, ferrostatin-1; DFO, deferoxamine; FAC, ferric ammonium citrate; *n.s*, no significance.

6. Biochemical pathways of apoptosis activation can be extracellular or intracellular, as well as caspase-dependent or mitochondrial-dependent [^24^](#_ENREF_24). The pan-caspase inhibitor Z-VAD-FMK (10 μM) did not affect the t-BHP-induced morphological changes, and failed to inhibit the t-BHP-induced decrease of cell viability (Figure S3A and B). The expressions of caspase-7, or -3, and the cleaved caspase-7, or -3 were also not altered by t-BHP treatment (Figure S3C-G). The IAP proteins function via direct interactions to inhibit the activity of several caspases, including caspase-7, and -3[^25-28^](#_ENREF_25). The t-BHP treatment did not affect the expression of XIAP, c-IAP1, and c-IAP2 (Figure S3H-J).


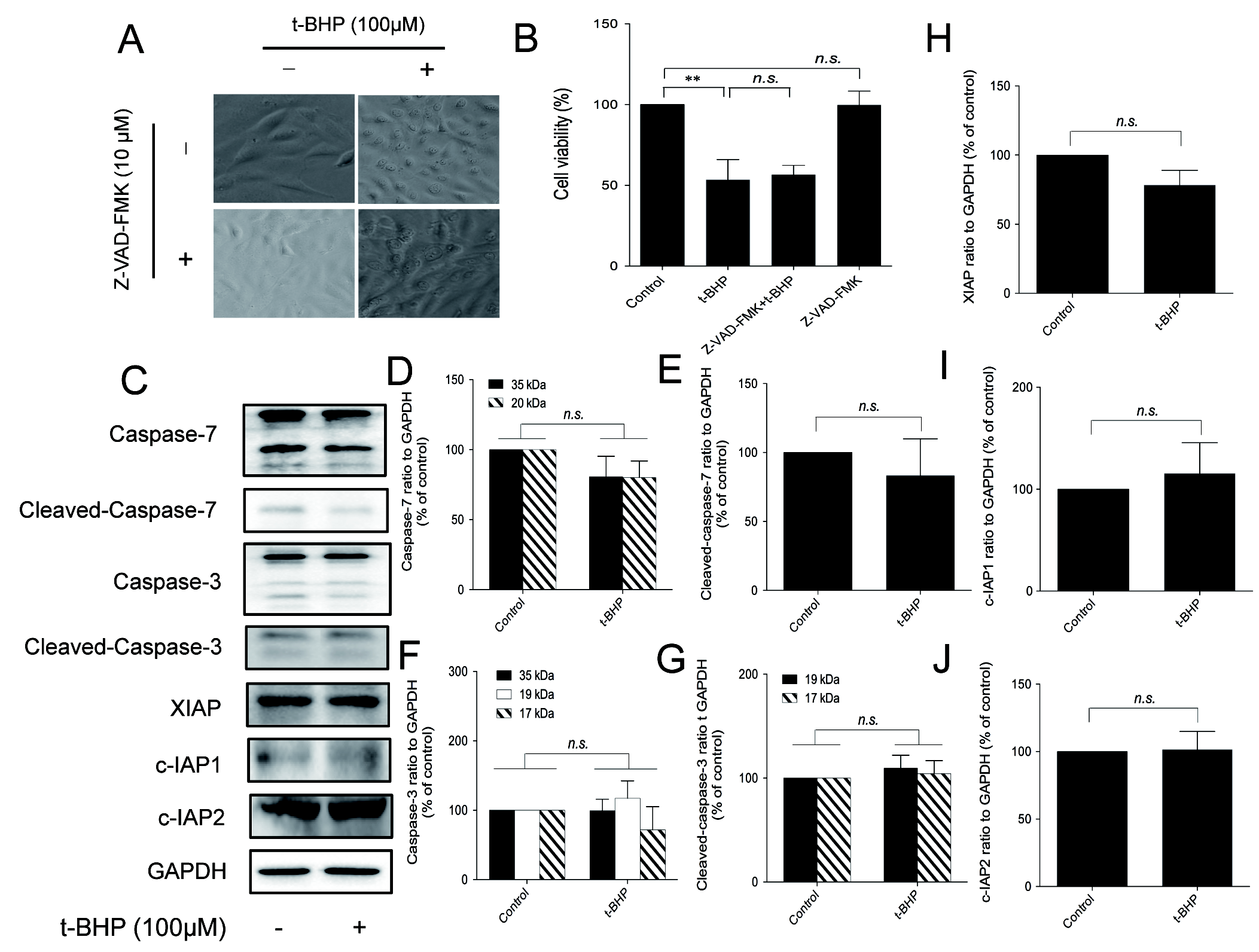


**Figure S3 Evidence against caspase-dependent apoptosis in PC12 cells after co-treatment with t-BHP**. PC12 cells were treated with t-BHP (100 μM) for 1 h with or without Z-VAD-FMK (10 μM) pretreatment for 24 h. Morphological changes (A) and the cell viability (B) were observed and determined by microscopy and the MTT assay respectively. The expression of caspase cascade proteins and IXP proteins (C-J) were detected by western blot analysis (The blots are displayed cropped and the full-length blots are included in the Supplementary Information file.). ^**^*P*＜0.01; *n.s*, no significance.

7. The cell morphology was changed in the t-BHP-treated group. CQ (30 μM) treatment did not affect the t-BHP-induced morphological alterations (Figure S4A and B). Specifically, CQ demonstrated no effects on the t-BHP-induced cell death. As shown in Figure S4, neither necrostatin-1 (Nec-1; 10 μM) nor necrostatin-1s (Nec-1s; 10 μM), which are both necroptosis inhibitors, could reverse the cell death and morphological changes induced by t-BHP (Figures S4C and E).


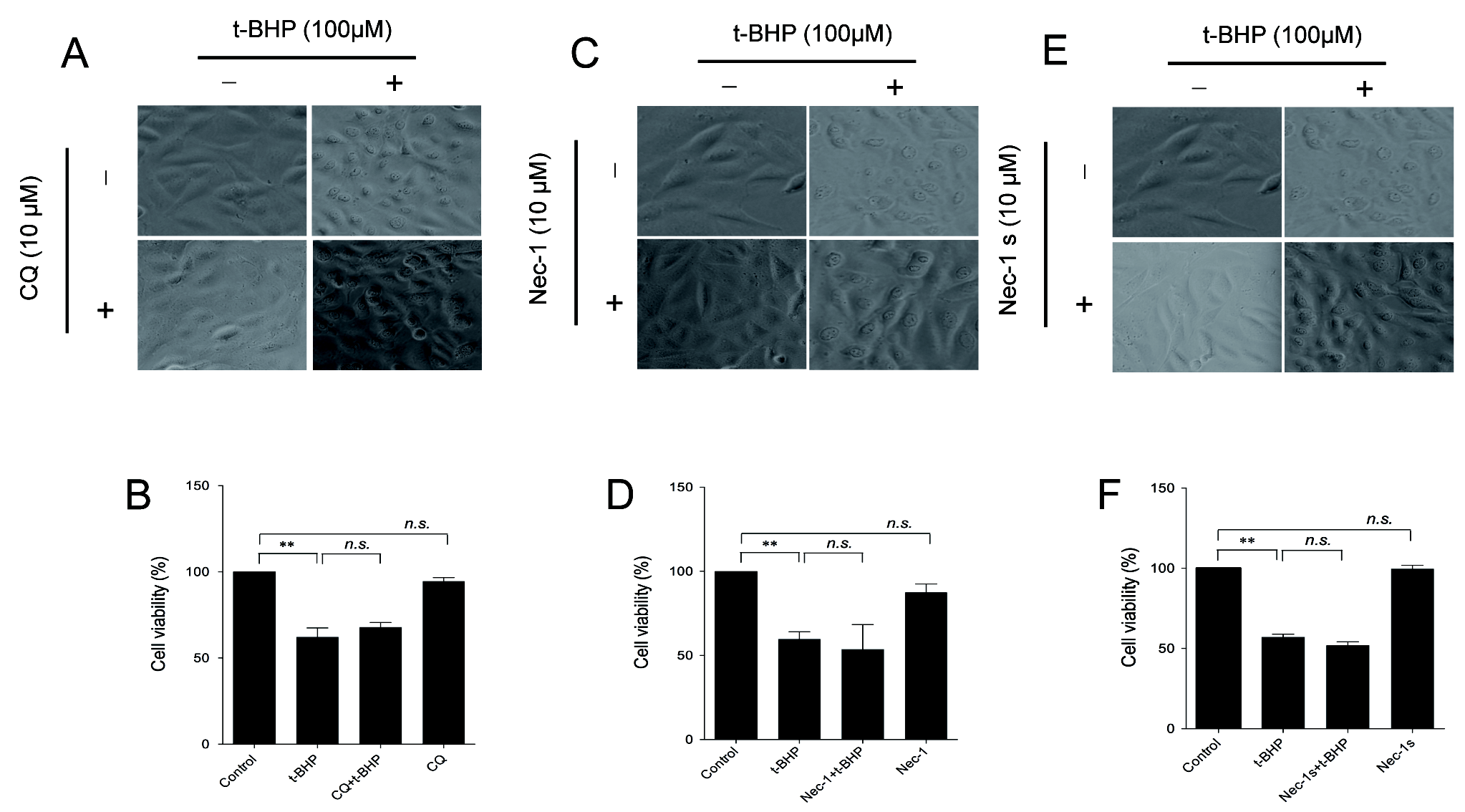


**Figure S4 Evidence against t-BHP-induced autophagy and necroptosis in PC12 cells**. PC12 cells were treated with t-BHP (100 μM) for 1 h with or without CQ (30 μM) pretreatment for 24 h. Morphological changes (A), (C) and (E) and the cell viability (B), (D) and (F) were observed and determined by microscopy and the MTT assay respectively. ^**^*P*＜0.01; CQ, chloroquine; *n.s*, no significance; Nec-1, necrostatin-1;Nec-1s, necrostain-1s; *n.s*, no significance.

8. As shown in Figure S5A-F, t-BHP treatment significantly increased the protein expression of *p*-JNK (Thr183/Tyr185) and *p*-ERK (Thr202/Tyr204), but had no effect on *p*-p38 mitogen-activated protein kinase (MAPK; Thr180/Tyr182). SP600125 (JNK inhibitor, 10 μM) and U0126 (ERK inhibitor, 10 μM) could partially reverse t-BHP-induced cell death, whereas SB202190 (p38 MAPK inhibitor, 20 μM) did not (Figures S5G, H, and I).


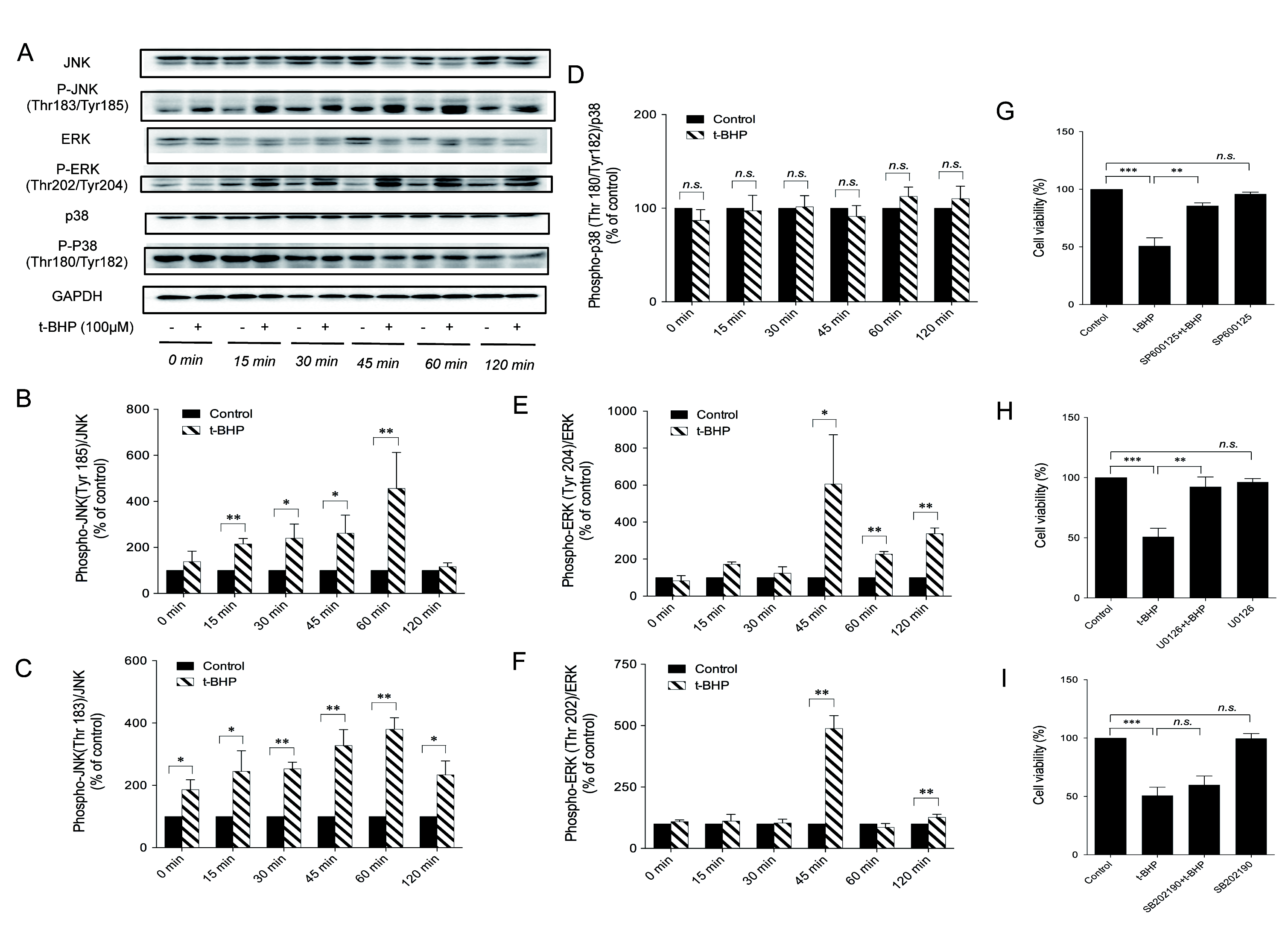


**Figure S5** **JNK1/2 and ERK1/2 were involved in t-BHP-induced cell death**. PC12 cells were treated with t-BHP (100 μM) for 1 h. The expression of MAPK cascade proteins (A)-(F) and cell viability (G)-(I) was determined by western blot analysis (The blots are displayed cropped and the full-length blots are included in the Supplementary Information file.) and MTT assay. ^**^*P*＜0.01; ^*^*P*＜0.05; *n.s*, no significance.

9. Another ERK inhibitor SCH772984 and JNK inhibitor JNK-IN-8 were used here to confirm the involvement of ERK and JNK in the cell death induced by t-BHP. As shown in Figure S6, we got the consistent results with the U0126 and SP600125, that SCH772984 and JNK-IN-8 increased the cell viability, the cell ATP content and the ratio of GSH/GSSG. Moreover, they increased the expression of Gpx4. All these results further confirmed the involvement of ERK signaling and JNK signaling in t-BHP induced cell death.


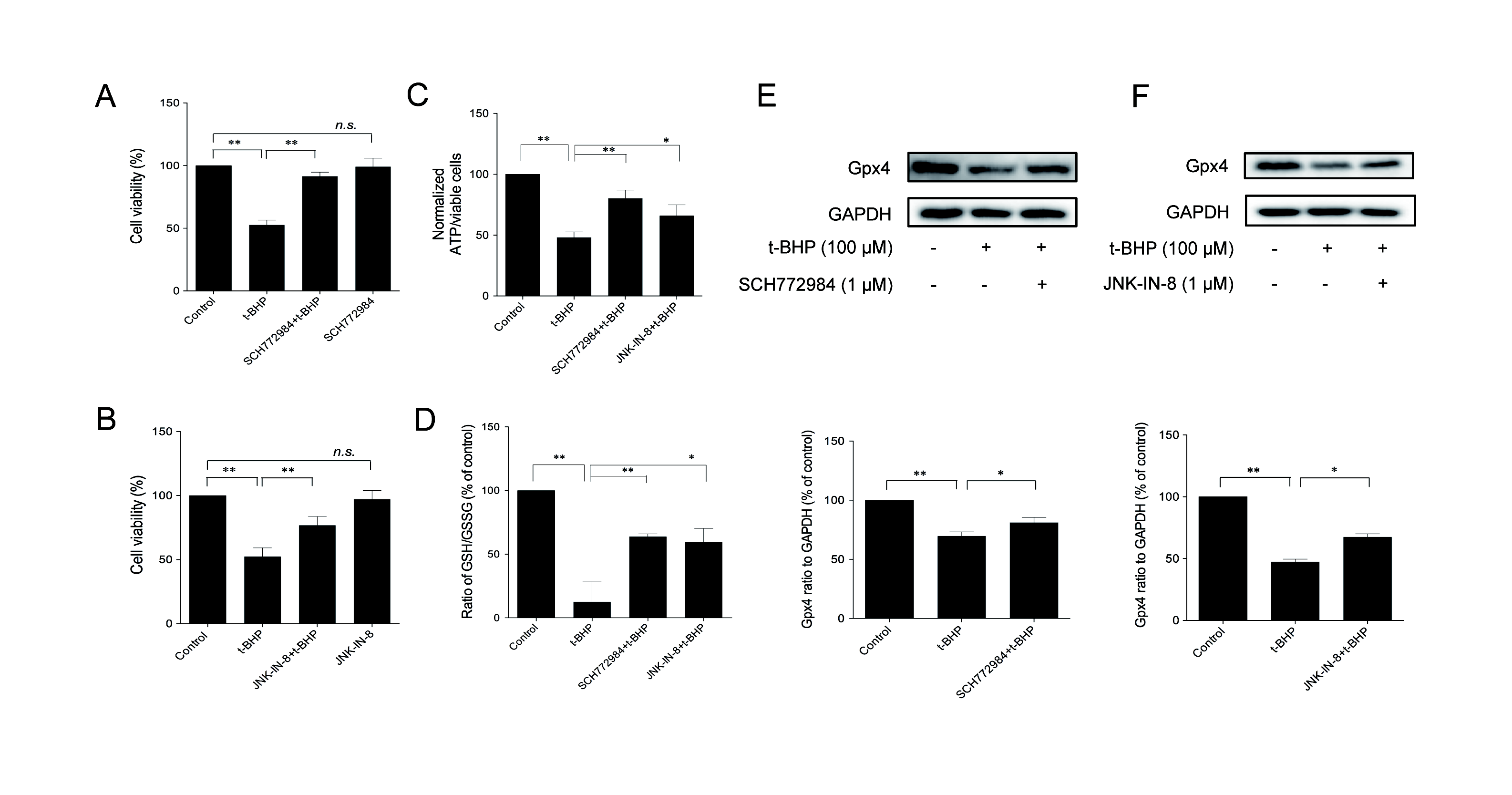


**Figure S6** SCH772984 and JNK-IN-8 rescued the cell death induced by t-BHP (100 μM for 1 h). PC12 cells were treated with t-BHP (100 μM) for 1 h with or without SCH772984 (1 μM) and JNK-IN-8 (1 μM) pretreatment for 24 h. Cell viability, ATP content and ratio of GSH/GSSG were detected by MTT assay and two commercial kits. The expression of Gpx4 was detected by western blot.

10. After treated with 100 μM t-BHP for 1 h, the PC12 cell morphology was rounding-up, and the quantified results revealed that PC12 rounding-up cells was significantly increased in t-BHP group, when compared with that in control group. Moreover, after treated with t-BHP, the expression of transferrin receptor (TFR), was also increased in the t-BHP treated group at the concentration of 100 μM and 200 μM.


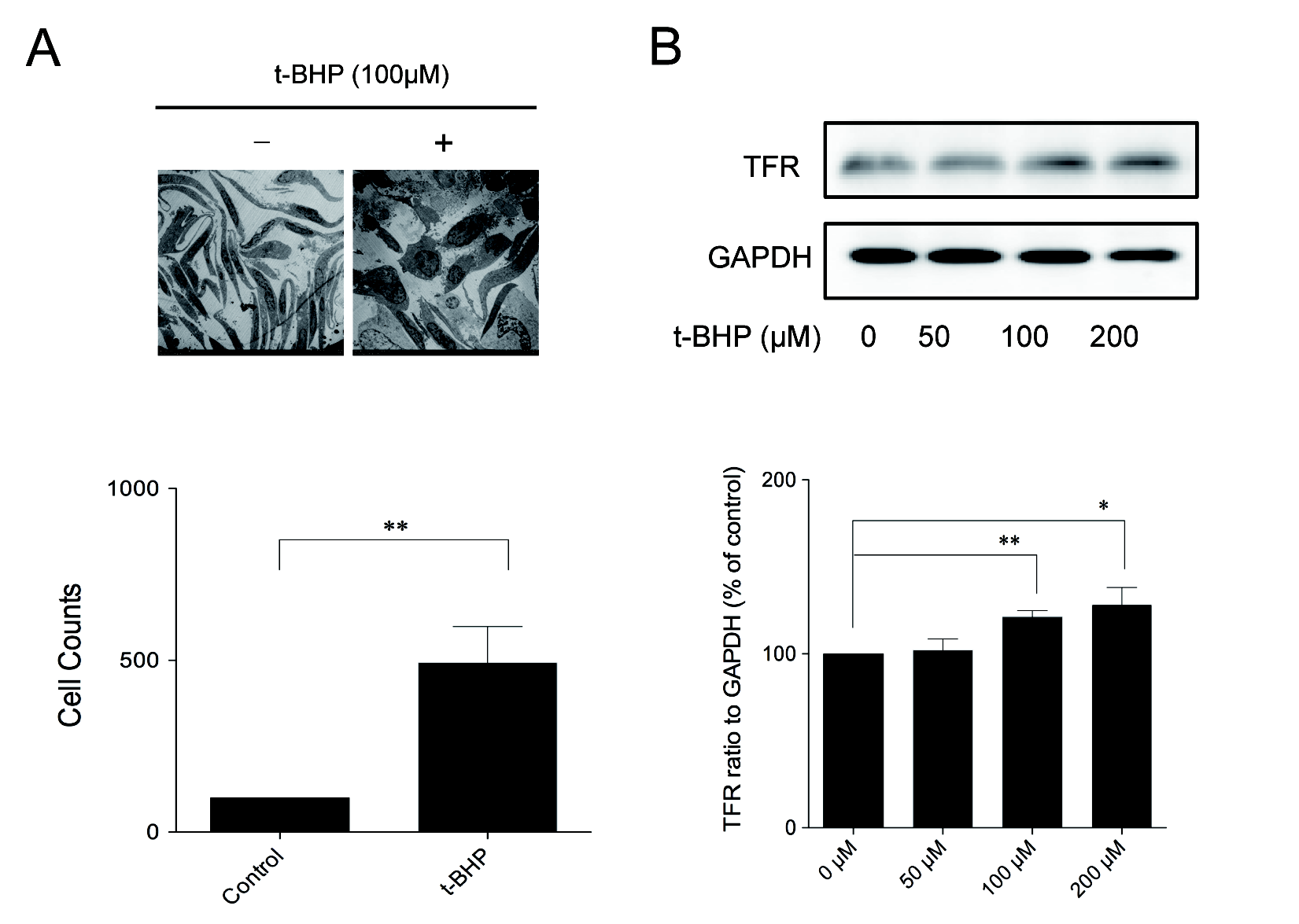


**Figure S7** Cell morphology and the expression of TFR was disturbed in PC12 cells after treated with t-BHP (100 μM) for 1h. Cell morphology images were observed by electron microscope (A). The expression of TFR was detected by western blot (B).
